# Integrating Pharmacokinetics Study, Network Analysis, and Experimental Validation to Uncover the Mechanism of Qiliqiangxin Capsule Against Chronic Heart Failure

**DOI:** 10.3389/fphar.2019.01046

**Published:** 2019-09-18

**Authors:** Yu Zhang, Mingdan Zhu, Fugeng Zhang, Shaoqiang Zhang, Wuxun Du, Xuefeng Xiao

**Affiliations:** ^1^School of Graduate, Tianjin University of Traditional Chinese Medicine, Tianjin, China,; ^2^The Second Affiliated Hospital, Tianjin University of Traditional Chinese Medicine, Tianjin, China; ^3^Department of Pharmacy, Tianjin Huanhu Hospital, Tianjin, China

**Keywords:** pharmacokinetics, network pharmacology, Qiliqiangxin capsule, chronic heart failure, HCMEC

## Abstract

**Objectives:** The purpose of this study was to propose an integrated strategy for investigating the mechanism of Qiliqiangxin capsule (QLQX) to treat chronic heart failure (CHF).

**Methods:** Pharmacokinetics analysis was performed to screen the active components of QLQX using high-performance liquid chromatography–tandem mass spectrometry techniques. We then constructed the component–target network between the targets of active components in QLQX and CHF using Cytoscape. A network analysis, including topological parameters, clustering, and pathway enrichment, was established to identify the hub targets and pathways. Finally, some of the predicted hub targets were validated experimentally in human cardiac microvascular endothelial cell (HCMEC).

**Results:** We identified 29 active components in QLQX, and 120 consensus potential targets were determined by the pharmacokinetics analysis and network pharmacology approach. Further network analysis indicated that 6 target genes, namely, *VEGFA*, *CYP1A1*, *CYP2B6*, *ATP1A1*, *STAT3*, and *STAT4*, and 10 predicted functional genes, namely, *KDR*, *FLT1*, *NRP2*, *JAK2*, *EGFR*, *IL-6*, *AHR*, *ATP1B1*, *JAK1*, and *HIF1A*, may be the primary targets regulated by QLQX for the treatment of CHF. Among these targets, *VEGFA*, *IL-6*, *p-STAT3*, and *p-JAK2* were selected for validation in the HCMEC. The results indicated that QLQX may inhibit inflammatory processes and promote angiogenesis in CHF *via* the JAK/STAT signaling pathway.

**Conclusions:** This study provides a strategy for understanding the mechanism of QLQX against CHF by combining pharmacokinetics study, network pharmacology, and experimental validation.

## Introduction

Traditional Chinese medicine, characterized by a holistic approach, has attracted increasing attention worldwide because of its satisfactory clinical efficacy (Fung and Linn, 2015; Zhang et al., 2015; Chao et al., 2017). Chinese herbal medicine, as the mainstay and principal form of traditional Chinese medicine practice, is composed of multiple herbal ingredients and hundreds of chemical compounds (Zhou et al., 2019). Hence, it is difficult to fully understand their effective ingredients and the mechanism of action which depends on the overall interactions among all the ingredients.

Qiliqiangxin capsule (QLQX), comprising 11 crude herbs, has been clinically used for treating chronic heart failure (CHF) in China, providing an effective alternative for treatments of CHF (Li et al., 2013; Tang and Huang, 2013; Sun J. et al., 2016). Chemically, flavonoids, saponins, cardiac glycosides, diterpene quinones, phenolic acids, diterpene alkaloids, and triterpenoids have been identified as the main constituents of QLQX (Yun et al., 2018). Recent pharmacological studies showed that QLQX performs various activities, including attenuating atrial structural remodeling (Tingting et al., 2019), improving endothelial cell function (Chen et al., 2015), and protecting cardiac myocytes and mitochondrial function (Zhang et al., 2013). However, the active compounds, potential targets, and pathways involved in these effects have not been systematically investigated.

Recently, integrated strategies based on bioinformatics, system biology, and high-throughput analytical techniques have emerged as a holistic and efficient tool to solve this issue (Ning et al., 2018; Xing et al., 2018; Huang et al., 2019). Network pharmacology gives us helpful tools to screen potential bioactive ingredients and understand the molecular mechanisms underlying the therapeutic effects by constructing component–target and target–disease networks (Li and Zhang, 2013; Yuan et al., 2017). In recent years, the network pharmacology-based method has been used to analyze the system-level mechanisms of mono- and poly-pharmacology, manifesting “multicompound, multitarget” characteristics (Engin et al., 2014; Park et al., 2018). However, there exist limitations in the insufficient accumulation and poor quality of traditional Chinese medicine-related data, and the nonreproducible nature of the existing data (Zhang R. et al., 2019). The information on the active constituents of Chinese herbal medicine is critical for accurate assessments of the network. Thus, the greater the reliability and correlation of the information on the active constituents, the more convincing will be the results of network research. It has also been accepted that the components absorbed into the blood that attain a certain blood concentration can produce pharmacodynamics effects and are considered pharmacologically active substances (Li et al., 2015; Mi et al., 2019). Therefore, pharmacokinetics study provides powerful evidence for determining the main active compounds absorbed into the blood and deciphering their process *in vivo*.

**Figure 1:**
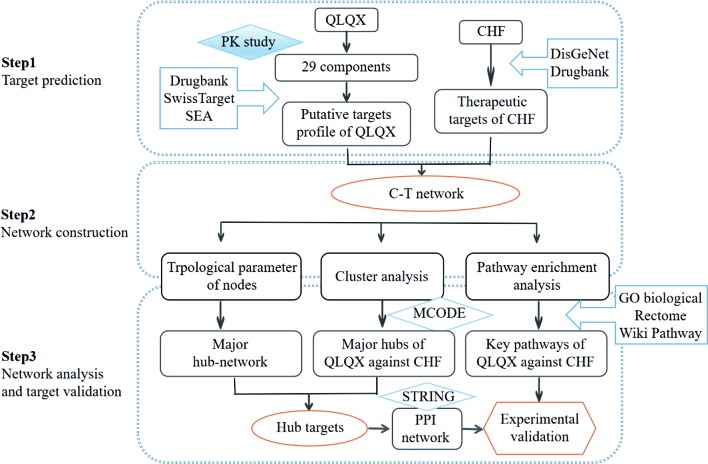
Flowchart of the study.

In the current study, a comprehensive method focusing on the main active compounds was used to illustrate the molecular mechanisms of QLQX by adopting pharmacokinetics study, network pharmacological analysis, and experimental validation. The flowchart is illustrated in **Figure 1**. Briefly, (1) a pharmacokinetics study was employed to determine the main active components of QLQX; (2) a component–target (C–T) network was established to visualize the synergistic interactions between the targets of the main active components and CHF; (3) topological parameters of the C–T network, clustering, and pathway enrichment analysis of major hubs of QLQX against CHF were used to pinpoint the hub targets and pathways; (4) the hub targets determined by topological parameters and clustering analysis were used to construct the protein–protein interaction (PPI) network; (5) some hub targets in the PPI network were experimentally validated in the human cardiac microvascular endothelial cell (HCMEC).

## Materials and Methods

### Reagents and Materials

The powder of QLQX was provided by Yiling Pharmaceutical Co. Ltd. (Shijiazhuang, China). Astragaloside, calycosin-7-glucoside, sinapine bisulfate, ginsenoside Rb1, Rb_2_, Rg_1_, Rg_3_, Rd, Re, Rf, and F_2_, salvianolic acid A, salvianolic acid B, danshensu, rosmarinic acid, protocatechuic acid, hydroxysafflor yellow A, formononetin, hesperidin, rutin, quercetin, mesaconitine, hypaconitine, benzoylaconine, benzoylmesaconine, and benzoylhypaconine were obtained from the National Institutes for Food and Drug Control (Beijing, China), and ginsenoside Rc, lithospermic acid, and aconitine were obtained from Shanghai Yuanye Biotechnology Co. Ltd. (Shanghai, China). High-performance liquid chromatography (HPLC)-grade methanol, acetonitrile, and ammonium acetate were obtained from Fisher Scientific International Inc. (Fair Lawn, NJ), and formic acid was obtained from CNW Technologies GmbH (Duesseldorf, Germany). HCMEC and complete growth medium [containing 90% high Dulbecco’s modified Eagle medium (H-DMEM), 10% fetal bovine serum (FBS), and penicillin/streptomycin] were purchased from BeNa Culture Collection (Beijing, China). Methylthiazolyldiphenyl-tetrazolium bromide (MTT) and bicinchoninic acid assay (BCA) protein assay kit were obtained from Beyotime (Shanghai, China). Rabbit antihuman monoclonal antibodies (*JAK2*, *phosphorylated-JAK2*, *STAT3*, *phosphorylated-STAT3*, *VEGFA*, and *β-actin*) and mouse antihuman monoclonal antibodies (*IL-6*) were provided by Beyotime (Shanghai, China). Rabbit antihuman monoclonal antibodies (*ADRB1*, *ADRB2*) were provided by Guidechem (Hangzhou, China).

### HPLC-MS/MS Method for Pharmacokinetics Study

High-performance liquid chromatography–tandem mass spectrometry (HPLC-MS/MS) analysis (Shimadzu Co., Japan) was performed to assess the pharmacokinetic properties of 29 components in QLQX according to the method described in our previous study (Zhang et al., 2018). Briefly, chromatographic separation was achieved on an Agilent ZOBRAX XDB-C_18_ column (4.6 mm × 50 mm, 3.5 µm) maintained at 40°C. The mobile phase consisted of water (A) and methanol (B) both containing 0.1% (*v/v*) formic acid for astragaloside, calycosin-7-glucoside, sinapine bisulfate, and ginsenoside Rb_1_, Rb_2_, Rg_1_, Rg_3_, Rc, Rd, Re, Rf, and F_2_ detection with a gradient elution. The mobile phase consisted of water (A) and acetonitrile (B) both containing 0.1% (*v/v*) formic acid for salvianolic acid A, salvianolic acid B, danshensu, lithospermic acid, rosmarinic acid, protocatechuic acid, hydroxysafflor yellow A, formononetin, hesperidin, rutin, and quercetin detection with a gradient elution. The mobile phase consisted of water containing 5 mM ammonium acetate (A) and methanol containing 0.1% formic acid (B) for aconitine, mesaconitine, hypaconitine, benzoylaconine, benzoylmesaconine, and benzoylhypaconine detection with a gradient elution. The flowrate was set at 0.45 ml/min, and the injection volume was 10 µl. The quantitative analysis was performed with multiple reaction monitoring in positive and negative ion modes.

### Plasma Sample Preparation

The powder of QLQX was diluted with 0.5% sodium carboxymethyl cellulose yielding a concentration of 0.13 g/ml suspension. Sprague–Dawley rats were intragastrically administered with QLQX suspension at 1.3 g/kg, and the blood samples were collected into a heparinized centrifuge tube at 5, 10, 20, 40, 60, 90, 120, 180, 240, 360, 480, 720, and 1,440 min after dosing *via* the postorbital venous plexus. Then, the whole blood was centrifuged at 12,000 rpm for 10 min, the supernatant was obtained, and stored at −80°C until analysis. The preparation of blood samples was conducted according to our previous research (Zhang et al., 2018). This study was carried out in accordance with the principles of the Basel Declaration and recommendations of guidelines of the National Institutes of Health. The protocol was approved by the Ethics Committee of Tianjin University of Traditional Chinese Medicine (Tianjin, China).

### Targets Fishing

The targets of active components in QLQX determined by pharmacokinetics analysis were obtained from three databases: DrugBank,^1^ Swiss Target Prediction,^2^ and Similarity Ensemble Approach (SEA).^3^ Known therapeutic targets of CHF were collected from the DrugBank database^1^ and DisGeNet database^4^.The keywords “chronic heart failure” and “congestive heart failure” were used, and the targets were human genes/proteins enrolled in this study.

### Network Construction and Topological Analysis

The C–T network was constructed using Cytoscape (Version 3.2.1) (Smoot et al., 2011). Four topological features (degree, betweenness centrality, average shortest path length, and closeness centrality) were analyzed using Network Analyzer (Assenov et al., 2008). The major hub network comprising putative major components and major targets was extracted by defining nodes with degrees higher than the average number of neighbors.

### Clustering Analysis

MCODE (Version 1.4.2) (Bader and Hogue, 2003) was employed to identify the major hubs of QLQX against CHF. MCODE analyzes the network based on the given parameter, and it assigns the weight to the vertex in local neighborhoods from the dense regions using vertex weighting, cluster prediction, and optimal postprocessing. Finally, we defined the hub targets by considering the results of the topological analysis and clustering analysis. To obtain the proteins interacting with the hub targets, the STRING^5^ database was used, and the association score ≥0.9 was considered the highest confidence.

### Pathway Enrichment Analysis

ClueGO (Version 2.3.2) (Rubinov and Sporns, 2010) was utilized to analyze the representative biological processes and pathways associated with QLQX against CHF. All targets obtained from the C–T network were imported. GO biological process, Reactome pathway, and Wiki pathway were selected from the ClueGO setting panel, and a two-sided hypergeometric test with *p* ≤ 0.01 significance level for biological process analysis and a *p* ≤ 0.05 significance level for pathway analysis was used.

### Cell Culture and Treatments

HCMECs were maintained in complete growth medium (90% H-DMEM, 10% FBS, and penicillin/streptomycin). All cells were cultured at 37°C in a humidified atmosphere containing 5% CO_2_. After three or four passages, the HCMECs were digested with 0.25% trypsin and adjusted to a density of 5 × 10^4^ cells/ml and 1 × 10^5^ cells/ml for cell viability assay and Western blot analysis, respectively. The powder of QLQX was accurately weighed and dissolved in complete growth medium to various concentrations (0.15 and 0.3 mg/ml). The cells used for cell viability assay were seeded on 96-well plates in 100 µl of complete growth medium (0.15 or 0.3 mg/ml QLQX were added) for 24, 48, or 72 h and were then treated with 10 mM Hcy for another 24 h. The cells used for Western blot analysis were divided into the following groups: control, model, positive-captopril (CA), QLQX-low (-L), and QLQX-high (-H). Cells in the control group were cultured without any treatments. In the model group, the HCMECs (1 × 10^5^/ml) were cultured in 3 ml complete growth medium (10 mM Hcy was added) for 24 h. In the CA, -L, and -H groups, cells were pretreated with 0.075 mg/ml captopril, 0.15 mg/ml QLQX, and 0.3 mg/ml QLQX for 48 h, respectively, and then cultured in complete growth medium (10 mM Hcy was added) for a further 24 h.

### Cell Viability Assay

Cell viability of the HCMECs was evaluated using the MTT assay. Cells were seeded on 96-well plates with a density of 5 × 10^4^ cells/ml in 100 µl of complete growth medium (0.15 or 0.3 mg/ml QLQX were added) for 24, 48, or 72 h and were then treated with 10 mM Hcy for 24 h. Following treatment, 10 μl of MTT (5 mg/ml) was added to each well. After 4 h, the culture medium was removed, and 100 μl of dimethyl sulfoxide (Beyotime, Shanghai, China) was added. The absorbance was measured at 570 nm using a microplate reader (Infinite M200 Pro, Tecan, Switzerland), and the cell viability was expressed as a percentage of the value of the untreated group.

### Western Blot Analysis

HCMECs (1 × 10^5^ cells/ml) were seeded on 60 × 20-mm dishes in 3 ml of complete growth medium for 24 h. After treatment, the HCMECs were scraped off and washed twice with cold phosphate-buffered saline. The cells were solubilized by radioimmunoprecipitation assay lysis buffer (Beyotime, China) containing 1% phenylmethylsulphonyl fluoride (Beyotime, China) and 1% phosphatase inhibitor for 20 min on ice. Whole-cell lysates were clarified by centrifuging at 12,000 rpm for 10 min at 4°C, and the supernatants were collected. Protein concentrations were determined by the BCA protein assay. The protein samples were mixed with sodium dodecyl sulfate polyacrylamide gel electrophoresis sample loading buffer (Beyotime, China) and boiled at 100°C for 5 min. Equal concentrations of protein (2 mg/ml) were separated by electrophoresis on 10% sodium dodecyl sulphate polyacrylamide gels and were transferred onto polyvinylidene difluoride membranes. These membranes were soaked in 5% skimmed milk, and dissolved with TBST buffer (Tris Buffer Saline supplemented with 0.1% Tween-20) 5 times (15 min each time) to block nonspecific binding sites. The membranes were then incubated overnight at 4°C with the primary antibodies (*VEGFA*, *STAT3*, *p-STAT3*, *JAK2*, *p-JAK2*, *IL-6*, *ADRB1*, and *ADRB2*). After washing with TBST, the membranes were incubated for 2 h at room temperature with horseradish peroxidase-labeled secondary antibodies. After rewashing with TBST, the membranes were scanned using a fluorescent scanner (Odyssey CLX, Gene Company Limited, USA). Band intensity was analyzed using ImageJ software (National Institutes of Health, Bethesda, MD, USA).

### Statistical Analysis

Data were presented as mean ± SD and analyzed using GraphPad Prism 6 (GraphPad Software, Inc., La Jolla, CA, USA). One-way analysis of variance (ANOVA) was used, and *p*-value < 0.05 was considered statistically significant.

## Results

### Pharmacokinetics Study of 29 Components in QLQX by HPLC-MS/MS

**Figure 2:**
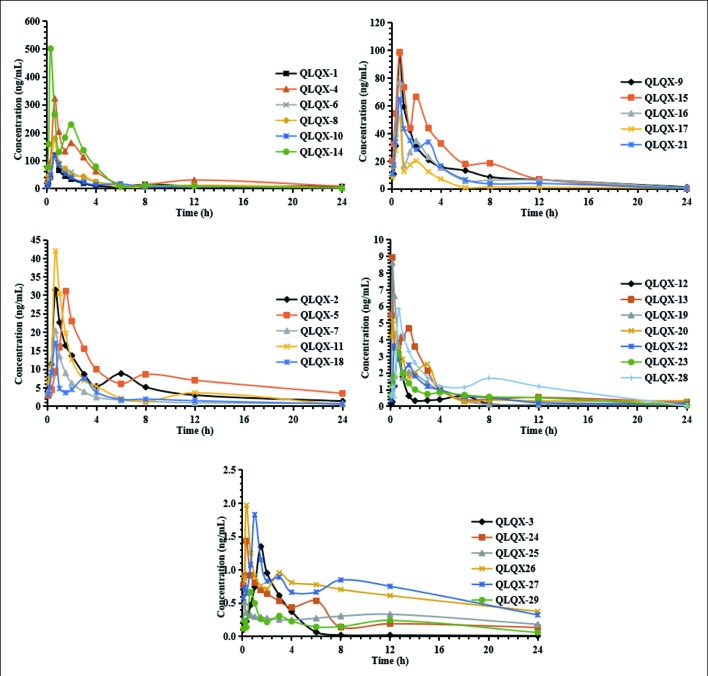
Mean concentration-time profiles of astragaloside (QLQX-1), calycosin-7-glucoside (QLQX-2), sinapine bisulfate (QLQX-3), ginsenoside Rb_1_ (QLQX-4), Rb_2_ (QLQX-5), Rg_1_ (QLQX-6), Rg_3_ (QLQX-7), Rc (QLQX-8), Rd (QLQX-9), Re (QLQX-10), Rf (QLQX-11), F_2_ (QLQX-12), salvianolic acid A (QLQX-13), salvianolic acid B (QLQX-14), danshensu (QLQX-15), lithospermic acid (QLQX-16), rosmarinic acid (QLQX-17), protocatechuic acid (QLQX-18), hydroxysafflor yellow A (QLQX-19), formononetin (QLQX-20), hesperidin (QLQX-21), rutin (QLQX-22), quercetin (QLQX-23), aconitine (QLQX-24), mesaconitine (QLQX-25), hypaconitine (QLQX-26), benzoylaconine (QLQX-27), benzoylmesaconine (QLQX-28), benzoylhypaconine (QLQX-29).

Pharmacokinetic properties of 29 components in QLQX (astragaloside, calycosin-7-glucoside, sinapine bisulfate, ginsenoside Rb_1_, Rb_2_, Rg_1_, Rg_3_, Rc, Rd, Re, Rf, F_2_, salvianolic acid A, salvianolic acid B, danshensu, lithospermic acid, rosmarinic acid, protocatechuic acid, hydroxysafflor yellow A, formononetin, hesperidin, rutin, quercetin, aconitine, mesaconitine, hypaconitine, benzoylaconine, benzoylmesaconine, and benzoylhypaconine) were studied using the HPLC-MS/MS method according to the method in our previous study (Zhang et al., 2018). The pharmacokinetic properties of 29 components are shown in **Table 1**, and the mean concentration–time curves are shown in **Figure 2**. These 29 components of QLQX detected in the blood of the rats and with appropriate pharmacokinetic properties were determined to be the main active components and used to construct the C–T network.

**Table 1:** Pharmacokinetic properties of 29 components in QLQX determined by HPLC-MS/MS.

No.	Compounds	Polarity	Parent (m/z)	Daughter (m/z)	Pharmacokinetic parameters				
					C_max_	T_max_	t_1/2_	AUC*_0∼t_*	AUC*_0∼∞_*
1	Astragaloside	Positive	807.5	627.3	120.46 ± 45.03	0.67 ± 0.00	5.74 ± 0.78	320.92 ± 79.10	369.14 ± 103.83
2	Calycosin-7-glucoside	Positive	447.2	285.2	31.49 ± 12.00	0.67 ± 0.06	6.05 ± 3.34	103.26 ± 47.63	120.71 ± 50.71
3	Sinapine bisulfate	Positive	310.2	251.2	1.35 ± 0.62	1.50 ± 0.00	3.52 ± 0.17	3.98 ± 1.21	8.76 ± 2.85
4	Ginsenoside Rb_1_	Positive	1,131.7	365.2	323.00 ± 91.00	0.67 ± 0.00	9.16 ± 3.18	590.15 ± 257.83	713.49 ± 270.66
5	Ginsenoside Rb_2_	Positive	1,107.8	335.0	31.25 ± 9.89	1.50 ± 0.06	10.11 ± 4.53	143.73 ± 35.67	307.99 ± 79.92
6	Ginsenoside Rg_1_	Positive	823.5	643.5	113.44 ± 48.22	0.67 ± 0.00	8.69 ± 3.17	362.17 ± 118.25	422.31 ± 180.63
7	Ginsenoside Rg_3_	Positive	807.6	364.9	20.49 ± 7.16	0.67 ± 0.24	5.03 ± 2.02	67.13 ± 27.93	89.81 ± 34.43
8	Ginsenoside Rc	Positive	1,101.6	789.5	180.00 ± 70.00	0.67 ± 0.00	4.32 ± 2.10	432.71 ± 178.66	493.04 ± 233.70
9	Ginsenoside Rd	Positive	969.5	789.6	97.49 ± 31.00	0.67 ± 0.00	5.90 ± 1.14	204.66 ± 87.30	257.92 ± 101.52
10	Ginsenoside Re	Positive	969.6	789.6	118.94 ± 52.82	0.67 ± 0.06	7.33 ± 3.45	268.84 ± 103.24	310.95 ± 147.36
11	Ginsenoside Rf	Positive	823.5	365.2	43.03 ± 12.49	0.67 ± 0.00	6.12 ± 3.53	130.27 ± 27.91	177.68 ± 68.95
12	Ginsenoside F_2_	Positive	807.5	627.7	3.83 ± 1.23	0.67 ± 0.00	6.23 ± 2.89	12.73 ± 3.62	18.64 ± 5.28
13	Salvianolic acid A	Negative	493.1	294.9	8.96 ± 2.90	0.17 ± 0.00	7.50 ± 4.92	18.59 ± 7.91	21.40 ± 7.36
14	Salvianolic acid B	Negative	717.3	519.2	502.00 ± 68.43	0.33 ± 0.14	5.32 ± 1.32	818.47 ± 249.00	980.47 ± 112.07
15	Danshensu	Negative	196.9	134.8	98.60 ± 23.66	0.67 ± 0.00	3.52 ± 0.42	290.92 ± 68.38	390.90 ± 66.50
16	Lithospermic acid	Negative	537.4	493.2	77.25 ± 19.90	0.67 ± 0.00	4.80 ± 0.89	173.49 ± 38.57	216.94 ± 27.39
17	Rosmarinic acid	Negative	358.9	160.9	52.16 ± 5.96	0.67 ± 0.00	5.28 ± 1.35	78.65 ± 35.72	96.69 ± 24.81
18	Protocatechuic acid	Negative	152.9	108.8	17.00 ± 7.26	0.67 ± 0.00	8.38 ± 1.02	44.15 ± 18.02	61.03 ± 10.51
19	Hydroxysafflor yellow A	Negative	611.2	491.0	8.62 ± 3.51	0.17 ± 0.00	5.03 ± 2.00	15.63 ± 4.42	21.99 ± 6.25
20	Formononetin	Negative	266.9	251.7	5.17 ± 1.13	0.17 ± 0.00	5.07 ± 2.13	14.70 ± 5.48	18.04 ± 6.69
21	Hesperidin	Negative	609.2	300.9	64.63 ± 23.49	0.67 ± 0.00	3.34 ± 0.42	154.32 ± 66.50	187.77 ± 34.98
22	Rutin	Negative	609.2	299.6	3.63 ± 1.25	0.33 ± 0.00	5.33 ± 1.27	12.29 ± 2.47	16.92 ± 5.21
23	Quercetin	Negative	301.1	150.7	3.23 ± 1.26	0.67 ± 0.00	5.04 ± 0.98	10.96 ± 3.42	15.03 ± 5.28
24	Aconitine	Positive	646.4	586.4	1.44 ± 0.68	0.33 ± 0.00	18.35 ± 5.44	7.03 ± 2.31	18.16 ± 6.87
25	Mesaconitine	Positive	632.3	572.3	0.52 ± 0.16	0.17 ± 0.06	6.23 ± 1.23	3.95 ± 1.02	5.13 ± 2.32
26	Hypaconitine	Positive	616.3	556.3	1.97 ± 0.33	0.33 ± 0.00	8.01 ± 2.39	7.89 ± 2.57	13.85 ± 6.67
27	Benzoylaconine	Positive	604.2	554.3	1.83 ± 0.33	1.00 ± 0.00	18.63 ± 7.20	8.63 ± 2.39	25.94 ± 9.03
28	Benzoylmesaconine	Positive	590.2	540.3	5.84 ± 0.61	0.67 ± 0.11	8.23 ± 2.02	20.13 ± 6.27	35.34 ± 11.22
29	Benzoylhypaconine	Positive	574.2	542.3	0.67 ± 0.32	0.67 ± 0.00	12.88 ± 3.47	4.03 ± 1.03	7.80 ± 3.84

### Compound–Target Network Construction

**Figure 3:**
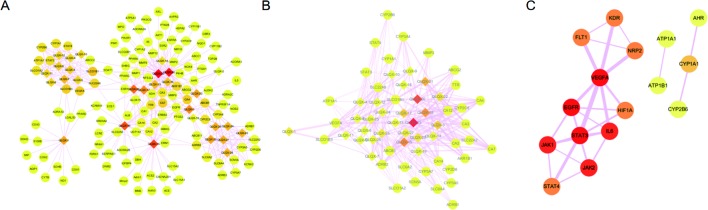
The networks associating with QLQX in the treatment of CHF. **(A)** The component-target network consisted of 149 nodes and 446 edges. **(B)** The major hub network define by nodes with degree higher the average number of neighbors (5.830). **(C)** The PPI network of hub targets obtained from STRING database and constructed by Cytoscape. The ellipse nodes represent targets, and the diamond nodes represent active components in QLQX, the widths of the edges represent the order of the edge betweenness and the colors of the nodes are illustrated from red to yellow in descending order of degree values.

Totally, 1,288 targets were found for the 29 components using Drugbank, Swiss Target Prediction, and SEA databases. The detailed target information for the 29 components in QLQX is shown in **Supplementary Table S1**. A total of 812 candidate targets of CHF were obtained from DisGeNet and Drugbank databases after removing redundant entries (**Supplementary Table S2**). Taking the intersection of the 1,288 putative targets of the 29 components in QLQX and the 812 candidate targets associated with CHF, a total of 120 consensus targets were collected as potential therapeutic targets of QLQX against CHF and used to establish the C–T network. As a result, a C–T network comprising 29 components and 120 consensus targets was constructed using Cytoscape. As shown in **Figure 3A**, the network comprised 149 nodes (29 components and 120 targets) and 446 edges.

### Network Topological Analysis

Network analyzer was used to calculate four topological features (degree, betweenness centrality, average shortest path length, and closeness centrality) of the nodes of the C–T network. Nodes with degrees higher than the average number of neighbors (5.830) were identified and extracted as the major hub networks (**Figure 3B**). Among these, 32 major targets with higher degree, closeness centrality, betweenness centrality, and lower average shortest path length were recognized as the major putative targets, and the results are shown in **Table 2**. Twenty-eight components were identified as the main active components (**Table 3**), and quercetin had the highest degree and betweenness centrality of 46 and 0.35, respectively, thereby indicating that quercetin has the most important position in the network.

**Table 2:** The topological parameters of 32 major targets.

Swiss prot	Genes/proteins	Description	Degree	Betweenness centrality	Average shortest path length	Closeness centrality
Q9Y6L6	SLCO1B1	Solute carrier organic anion transporter family member 1B1	12	0.09496758	2.69078947	0.37163814
Q9NPD5	SLCO1B3	Solute carrier organic anion transporter family member 1B3	11	0.04862563	3.28289474	0.30460922
P43166	CA7	Carbonic anhydrase 7	11	0.04690937	2.40131579	0.41643836
P22748	CA4	Carbonic anhydrase 4	11	0.04164377	2.45394737	0.4075067
P15692	VEGFA	Vascular endothelial growth factor A	11	0.11260667	2.79605263	0.35764706
Q8TCC7	SLC22A8	Solute carrier family 22 member 8	10	0.03755913	3.23026316	0.3095723
P15121	AKR1B1	Aldo-keto reductase family 1 member B	10	0.02861805	2.70394737	0.36982968
P08183	ABCB1	Multidrug resistance protein 1	10	0.06692038	2.67763158	0.37346437
Q14765	STAT4	Signal transducer and activator of transcription 4	9	4.74E−04	4.08552632	0.24476651
P46721	SLCO1A2	Solute carrier organic anion transporter family member 1A2	9	4.74E−04	4.08552632	0.24476651
P40763	STAT3	Signal transducer and activator of transcription 3	9	4.74E−04	4.08552632	0.24476651
P20813	CYP2B6	Cytochrome P450 2B6	9	4.74E−04	4.08552632	0.24476651
P10632	CYP2C8	Cytochrome P450 2C8	9	0.05421697	2.75657895	0.3627685
P05023	ATP1A1	Sodium/potassium-transporting ATPase subunit alpha-1	9	4.74E−04	4.08552632	0.24476651
P04798	CYP1A1	Cytochrome P450 1A1	9	4.74E−04	4.08552632	0.24476651
P02766	TTR	Transthyretin	9	0.02882135	2.53289474	0.39480519
P08684	CYP3A4	Cytochrome P450 3A4	8	0.01906502	3.15131579	0.31732777
P07550	ADRB2	Beta-2 adrenergic receptor	8	0.04366511	3.20394737	0.31211499
P07451	CA3	Carbonic anhydrase 3	8	0.03416389	2.45394737	0.4075067
Q9UNQ0	ABCG2	ATP binding cassette subfamily G member 2 (Junior blood group)	7	0.0203742	2.74342105	0.36450839
Q9ULX7	CA14	Carbonic anhydrase 14	7	0.02057174	2.625	0.38095238
P31645	SLC6A4	Solute carrier family 6 member 4	7	0.0021531	3.83552632	0.26072041
P23975	SLC6A2	Solute carrier family 6 member 2	7	0.0021531	3.83552632	0.26072041
O43570	CA12	Carbonic anhydrase 12	7	0.01886671	2.66447368	0.37530864
Q14524	SCN5A	Sodium voltage-gated channel alpha subunit 5	6	1.51E−04	4.09868421	0.24398074
P24462	CYP3A7	Cytochrome P450 3A7	6	1.51E−04	4.09868421	0.24398074
P20815	CYP3A5	Cytochrome P450 3A5	6	1.51E−04	4.09868421	0.24398074
P10635	CYP2D6	Cytochrome P450 2D6	6	1.51E−04	4.09868421	0.24398074
P08588	ADRB1	Adrenoceptor beta 1	6	1.51E−04	4.09868421	0.24398074
P08254	MMP3	Matrix metallopeptidase 3	6	0.00514916	2.95394737	0.33853007
P00918	CA2	Carbonic anhydrase 2	6	0.01628126	2.67763158	0.37346437
O15244	SLC22A2	Solute carrier family 22 member 2	6	1.51E−04	4.09868421	0.24398074

**Table 3:** The topological parameters of 28 main active components.

No.	Compounds	Degree	Betweenness centrality	Average shortest path length	Closeness centrality
QLQX-23	Quercetin	46	0.34943416	2.21052632	0.45238095
QLQX-20	Formononetin	37	0.20139204	2.47368421	0.40425532
QLQX-15	Danshensu	27	0.14738861	2.85526316	0.35023041
QLQX-18	Protocatechuic acid	25	0.14911094	2.65789474	0.37623762
QLQX-21	Hesperidin	22	0.10590127	2.71052632	0.36893204
QLQX-3	Sinapine bisulfate	18	0.06332468	2.94736842	0.33928571
QLQX-17	Rosmarinic acid	18	0.05489072	2.96052632	0.33777778
QLQX-22	Rutin	15	0.03964651	2.98684211	0.33480176
QLQX-1	Astragaloside	15	0.13507408	3.44736842	0.29007634
QLQX-29	Benzoylhypaconine	15	0.02499921	3.17105263	0.3153527
QLQX-27	Benzoylaconine	14	0.0210363	3.18421053	0.31404959
QLQX-26	Hypaconitine	14	0.0210363	3.18421053	0.31404959
QLQX-7	Ginsenoside Rg_3_	13	0.03221083	3.19736842	0.3127572
QLQX-13	Salvianolic acid A	13	0.0102585	3.19736842	0.3127572
QLQX-28	Benzoylmesaconine	13	0.01850138	3.19736842	0.3127572
QLQX-25	Mesaconitine	13	0.01786266	3.19736842	0.3127572
QLQX-24	Aconitine	13	0.01786266	3.19736842	0.3127572
QLQX-16	Lithospermic acid	12	0.00961587	3.21052632	0.31147541
QLQX-8	Ginsenoside Rc	11	0.01141274	3.22368421	0.31020408
QLQX-4	Ginsenoside Rb_1_	11	0.01141274	3.22368421	0.31020408
QLQX-9	Ginsenoside Rd	10	0.0092025	3.23684211	0.30894309
QLQX-6	Ginsenoside Rg_1_	10	0.0092025	3.23684211	0.30894309
QLQX-5	Ginsenoside Rb_2_	10	0.0092025	3.23684211	0.30894309
QLQX-12	Ginsenoside F_2_	10	0.0092025	3.23684211	0.30894309
QLQX-11	Ginsenoside Rf	10	0.0092025	3.23684211	0.30894309
QLQX-10	Ginsenoside Re	10	0.0092025	3.23684211	0.30894309
QLQX-14	Salvianolic acid b	9	0.0045723	3.25	0.30769231
QLQX-19	Hydroxysafflor yellow a	7	0.01639755	3.09210526	0.32340426

### Clustering Analysis

MCODE was used to identify the major hubs of QLQX against CHF, which generated a well-organized cluster containing 17 nodes (*SLC22A8*, *SLCO1B3*, QLQX-9, QLQX-11, QLQX-5, QLQX-8, *CYP1A1*, QLQX-12, QLQX-6, *ATP1A1*, *VEGFA*, *CYP2B6*, *STAT3*, *SLCO1A2*, QLQX-10, QLQX-4, and *STAT4*). Taking the intersection of the topological analysis and clustering analysis results, a total of nine consensus targets were collected to be hub targets of QLQX against CHF, namely, *SLC22A8*, *SLCO1B3*, *CYP1A1*, *ATP1A1*, *VEGFA*, *CYP2B6*, *STAT3*, *SLCO1A2*, and *STAT4*. Then, the nine consensus targets were used as hub targets and submitted to STRING to generate the proteins interacting with these hub targets. The STRING database provides both experimental and predicted interaction information and provides a probabilistic association confidence score by calculation. As shown in **Table 4**, six target genes, namely, *VEGFA*, *CYP1A1*, *CYP2B6*, *ATP1A1*, *STAT3*, and *STAT4*, and 10 predicted functional genes, namely, *KDR*, *FLT1*, *NRP2*, *JAK2*, *EGFR*, *IL-6*, *AHR*, *ATP1B1*, *JAK1*, and *HIF1A*, having the highest confidence with an association score ≥0.9 were imported into Cytoscape 3.2.1 (**Figure 3C**). The network analysis tool was used to analyze the PPI network and targets with a higher degree played an important role in central correlation. The results are shown in **Table 5**.

**Table 4:** The proteins interacting of hub targets.

Node 1	Interaction	Node 2	Score
VEGFA	pp	KDR	0.999
VEGFA	pp	FLT1	0.999
VEGFA	pp	NRP2	0.998
JAK2	pp	STAT3	0.998
EGFR	pp	STAT3	0.998
CYP1A1	pp	AHR	0.997
JAK1	pp	STAT3	0.997
ATP1A1	pp	ATP1B1	0.997
IL6	pp	STAT3	0.997
VEGFA	pp	HIF1A	0.996
VEGFA	pp	STAT3	0.994
NRP2	pp	FLT1	0.988
FLT1	pp	KDR	0.988
HIF1A	pp	STAT3	0.986
STAT4	pp	JAK2	0.984
NRP2	pp	KDR	0.983
STAT4	pp	JAK1	0.983
VEGFA	pp	EGFR	0.979
IL6	pp	JAK2	0.975
HIF1A	pp	EGFR	0.973
IL6	pp	JAK1	0.968
JAK1	pp	EGFR	0.966
VEGFA	pp	IL6	0.959
JAK2	pp	JAK1	0.943
STAT4	pp	STAT3	0.938
JAK2	pp	EGFR	0.928
CYP1A1	pp	CYP2B6	0.928
IL6	pp	EGFR	0.921

**Table 5:** The topological parameters of PPI network.

Genes/proteins	Degree	Betweenness centrality	Average shortest path length	Closeness centrality
VEGFA	7	0.47407407	1.3	0.76923077
STAT3	7	0.21481481	1.3	0.76923077
EGFR	6	0.08888889	1.4	0.71428571
JAK2	5	0.01481481	1.8	0.55555556
JAK1	5	0.01481481	1.8	0.55555556
IL6	5	0.05925926	1.5	0.66666667
KDR	3	0	2	0.5
FLT1	3	0	2	0.5
NRP2	3	0	2	0.5
HIF1A	3	0	1.7	0.58823529
STAT4	3	0	2	0.5
CYP1A1	2	1	1	1
AHR	1	0	1.5	0.66666667
ATP1A1	1	0	1	1
ATP1B1	1	0	1	1
CYP2B6	1	0	1.5	0.66666667

### Pathway Enrichment Analysis

**Figure 4:**
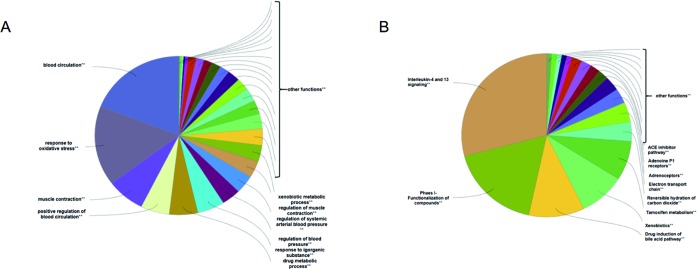
ClueGO analysis of the candidate targets. **(A)** Representative biological process among 120 candidate targets. **(B)** Representative reactome and Wiki pathway analysis amng 120 candidate targets. Only top ten concerned biological processes and pathways were shown in figures.

**Figure 5:**
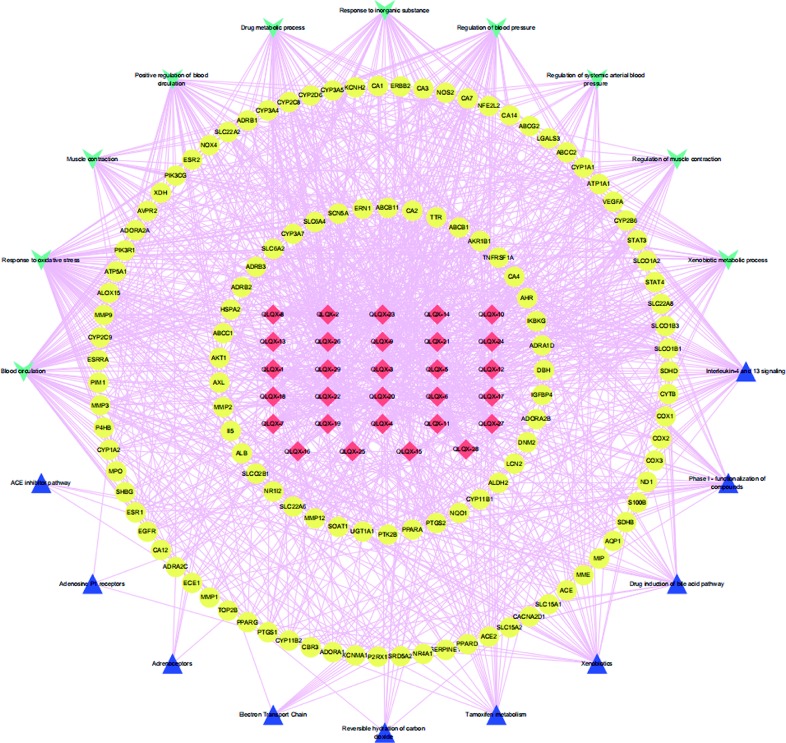
The component-target-pathway/biological process network. The red diamond nodes represent the 29 active components in QLQX, the yellow ellipse nodes represent 120 putative targets associated with QLQX in the treatment of CHF, the wathet V nodes represent top ten biological processes and the mazarine triangle nodes represent top ten pathways related the 20 putative.

To analyze the representative biological processes and pathways associated with the 120 targets of the C–T network, GO biological, Reactome, and Wiki pathway analysis were used to explore the potential biological processes and pathways affected by QLQX through analysis of the 120 targets. The biological processes were ranked by their nominal *P* values with a cutoff at 0.01, and 120 targets were enriched to 26 biological processes; the top 10 are shown in **Supplementary Table S3** and **Figure 4A**. The pathways were ranked by their nominal *p* values with a cutoff at 0.05, and the top 10 pathways included interleukin-4 and 13 signaling, phase I functionalization of compounds, drug induction of bile acid pathway, xenobiotics, tamoxifen metabolism, reversible hydration of carbon dioxide, electron transport chain, adrenoceptors, adenosine P1 receptors, and angiotensin-converting enzyme inhibitor pathway (**Figure 4B**). The functional targets involved in each pathway are illustrated in **Supplementary Table S4**. The C–T pathway/biological process network (C–T–P) was constructed using Cytoscape (**Figure 5**) based on the interactions among the 29 components, 120 consensus targets, and top 10 biological processes and pathways.

### Experimental Validation of Key Targets and Pathway

**Figure 6:**
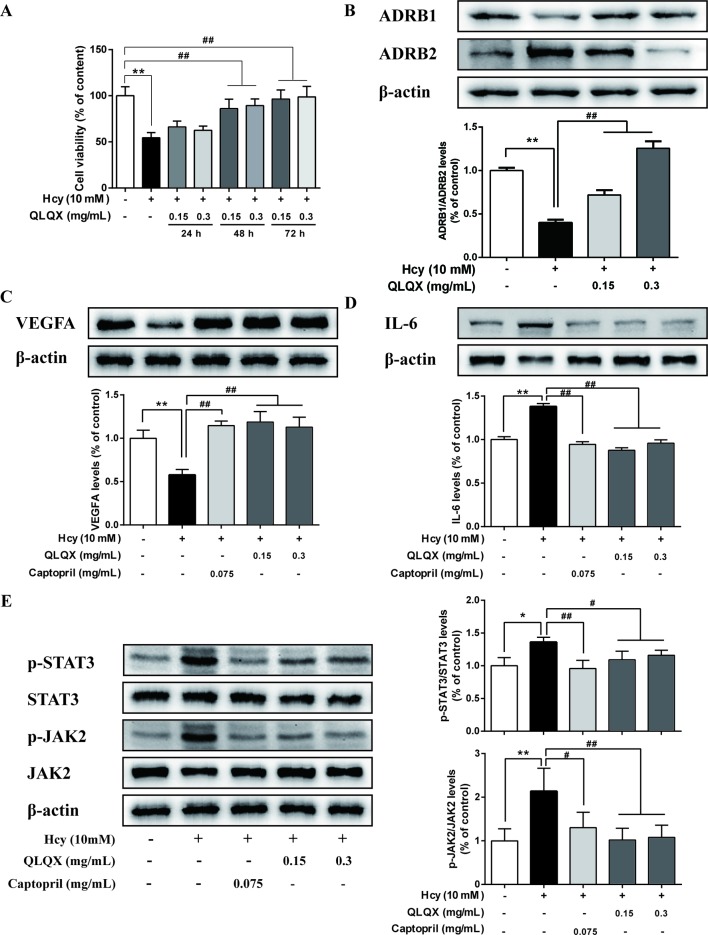
Experimental validation of key targets and pathway in HCMEC. **(A)** Effect of QLQX treatment on Hcy-induced HCMEC cell viability. HCMECs were treated with 0.15 mg/mL and 0.3 mg/mL QLQX for 24, 48, and 72 h, after then cells were treated with Hcy for 24 h. The cell viability was determined using the MTT assay. **(B)** Effect of QLQX treatment on Hcy-induced *ADRB1/Anti-ADRB2* ratio in HCMEC. **(C)** Effect of QLQX and captopril treatment on Hcy-induced *VEGFA* expression in HCMEC. **(D)** Effect of QLQX and captopril treatment on Hcy-induced *IL-6* expression in HCMEC. **(E)** Effect of QLQX and captopril treatment on Hcy-induced phosphorylation of STAT3/JAK2 signaling pathway. HCMECs were treated with 0.075 mg/mL, 0.3 mg/mL QLQX for 48 h and exposed to 10 mM Hcy for a further 24 h. The levels of *VEGFA, IL-6, p-STAT3*, and *p-JAK2* expression were determined by western blot. Data were presented as mean SD of three independent experiments. *p < 0.05 or **p < 0.01 compared with the Hcy-untreated control. ^#^ *p* < 0.05 or ^##^ *p* < 0.01 compared with the Hcy-treated control.

MTT assays showed that 0.15 (-L) and 0.3 mg/ml (-H) QLQX dramatically inhibited Hcy-induced injury in the HCMEC at 48 and 72 h in a time-dependent manner (**Figure 6**). To determine the mechanisms of QLQX in the treatment of CHF, some of the key proteins with a higher degree in the PPI network were experimentally validated in the HCMEC. Besides, considering that *ADRB1* and *ADRB2* play an essential role in heart failure (Doughty and Sharpe, 1997; Spadari et al., 2018), *ADRB1* and *ADRB2* were used as a control to validate the effect of QLQX. As shown in **Figure 6**, QLQX significantly increased the ratio between *ADRB1* and *ADRB2*. Meanwhile, QLQX significantly increased the expression level of *VEGFA* and inhibited the expression levels of *p-STAT3*, *p-JAK2*, and *IL-6* in a dose-dependent manner.

## Discussion

As a multicomponent drug, QLQX has been used for several years to treat CHF in China and has been demonstrated as effective in lowering the NT-proBNP level in CHF patients (Li et al., 2013). However, its “multicompound, multitarget” characteristics make it difficult to decipher the active ingredients and mechanisms of QLQX in the treatment against CHF.

Integrated strategies based on network pharmacology provide a useful approach to investigate the active components and molecular mechanisms. For instance, Gao et al. (2018) proposed a “main active compound-based network pharmacology” based on quantitative analysis of components to explore the anticancer mechanism of CKI. They selected herbs used in the clinical therapy of hepatoma to ascertain molecular targets and antitumor mechanisms, emphasizing the combination of clinical study and network pharmacology. However, in previous studies, all herbal ingredients were collected from herb databases and filtered according to absorption, distribution, metabolism, and excretion properties or drug-likeness value, which may be inconsistent with the ingredients present in the blood (Gao et al., 2016). Here, we proposed an integrated strategy *via* pharmacokinetics study, network analysis, and experimental validation to achieve an accurate and systematic exploration of the mechanisms of QLQX against CHF.

In this study, the 29 components of QLQX showing proper pharmacokinetics behavior in rats were determined to be the active ingredients of QLQX. These compounds were mainly categorized as triterpenoid saponins, phenolic acids, flavonoids, and alkaloids. Particularly, astragaloside, ginsenoside Rb_1_, Rb_2_, Rg_1_, Rg_3_, Rc, Rd, Re, Rf, and ginsenoside F_2_, as triterpenoid saponins, are known for their anti-inflammatory property during the period of ventricular remodeling (Sun Y. et al., 2016; Qi et al., 2017); salvianolic acid A, salvianolic acid B, danshensu, lithospermic acid, rosmarinic acid, and protocatechuic acid, as phenolic acids, exert cardioprotection through promoting angiogenesis in animal models (Yu et al., 2017); calycosin-7-glucoside, hydroxysafflor yellow A, formononetin, hesperidin, rutin, and quercetin, as flavonoids, can reduce cardiomyocytes damage and apoptosis and improve cardiac function by decreasing oxidative stress (Mattera et al., 2017); and aconitine, mesaconitine, hypaconitine, benzoylaconine, benzoylmesaconine, benzoylhypaconine, and sinapine bisulfate, as alkaloids, can improve left ventricular systolic and diastolic function (Liu et al., 2012). Moreover, the hub targets of these active ingredients were determined *via* combining network topological parameters with clustering and PPI network analysis. In total, 6 targets, namely, *VEGFA*, *CYP1A1*, *CYP2B6*, *ATP1A1*, *STAT3*, and *STAT4*, and 10 predicted functional targets, namely, *KDR*, *FLT1*, *NRP2*, *JAK2*, *EGFR*, *IL-6*, *AHR*, *ATP1B1*, *JAK1*, and *HIF1A* were collected as hub targets of QLQX against CHF.

As the leading cause of cardiovascular mortality, CHF is associated with many pathogenic factors, such as increased hemodynamic overload, ventricular remodeling, neurohormonal activation, and energy metabolism disorder (Gedela et al., 2015; Tanai and Frantz, 2015). In recent years, increasing evidence has shown that cardiac microvascular and microcirculation functions are closely related, in that, proper cardiac function requires myocardial oxygen balance. Furthermore, the perturbations in microcirculation caused by the interplay of neurohumoral, metabolic, and endothelium-derived factors lead to cardiac microvascular dysfunction and further result in cardiac insufficiency or heart failure (Den Uil et al., 2008; Heinonen et al., 2015).

It is thought that the presence of endothelial dysfunction, which induced by impairment of endothelium-dependent relaxation of blood vessels, might contributed to the pathogenesis of heart failure (Kishimoto et al., 2017). Previous study have suggested that Hcy could initiate mitochondrial dysfunction, which contributes to the cell apoptosis and chronic inflammation, thereby resulting in endothelial dysfunction (Han et al., 2015; Zhang et al., 2017). In this manuscript, the Hcy-induced HCMECs injury was used as the model to explore the role of QLQX in endothelial dysfunction of damaged microvascular endothelial cell. It has been shown that, captopril, as a positive control drug to treat HCMEC in our study, improves endothelium-dependent vasodilatation in patients with CHF (Drexler et al., 1995). Meanwhile, captopril can increase vascular endothelial growth factor (VEGFA) expression during the period of pathological angiogenesis and rarefaction and inhibit proinflammatory cytokine expression (IL-1β, IL-6, and IL-8) in cultured human coronary artery endothelial cells (Greene and Amaral, 2002; Haas et al., 2019). Studies have also reported that captopril exerted a cardioprotective effect on heart failure by inhibiting phosphorylation of JAK2/STAT3 (Zhang Y. et al., 2019).

We have shown that QLQX dramatically inhibited Hcy-induced injury on the HCMEC in a time-dependent manner as observed through MTT assays. Western blotting confirmed that QLQX significantly increased the ratio between *ADRB1* and *ADRB2*, upregulated the expression level of *VEGFA*, and downregulated the expression levels of *p-STAT3*, *p-JAK2*, and *IL-6* in Hcy-induced HCMEC. Evidence has shown that a reduction in the *ADRB1/ADRB2* ratio has been observed in heart failure, and the use of adrenoceptor beta blockers is a cornerstone of current heart failure therapy (Baker, 2014; Woo et al., 2015). Consistent with prior study, we validated the effect of QLQX on the expression of adrenoceptor beta, which showed the prediction power in network pharmacological analysis of our study. On this basis, CHF is defined by cardiac dysfunction associated with ventricular remodeling, and, more recently, an imbalance between angiogenesis and cardiac hypertrophy has increasingly been acknowledged as an additional contributing mechanism (Taimeh et al., 2013). Accordingly, the therapeutic effect of myocardial angiogenesis is emerging as a promising approach for the prevention and treatment of CHF (Vila et al., 2008; Oka et al., 2014). VEGFA, a cornerstone cytokine of angiogenesis, participates in the process of vascular remodeling and myocardial angiogenesis in CHF through maintenance and repair of luminal endothelium (Morine et al., 2016). Besides, increased expression of VEGFA levels has been associated with the process of angiogenesis, and it may be adopted as an indicator of revascularization (Kucukardali et al., 2008; Wang et al., 2017; Yan et al., 2019).

As shown in the present study, QLQX exerts a protective effect against IL-6 secretion induced by Hcy in the HCMEC, which may be associated with negative regulation of the Janus kinase signal transducer and signal transduction activator of transcription (JAK-STAT) signaling. Recent evidence has revealed that inflammation is a critical pathological process of CHF (Dick and Epelman, 2016; Ayoub et al., 2017; Cocco et al., 2017) and high levels of IL-6, a proinflammatory cytokine, have been reported to be an important mediator in chronic inflammatory and cardiovascular disorders (Smart et al., 2006; Bacchiega et al., 2017). Moreover, IL-6 exerts its action through a specific IL-6R and a soluble IL-6 receptor, whereby the IL-6/IL-6R complex binds to the membrane glycoprotein 130 to induce intracellular signaling pathways (Hirota et al., 2004; Ptaszynska-Kopczynska et al., 2017). The JAK-STAT pathway is a characteristic signal transduction pathway that plays a crucial role in this process (Mohri et al., 2012). Specifically, the activated JAK2, a key member of the Janus family of kinases, leads to phosphorylation and activation of a group of transcription factors collectively called STATs. Among them, STAT3 also appears to be involved in a broad range of cytoprotection activities, such as inflammation, angiogenesis, extracellular matrix composition, and apoptosis in the heart (Shen-Orr et al., 2016). Previous study has implicated astragaloside could improve vascular endothelial dysfunction induced by hyperglycemia by increasing eNOS expression and decreasing the content of IL-6 (Leng et al., 2018). Evidence provided by Jiang et al. (2015) showed calycosin protected vascular endothelial from LPS-induced endothelial injury through suppression of ROS and VEGFA level. Furthermore, it has been reported that salvianolic acid A inhibited endothelial dysfunction and vascular remodeling in spontaneously hypertensive rats (Teng et al., 2016). All these mentioned above suggest that some compounds in QLQX exerts a protective effect on vascular endothelial, which in another way could support the results in our study.

Therefore, we focused on the inflammation in the HCMEC, as we were interested in the role of the JAK/STAT signaling in the pathogenesis of QLQX against CHF. Inflammatory processes and angiogenesis may be interrelated during the process of CHF, and the angiogenesis factor promotes angiogenesis mainly through signaling pathways, of which JAK2-STAT3 is one of the important ones (Fujio et al., 2011). It was reported that inflammation and neovascularization in atheromatous plaques might be mediated by VEGFA (Moulton et al., 2003), and the antihuman IL-6 receptor monoclonal antibody was shown to improve endothelial function in patients with acute coronary syndromes (Holte et al., 2017). Thus, QLQX can rectify the injury of microvascular endothelial cells induced by Hcy while significantly decreasing the levels of *IL-6*, *p-JAK2*, and *p-STAT3*, which suggests that QLQX may inhibit inflammatory processes and promote angiogenesis in CHF *via* the JAK/STAT signaling pathway.

## Conclusion

In the current study, an integrated strategy was used to illustrate the active ingredients and molecular mechanisms of QLQX in the treatment of CHF by adopting pharmacokinetics study, network pharmacological analysis, and experimental validation. In total, 29 ingredients determined by pharmacokinetics study, instead of herb databases, were used for network pharmacology analysis. Through experimental validation of the hub targets (*VEGFA*, *IL-6*, *p-STAT3*, and *p-JAK2*), the JAK/STAT signaling pathway was identified as the mechanism of QLQX against inflammatory process in CHF. Additionally, the established C–T–P network remained a characteristic of multilink and multilevel comprehensive effects of QLQX. These results provided an efficient way to understand the pharmacological mechanisms of traditional Chinese medicine prescriptions.

## Data Availability

The data used to support the findings of this study are available from the corresponding author upon request.

## Ethics Statement

The animal study was reviewed and approved by the Ethics Committee of Tianjin University of Traditional Chinese Medicine (Tianjin, China). This study was carried out in accordance with the principles of the Basel Declaration and recommendations of guidelines of the National Institutes of Health.

## Author Contributions

YZ is the first author and performed all the experiments and drafted the manuscript. MZ revised the manuscript. FZ and SZ helped the first author and prepared the materials of this paper. WD and XX contributed toward study design, experimental setup, results supervision, and manuscript correction.

## Funding

This work was supported by the National Natural Science Foundation of China (81774227), Natural Science Fund of Tianjin City (17JCZDJC34600), Project of TCM and combining TCM and western medicine of Tianjin Health and Family Planning Committee (2015050), and Technology program in key areas of Tianjin (20190101).

## Conflict of Interest Statement

The authors declare that the research was conducted in the absence of any commercial or financial relationships that could be construed as a potential conflict of interest.

## Footnotes

1 https://www.drugbank.ca/

2 http://www.swisstargetprediction.ch/

3 http://sea.bkslab.org/

4 http://www.disgenet.org/web/DisGeNET/menu/home

5 https://string-db.org/
