# Supplementary material for: Integrating Pharmacokinetics Study, Network Analysis, and Experimental Validation to Uncover the Mechanism of Qiliqiangxin Capsule Against Chronic Heart Failure
Source: Front Pharmacol. 2019 Sep 18;10:1046. doi: 10.3389/fphar.2019.01046 (PMC6759796; doi:10.3389/fphar.2019.01046)
Supplement: Supplementary file 1 [file Table_1.doc]

**SUPPLEMENTARY TABLE S1 ׀** Putative targets of 29 components in QLQX.

| **No.** | **Components** | **Gene** | **Protein name** | **Uniprot ID** |
| --- | --- | --- | --- | --- |
| 1 | QLQX-1 | HPSE | Heparanase | Q9Y251 |
| 2 | QLQX-1 | UQCR10 | Cytochrome b-c1 complex subunit 9 | [Q9UDW1](http://www.uniprot.org/uniprot/Q9UDW1) |
| 3 | QLQX-1 | GLTP | Glycolipid transfer protein | [Q9NZD2](http://www.uniprot.org/uniprot/Q9NZD2) |
| 4 | QLQX-1 | SLCO1B3 | Solute carrier organic anion transporter family member 1B3 | Q9NPD5 |
| 5 | QLQX-1 | SDHC | Succinate dehydrogenase cytochrome b560 subunit, mitochondrial | Q99643 |
| 6 | QLQX-1 | RABGGTA | Geranylgeranyl transferase type-2 subunit alpha | [Q92696](http://www.uniprot.org/uniprot/Q92696) |
| 7 | QLQX-1 | HPSE2 | Heparanase | Q8WWQ2 |
| 8 | QLQX-1 | GLT6D1 | Putative glycosyltransferase 6 domain-containing protein 1 | Q7Z4J2 |
| 9 | QLQX-1 | CLPP | ATP-dependent Clp protease proteolytic subunit, mitochondrial | [Q16740](http://www.uniprot.org/uniprot/Q16740) |
| 10 | QLQX-1 | ETFDH | Electron transfer flavoprotein-ubiquinone oxidoreductase, mitochondrial | Q16134 |
| 11 | QLQX-1 | PPARD | Peroxisome proliferator-activated receptor delta | Q03181 |
| 12 | QLQX-1 | COX6A2 | Cytochrome c oxidase subunit 6A2, mitochondrial | Q02221 |
| 13 | QLQX-1 | FKBP1A | Peptidyl-prolyl cis-trans isomerase FKBP1A | P62942 |
| 14 | QLQX-1 | B2M | Beta-2-microglobulin | [P61769](http://www.uniprot.org/uniprot/P61769) |
| 15 | QLQX-1 | RABGGTB | Geranylgeranyl transferase type-2 subunit beta | P53611 |
| 16 | QLQX-1 | KCNJ3 | G protein-activated inward rectifier potassium channel 1 | P48549 |
| 17 | QLQX-1 | UQCRFS1 | Cytochrome b-c1 complex subunit Rieske, mitochondrial | P47985 |
| 18 | QLQX-1 | ADRA1B | Alpha-1B adrenergic receptor | P35368 |
| 19 | QLQX-1 | ADRA1A | Alpha-1A adrenergic receptor | P35348 |
| 20 | QLQX-1 | UQCRC1 | Cytochrome b-c1 complex subunit 1, mitochondrial | P31930 |
| 21 | QLQX-1 | SDHA | Succinate dehydrogenase [ubiquinone] flavoprotein subunit, mitochondrial | [P31040](http://www.uniprot.org/uniprot/P31040) |
| 22 | QLQX-1 | MIP | Lens fiber major intrinsic protein | [P30301](http://www.uniprot.org/uniprot/P30301) |
| 23 | QLQX-1 | AQP1 | Aquaporin-1 | P29972 |
| 24 | QLQX-1 | S100G | Protein S100-G | [P29377](http://www.uniprot.org/uniprot/P29377) |
| 25 | QLQX-1 | ADRA1D | Alpha-1D adrenergic receptor | P25100 |
| 26 | QLQX-1 | COX7B | Cytochrome c oxidase subunit 7B, mitochondrial | [P24311](http://www.uniprot.org/uniprot/P24311) |
| 27 | QLQX-1 | COX7A1 | Cytochrome c oxidase subunit 7A1, mitochondrial | [P24310](http://www.uniprot.org/uniprot/P24310) |
| 28 | QLQX-1 | UQCRC2 | Cytochrome b-c1 complex subunit 2, mitochondrial | P22695 |
| 29 | QLQX-1 | SDHB | Succinate dehydrogenase [ubiquinone] iron-sulfur subunit, mitochondrial | P21912 |
| 30 | QLQX-1 | COX5A | Cytochrome c oxidase subunit 5A, mitochondrial | P20674 |
| 31 | QLQX-1 | CHRM3 | Muscarinic acetylcholine receptor M3 | P20309 |
| 32 | QLQX-1 | AMY2B | Alpha-amylase 2B | P19961 |
| 33 | QLQX-1 | LGALS3 | Galectin-3 | P17931 |
| 34 | QLQX-1 | ABO | Histo-blood group ABO system transferase | P16442 |
| 35 | QLQX-1 | PNLIP | Pancreatic triacylglycerol lipase | [P16233](http://www.uniprot.org/uniprot/P16233) |
| 36 | QLQX-1 | COX7C | Cytochrome c oxidase subunit 7C, mitochondrial | P15954 |
| 37 | QLQX-1 | VEGFA | Vascular endothelial growth factor A | P15692 |
| 38 | QLQX-1 | UQCRB | Cytochrome b-c1 complex subunit 7 | P14927 |
| 39 | QLQX-1 | COX6B1 | Cytochrome c oxidase subunit 6B1 | P14854 |
| 40 | QLQX-1 | RARG | Retinoic acid receptor gamma | P13631 |
| 41 | QLQX-1 | COX4I1 | Cytochrome c oxidase subunit 4 isoform 1, mitochondrial | P13073 |
| 42 | QLQX-1 | CDK4 | Cyclin-dependent kinase 4 | P11802 |
| 43 | QLQX-1 | CHRM1 | Muscarinic acetylcholine receptor M1 | P11229 |
| 44 | QLQX-1 | MBL2 | Mannose-binding protein C | [P11226](http://www.uniprot.org/uniprot/P11226) |
| 45 | QLQX-1 | COX5B | Cytochrome c oxidase subunit 5B, mitochondrial | P10606 |
| 46 | QLQX-1 | COX8A | Cytochrome c oxidase subunit 8A, mitochondrial | P10176 |
| 47 | QLQX-1 | UBC | Polyubiquitin-C | P0CG48 |
| 48 | QLQX-1 | COX6C | Cytochrome c oxidase subunit 6C | P09669 |
| 49 | QLQX-1 | FGF2 | Fibroblast growth factor 2 | P09038 |
| 50 | QLQX-1 | CHRM5 | Muscarinic acetylcholine receptor M5 | P08912 |
| 51 | QLQX-1 | CYC1 | Cytochrome c1, heme protein, mitochondrial | P08574 |
| 52 | QLQX-1 | CHRM4 | Muscarinic acetylcholine receptor M4 | P08173 |
| 53 | QLQX-1 | CHRM2 | Muscarinic acetylcholine receptor M2 | P08172 |
| 54 | QLQX-1 | RHO | Rhodopsin | P08100 |
| 55 | QLQX-1 | RNASE1 | Ribonuclease pancreatic | P07998 |
| 56 | QLQX-1 | UQCRH | Cytochrome b-c1 complex subunit 6, mitochondrial | P07919 |
| 57 | QLQX-1 | CDK1 | Cyclin-dependent kinase 1 | P06493 |
| 58 | QLQX-1 | FGF1 | Fibroblast growth factor 1 | P05230 |
| 59 | QLQX-1 | S100B | Protein S100-B | [P04271](http://www.uniprot.org/uniprot/P04271) |
| 60 | QLQX-1 | CLPS | Colipase | P04118 |
| 61 | QLQX-1 | HLA-B | HLA class I histocompatibility antigen, B-27 alpha chain | P03989 |
| 62 | QLQX-1 | MT-ND1 | NADH-ubiquinone oxidoreductase chain 1 | P03886 |
| 63 | QLQX-1 | SERPINA1 | Alpha-1-antitrypsin | [P01009](http://www.uniprot.org/uniprot/P01009) |
| 64 | QLQX-1 | SERPINC1 | Antithrombin-III | P01008 |
| 65 | QLQX-1 | MT-CO3 | Cytochrome c oxidase subunit 3 | P00414 |
| 66 | QLQX-1 | MT-CO2 | Cytochrome c oxidase subunit 2 | P00403 |
| 67 | QLQX-1 | MT-CO1 | Cytochrome c oxidase subunit 1 | P00395 |
| 68 | QLQX-1 | MT-CYB | Cytochrome b | P00156 |
| 69 | QLQX-1 | UQCRQ | Cytochrome b-c1 complex subunit 8 | O14949 |
| 70 | QLQX-1 | SDHD | Succinate dehydrogenase [ubiquinone] cytochrome b small subunit, mitochondrial | O14521 |
| 71 | QLQX-10 | SLCO1B1 | Solute carrier organic anion transporter family member 1B1 | [Q9Y6L6](http://www.uniprot.org/uniprot/Q9Y6L6) |
| 72 | QLQX-10 | HPSE | Heparanase | Q9Y251 |
| 73 | QLQX-10 | SLCO1C1 | Solute carrier organic anion transporter family member 1C1 | Q9NYB5 |
| 74 | QLQX-10 | SLCO1B3 | Solute carrier organic anion transporter family member 1B3 | Q9NPD5 |
| 75 | QLQX-10 | HPSE2 | Inactive heparanase-2 | Q8WWQ2 |
| 76 | QLQX-10 | SLC22A8 | Solute carrier family 22 member 8 | [Q8TCC7](http://www.uniprot.org/uniprot/Q8TCC7) |
| 77 | QLQX-10 | SLCO4C1 | Solute carrier organic anion transporter family member 4C1 | Q6ZQN7 |
| 78 | QLQX-10 | STAT4 | Signal transducer and activator of transcription 4 | Q14765 |
| 79 | QLQX-10 | STAT2 | Signal transducer and activator of transcription 2 | P52630 |
| 80 | QLQX-10 | ATP1A2 | Sodium/potassium-transporting ATPase subunit alpha-2 | P50993 |
| 81 | QLQX-10 | SLCO1A2 | Solute carrier organic anion transporter family member 1A2 | P46721 |
| 82 | QLQX-10 | STAT1 | Signal transducer and activator of transcription 1-alpha/beta | P42224 |
| 83 | QLQX-10 | STAT3 | Signal transducer and activator of transcription 3 | P40763 |
| 84 | QLQX-10 | CYP2B6 | Cytochrome P450 2B6 | [P20813](http://www.uniprot.org/uniprot/P20813) |
| 85 | QLQX-10 | CHRM3 | Muscarinic acetylcholine receptor M3 | P20309 |
| 86 | QLQX-10 | FLT1 | Vascular endothelial growth factor receptor 1 | P17948 |
| 87 | QLQX-10 | VEGFA | Vascular endothelial growth factor A | P15692 |
| 88 | QLQX-10 | ATP1A3 | Sodium/potassium-transporting ATPase subunit alpha-3 | P13637 |
| 89 | QLQX-10 | CHRM1 | Muscarinic acetylcholine receptor M1 | P11229 |
| 90 | QLQX-10 | FGF2 | Fibroblast growth factor 2 | P09038 |
| 91 | QLQX-10 | CHRM5 | Muscarinic acetylcholine receptor M5 | P08912 |
| 92 | QLQX-10 | CHRM4 | Muscarinic acetylcholine receptor M4 | P08173 |
| 93 | QLQX-10 | CHRM2 | Muscarinic acetylcholine receptor M2 | P08172 |
| 94 | QLQX-10 | FGF1 | Fibroblast growth factor 1 | P05230 |
| 95 | QLQX-10 | ATP1A1 | Sodium/potassium-transporting ATPase subunit alpha-1 | P05023 |
| 96 | QLQX-10 | CYP1A1 | Cytochrome P450 1A1 | P04798 |
| 97 | QLQX-10 | AMY2A | Pancreatic alpha-amylase | P04746 |
| 98 | QLQX-11 | SLCO1B1 | Solute carrier organic anion transporter family member 1B1 | [Q9Y6L6](http://www.uniprot.org/uniprot/Q9Y6L6) |
| 99 | QLQX-11 | HPSE | Heparanase | Q9Y251 |
| 100 | QLQX-11 | SLCO1C1 | Solute carrier organic anion transporter family member 1C1 | Q9NYB5 |
| 101 | QLQX-11 | SLCO1B3 | Solute carrier organic anion transporter family member 1B3 | Q9NPD5 |
| 102 | QLQX-11 | HPSE2 | Inactive heparanase-2 | Q8WWQ2 |
| 103 | QLQX-11 | SLC22A8 | Solute carrier family 22 member 8 | [Q8TCC7](http://www.uniprot.org/uniprot/Q8TCC7) |
| 104 | QLQX-11 | SLCO4C1 | Solute carrier organic anion transporter family member 4C1 | Q6ZQN7 |
| 105 | QLQX-11 | STAT4 | Signal transducer and activator of transcription 4 | Q14765 |
| 106 | QLQX-11 | CDK6 | Cyclin-dependent kinase 6 | Q00534 |
| 107 | QLQX-11 | CDK3 | Cyclin-dependent kinase 3 | Q00526 |
| 108 | QLQX-11 | STAT2 | Signal transducer and activator of transcription 2 | P52630 |
| 109 | QLQX-11 | ATP1A2 | Sodium/potassium-transporting ATPase subunit alpha-2 | P50993 |
| 110 | QLQX-11 | SLCO1A2 | Solute carrier organic anion transporter family member 1A2 | P46721 |
| 111 | QLQX-11 | STAT1 | Signal transducer and activator of transcription 1-alpha/beta | P42224 |
| 112 | QLQX-11 | STAT3 | Signal transducer and activator of transcription 3 | P40763 |
| 113 | QLQX-11 | CDK2 | Cyclin-dependent kinase 2 | P24941 |
| 114 | QLQX-11 | CYP2B6 | Cytochrome P450 2B6 | [P20813](http://www.uniprot.org/uniprot/P20813) |
| 115 | QLQX-11 | VEGFA | Vascular endothelial growth factor A | P15692 |
| 116 | QLQX-11 | ATP1A3 | Sodium/potassium-transporting ATPase subunit alpha-3 | P13637 |
| 117 | QLQX-11 | CDK4 | Cyclin-dependent kinase 4 | P11802 |
| 118 | QLQX-11 | FGF2 | Fibroblast growth factor 2 | P09038 |
| 119 | QLQX-11 | CHRM5 | Muscarinic acetylcholine receptor M5 | P08912 |
| 120 | QLQX-11 | CDK1 | Cyclin-dependent kinase 1 | P06493 |
| 121 | QLQX-11 | FGF1 | Fibroblast growth factor 1 | P05230 |
| 122 | QLQX-11 | ATP1A1 | Sodium/potassium-transporting ATPase subunit alpha-1 | P05023 |
| 123 | QLQX-11 | CYP1A1 | Cytochrome P450 1A1 | P04798 |
| 124 | QLQX-12 | SLCO1B1 | Solute carrier organic anion transporter family member 1B1 | [Q9Y6L6](http://www.uniprot.org/uniprot/Q9Y6L6) |
| 125 | QLQX-12 | HPSE | Heparanase | Q9Y251 |
| 126 | QLQX-12 | SLCO1C1 | Solute carrier organic anion transporter family member 1C1 | Q9NYB5 |
| 127 | QLQX-12 | SLCO1B3 | Solute carrier organic anion transporter family member 1B3 | Q9NPD5 |
| 128 | QLQX-12 | HPSE2 | Inactive heparanase-2 | Q8WWQ2 |
| 129 | QLQX-12 | SLC22A8 | Solute carrier family 22 member 8 | [Q8TCC7](http://www.uniprot.org/uniprot/Q8TCC7) |
| 130 | QLQX-12 | SLCO4C1 | Solute carrier organic anion transporter family member 4C1 | Q6ZQN7 |
| 131 | QLQX-12 | STAT4 | Signal transducer and activator of transcription 4 | Q14765 |
| 132 | QLQX-12 | STAT2 | Signal transducer and activator of transcription 2 | P52630 |
| 133 | QLQX-12 | ATP1A2 | Sodium/potassium-transporting ATPase subunit alpha-2 | P50993 |
| 134 | QLQX-12 | SLCO1A2 | Solute carrier organic anion transporter family member 1A2 | P46721 |
| 135 | QLQX-12 | STAT1 | Signal transducer and activator of transcription 1-alpha/beta | P42224 |
| 136 | QLQX-12 | STAT3 | Signal transducer and activator of transcription 3 | P40763 |
| 137 | QLQX-12 | PTAFR | Platelet-activating factor receptor | P25105 |
| 138 | QLQX-12 | CYP2B6 | Cytochrome P450 2B6 | [P20813](http://www.uniprot.org/uniprot/P20813) |
| 139 | QLQX-12 | CHRM3 | Muscarinic acetylcholine receptor M3 | P20309 |
| 140 | QLQX-12 | VEGFA | Vascular endothelial growth factor A | P15692 |
| 141 | QLQX-12 | ATP1A3 | Sodium/potassium-transporting ATPase subunit alpha-3 | P13637 |
| 142 | QLQX-12 | CHRM1 | Muscarinic acetylcholine receptor M1 | P11229 |
| 143 | QLQX-12 | FGF2 | Fibroblast growth factor 2 | P09038 |
| 144 | QLQX-12 | CHRM5 | Muscarinic acetylcholine receptor M5 | P08912 |
| 145 | QLQX-12 | CHRM4 | Muscarinic acetylcholine receptor M4 | P08173 |
| 146 | QLQX-12 | CHRM2 | Muscarinic acetylcholine receptor M2 | P08172 |
| 147 | QLQX-12 | FGF1 | Fibroblast growth factor 1 | P05230 |
| 148 | QLQX-12 | ATP1A1 | Sodium/potassium-transporting ATPase subunit alpha-1 | P05023 |
| 149 | QLQX-12 | CYP1A1 | Cytochrome P450 1A1 | P04798 |
| 150 | QLQX-13 | CA5B | Carbonic anhydrase 5B, mitochondrial | Q9Y2D0 |
| 151 | QLQX-13 | CA14 | Carbonic anhydrase 14 | Q9ULX7 |
| 152 | QLQX-13 | SLCO1C1 | Solute carrier organic anion transporter family member 1C1 | Q9NYB5 |
| 153 | QLQX-13 | TDP1 | Tyrosyl-DNA phosphodiesterase 1 | Q9NUW8 |
| 154 | QLQX-13 | AKR1E2 | 1,5-anhydro-D-fructose reductase | Q96JD6 |
| 155 | QLQX-13 | Ca13 | Carbonic anhydrase 13 | Q8N1Q1 |
| 156 | QLQX-13 | NFE2L2 | Nuclear factor erythroid 2-related factor 2 | Q16236 |
| 157 | QLQX-13 | GLO1 | Lactoylglutathione lyase | Q04760 |
| 158 | QLQX-13 | [CA7](http://zinc15.docking.org/genes/CA7) | Carbonic anhydrase 7 | P43166 |
| 159 | QLQX-13 | FRK | Tyrosine-protein kinase FRK | P42685 |
| 160 | QLQX-13 | MMP12 | Macrophage metalloelastase | P39900 |
| 161 | QLQX-13 | SNCA | Alpha-synuclein | P37840 |
| 162 | QLQX-13 | HSD17B3 | Testosterone 17-beta-dehydrogenase 3 | P37058 |
| 163 | QLQX-13 | CA5A | Carbonic anhydrase 5A, mitochondrial | P35218 |
| 164 | QLQX-13 | CA6 | Carbonic anhydrase 6 | P23280 |
| 165 | QLQX-13 | [CA4](http://zinc15.docking.org/genes/CA4) | Carbonic anhydrase 4 | P22748 |
| 166 | QLQX-13 | DDC | Aromatic-L-amino-acid decarboxylase | P20711 |
| 167 | QLQX-13 | AKR1C4 | Aldo-keto reductase family 1 member C4 | P17516 |
| 168 | QLQX-13 | AKR1B1 | Aldose reductase | P15121 |
| 169 | QLQX-13 | MMP9 | Matrix metalloproteinase-9 | P14780 |
| 170 | QLQX-13 | AKR1A1 | Alcohol dehydrogenase [NADP(+)] | P14550 |
| 171 | QLQX-13 | SRC | Proto-oncogene tyrosine-protein kinase Src | P12931 |
| 172 | QLQX-13 | ALOX5 | Arachidonate 5-lipoxygenase | P09917 |
| 173 | QLQX-13 | FGR | Tyrosine-protein kinase Fgr | P09769 |
| 174 | QLQX-13 | MMP3 | Stromelysin-1 | P08254 |
| 175 | QLQX-13 | MMP2 | 72 kDa type IV collagenase | P08253 |
| 176 | QLQX-13 | YES1 | Tyrosine-protein kinase Yes | P07947 |
| 177 | QLQX-13 | [TH](http://zinc15.docking.org/genes/TH) | Tyrosine 3-monooxygenase | P07101 |
| 178 | QLQX-13 | FYN | Tyrosine-protein kinase Fyn | P06241 |
| 179 | QLQX-13 | APP | Amyloid-beta A4 protein | P05067 |
| 180 | QLQX-13 | MMP1 | Interstitial collagenase | P03956 |
| 181 | QLQX-13 | TTR | Transthyretin | P02766 |
| 182 | QLQX-13 | [CA2](http://zinc15.docking.org/genes/CA2) | Carbonic anhydrase 2 | P00918 |
| 183 | QLQX-13 | KCNK2 | Potassium channel subfamily K member 2 | O95069 |
| 184 | QLQX-13 | AKR1B10 | Aldo-keto reductase family 1 member B10 | O60218 |
| 185 | QLQX-13 | [CA12](http://zinc15.docking.org/genes/CA12) | Carbonic anhydrase 12 | O43570 |
| 186 | QLQX-13 | AlOX15B | Arachidonate 15-lipoxygenase B | O15296 |
| 187 | QLQX-13 | AKR1B15 | Aldo-keto reductase family 1 member B15 | C9JRZ8 |
| 188 | QLQX-14 | MMP27 | Matrix metalloproteinase-27 | Q9H306 |
| 189 | QLQX-14 | AKR1E2 | 1,5-anhydro-D-fructose reductase | Q96JD6 |
| 190 | QLQX-14 | MMP13 | Collagenase 3 | P45452 |
| 191 | QLQX-14 | MMP12 | Macrophage metalloelastase | P39900 |
| 192 | QLQX-14 | PTGS1 | Prostaglandin G/H synthase 1 | P23219 |
| 193 | QLQX-14 | MMP8 | Neutrophil collagenase | P22894 |
| 194 | QLQX-14 | CA4 | Carbonic anhydrase 4 | P22748 |
| 195 | QLQX-14 | AKR1C4 | Aldo-keto reductase family 1 member C4 | P17516 |
| 196 | QLQX-14 | AKR1B1 | Aldose reductase | P15121 |
| 197 | QLQX-14 | MMP9 | Matrix metalloproteinase-9 | P14780 |
| 198 | QLQX-14 | AKR1A1 | Alcohol dehydrogenase [NADP(+)] | P14550 |
| 199 | QLQX-14 | MMP10 | Stromelysin-2 | P09238 |
| 200 | QLQX-14 | MMP3 | Stromelysin-1 | P08254 |
| 201 | QLQX-14 | MMP2 | 72 kDa type IV collagenase | P08253 |
| 202 | QLQX-14 | MMP1 | Interstitial collagenase | P03956 |
| 203 | QLQX-14 | TTR | Transthyretin | P02766 |
| 204 | QLQX-14 | AKR1B10 | Aldo-keto reductase family 1 member B10 | O60218 |
| 205 | QLQX-14 | AKR1B15 | Aldo-keto reductase family 1 member B15 | C9JRZ8 |
| 206 | QLQX-15 | PLAA | Phospholipase A-2-activating protein | Q9Y263 |
| 207 | QLQX-15 | RPL26L1 | 60S ribosomal protein L26-like 1 | Q9UNX3 |
| 208 | QLQX-15 | CA14 | Carbonic anhydrase 14 | Q9ULX7 |
| 209 | QLQX-15 | RPL24D1 | Probable ribosome biogenesis protein RLP24 | Q9UHA3 |
| 210 | QLQX-15 | SLCO1C1 | Solute carrier organic anion transporter family member 1C1 | Q9NYB5 |
| 211 | QLQX-15 | TDP1 | Tyrosyl-DNA phosphodiesterase 1 | Q9NUW8 |
| 212 | QLQX-15 | ACE2 | Angiotensin-converting enzyme 2 | Q9BYF1 |
| 213 | QLQX-15 | RPL10L | 60S ribosomal protein L10-like | Q96L21 |
| 214 | QLQX-15 | CA13 | Carbonic anhydrase 13 | Q8N1Q1 |
| 215 | QLQX-15 | SLC22A6 | Solute carrier family 22 member 6 | Q4U2R8 |
| 216 | QLQX-15 | KYNU | Kynureninase | Q16719 |
| 217 | QLQX-15 | SLC15A2 | Solute carrier family 15 member 2 | Q16348 |
| 218 | QLQX-15 | FUT7 | Alpha-(1,3)-fucosyltransferase 7 | Q11130 |
| 219 | QLQX-15 | ENPEP | Glutamyl aminopeptidase | Q07075 |
| 220 | QLQX-15 | FOLH1 | Glutamate carboxypeptidase 2 | Q04609 |
| 221 | QLQX-15 | RPL109 | 60S ribosomal protein L19 | P84098 |
| 222 | QLQX-15 | RPL8 | 60S ribosomal protein L8 | P62917 |
| 223 | QLQX-15 | RPL11 | 60S ribosomal protein L11 | P62913 |
| 224 | QLQX-15 | RPL23 | 60S ribosomal protein L23 | P62829 |
| 225 | QLQX-15 | RPL23A | 60S ribosomal protein L23a | [P62750](http://www.uniprot.org/uniprot/P62750) |
| 226 | QLQX-15 | YWHAG | 14-3-3 protein gamma | P61981 |
| 227 | QLQX-15 | RPL37 | 60S ribosomal protein L37 | P61927 |
| 228 | QLQX-15 | RPL15 | 60S ribosomal protein L15 | P61313 |
| 229 | QLQX-15 | ARPP19 | cAMP-regulated phosphoprotein 19 | P56211 |
| 230 | QLQX-15 | SNU13 | NHP2-like protein 1 | P55769 |
| 231 | QLQX-15 | YARS | Tyrosine--tRNA ligase, cytoplasmic | P54577 |
| 232 | QLQX-15 | CACNA2D1 | Voltage-dependent calcium channel subunit alpha-2/delta-1 | P54289 |
| 233 | QLQX-15 | SLC15A1 | Solute carrier family 15 member 1 | P46059 |
| 234 | QLQX-15 | CA7 | Carbonic anhydrase 7 | P43166 |
| 235 | QLQX-15 | CSK | Tyrosine-protein kinase CSK | P41240 |
| 236 | QLQX-15 | RPL13A | 60S ribosomal protein L13a | P40429 |
| 237 | QLQX-15 | RPL3 | 60S ribosomal protein L3 | P39023 |
| 238 | QLQX-15 | SNCA | Alpha-synuclein | P37840 |
| 239 | QLQX-15 | PTGS2 | Prostaglandin G/H synthase 2 | P35354 |
| 240 | QLQX-15 | CA5A | Carbonic anhydrase 5A, mitochondrial | P35218 |
| 241 | QLQX-15 | ADORA2B | Adenosine receptor A2b | P29275 |
| 242 | QLQX-15 | [IGFBP5](http://zinc15.docking.org/genes/IGFBP5) | Insulin-like growth factor-binding protein 5 | P24593 |
| 243 | QLQX-15 | IGFBP6 | Insulin-like growth factor-binding protein 6 | P24592 |
| 244 | QLQX-15 | CA6 | Carbonic anhydrase 6 | P23280 |
| 245 | QLQX-15 | CA4 | Carbonic anhydrase 4 | P22748 |
| 246 | QLQX-15 | IGFBP4 | Insulin-like growth factor-binding protein 4 | P22692 |
| 247 | QLQX-15 | COMT | Catechol O-methyltransferase | P21964 |
| 248 | QLQX-15 | DDC | Aromatic-L-amino-acid decarboxylase | P20711 |
| 249 | QLQX-15 | IGFBP2 | Insulin-like growth factor-binding protein 2 | P18065 |
| 250 | QLQX-15 | ALOX12 | Arachidonate 12-lipoxygenase, 12S-type | P18054 |
| 251 | QLQX-15 | IGFBP3 | Insulin-like growth factor-binding protein 3 | P17936 |
| 252 | QLQX-15 | AKR1C4 | Aldo-keto reductase family 1 member C4 | P17516 |
| 253 | QLQX-15 | ALOX15 | Arachidonate 15-lipoxygenase | P16050 |
| 254 | QLQX-15 | EZR | Ezrin | P15311 |
| 255 | QLQX-15 | CPA3 | Mast cell carboxypeptidase A | P15088 |
| 256 | QLQX-15 | CPA1 | Carboxypeptidase A1 | [P15085](http://www.uniprot.org/uniprot/P15085) |
| 257 | QLQX-15 | DAO | D-amino-acid oxidase | P14920 |
| 258 | QLQX-15 | TYR | Tyrosinase | P14679 |
| 259 | QLQX-15 | ADRB3 | Beta-3 adrenergic receptor | P13945 |
| 260 | QLQX-15 | ACE | Angiotensin-converting enzyme | P12821 |
| 261 | QLQX-15 | PCNA | Proliferating cell nuclear antigen | P12004 |
| 262 | QLQX-15 | THRB | Thyroid hormone receptor beta | P10828 |
| 263 | QLQX-15 | THRA | Thyroid hormone receptor alpha | P10827 |
| 264 | QLQX-15 | ALPG | Alkaline phosphatase, germ cell type | P10696 |
| 265 | QLQX-15 | MAPT | Microtubule-associated protein tau | P10636 |
| 266 | QLQX-15 | DBH | Dopamine beta-hydroxylase | P09172 |
| 267 | QLQX-15 | IGFBP1 | Insulin-like growth factor-binding protein 1 | P08833 |
| 268 | QLQX-15 | ADRB1 | Beta-1 adrenergic receptor | P08588 |
| 269 | QLQX-15 | MME | Neprilysin | P08473 |
| 270 | QLQX-15 | MMP2 | 72 kDa type IV collagenase | P08253 |
| 271 | QLQX-15 | ADRB2 | Beta-2 adrenergic receptor | P07550 |
| 272 | QLQX-15 | CA3 | Carbonic anhydrase 3 | P07451 |
| 273 | QLQX-15 | [TH](http://zinc15.docking.org/genes/TH) | Tyrosine 3-monooxygenase | P07101 |
| 274 | QLQX-15 | FYN | Tyrosine-protein kinase Fyn | P06241 |
| 275 | QLQX-15 | LCK | Tyrosine-protein kinase Lck | P06239 |
| 276 | QLQX-15 | ALPL | Alkaline phosphatase, tissue-nonspecific isozyme | P05186 |
| 277 | QLQX-15 | ERBB2 | Receptor tyrosine-protein kinase erbB-2 | P04626 |
| 278 | QLQX-15 | ALB | Serum albumin | P02768 |
| 279 | QLQX-15 | TTR | Transthyretin | P02766 |
| 280 | QLQX-15 | HRAS | GTPase HRas | P01112 |
| 281 | QLQX-15 | CA2 | Carbonic anhydrase 2 | P00918 |
| 282 | QLQX-15 | CA1 | Carbonic anhydrase 1 | P00915 |
| 283 | QLQX-15 | EGFR | Epidermal growth factor receptor | P00533 |
| 284 | QLQX-15 | CA12 | Carbonic anhydrase 12 | O43570 |
| 285 | QLQX-15 | MLNR | Motilin receptor | O43193 |
| 286 | QLQX-15 | AlOX15B | Arachidonate 15-lipoxygenase B | O15296 |
| 287 | QLQX-15 | KMO | Kynurenine 3-monooxygenase | O15229 |
| 288 | QLQX-15 | KDM4E | Lysine-specific demethylase 4E | B2RXH2 |
| 289 | QLQX-15 | SLC22A20 | Solute carrier family 22 member 20 | A6NK97 |
| 290 | QLQX-16 | MMP27 | Matrix metalloproteinase-27 | Q9H306 |
| 291 | QLQX-16 | MMP13 | Collagenase 3 | P45452 |
| 292 | QLQX-16 | [CA7](http://zinc15.docking.org/genes/CA7) | Carbonic anhydrase 7 | P43166 |
| 293 | QLQX-16 | MMP12 | Macrophage metalloelastase | P39900 |
| 294 | QLQX-16 | PTGS2 | Prostaglandin G/H synthase 2 | P35354 |
| 295 | QLQX-16 | PTGS1 | Prostaglandin G/H synthase 1 | P23219 |
| 296 | QLQX-16 | MMP8 | Neutrophil collagenase | P22894 |
| 297 | QLQX-16 | [CA4](http://zinc15.docking.org/genes/CA4) | Carbonic anhydrase 4 | P22748 |
| 298 | QLQX-16 | AKR1C4 | Aldo-keto reductase family 1 member C4 | P17516 |
| 299 | QLQX-16 | AKR1B1 | Aldose reductase | P15121 |
| 300 | QLQX-16 | MMP9 | Matrix metalloproteinase-9 | P14780 |
| 301 | QLQX-16 | AKR1A1 | Alcohol dehydrogenase [NADP(+)] | P14550 |
| 302 | QLQX-16 | MMP10 | Stromelysin-2 | P09238 |
| 303 | QLQX-16 | MMP3 | Stromelysin-1 | P08254 |
| 304 | QLQX-16 | MMP2 | 72 kDa type IV collagenase | P08253 |
| 305 | QLQX-16 | MMP1 | Interstitial collagenase | P03956 |
| 306 | QLQX-16 | TTR | Transthyretin | P02766 |
| 307 | QLQX-16 | AKR1B10 | Aldo-keto reductase family 1 member B10 | O60218 |
| 308 | QLQX-16 | [CA12](http://zinc15.docking.org/genes/CA12) | Carbonic anhydrase 12 | O43570 |
| 309 | QLQX-16 | AKR1B15 | Aldo-keto reductase family 1 member B15 | C9JRZ8 |
| 310 | QLQX-17 | KDM4E | Lysine-specific demethylase 4E | B2RXH2 |
| 311 | QLQX-17 | IKBKG | NF-kappa-B essential modulator | Q9Y6K9 |
| 312 | QLQX-17 | CA5B | Carbonic anhydrase 5B, mitochondrial | Q9Y2D0 |
| 313 | QLQX-17 | ABCG2 | ATP-binding cassette sub-family G member 2 | Q9UNQ0 |
| 314 | QLQX-17 | CA14 | Carbonic anhydrase 14 | Q9ULX7 |
| 315 | QLQX-17 | SLCO1C1 | Solute carrier organic anion transporter family member 1C1 | Q9NYB5 |
| 316 | QLQX-17 | TDP1 | Tyrosyl-DNA phosphodiesterase 1 | Q9NUW8 |
| 317 | QLQX-17 | TUBB1 | Tubulin beta-2 chain | Q9H4B7 |
| 318 | QLQX-17 | NR1H4 | Bile acid receptor | Q96RI1 |
| 319 | QLQX-17 | AKR1E2 | 1,5-anhydro-D-fructose reductase | Q96JD6 |
| 320 | QLQX-17 | CA13 | Carbonic anhydrase 13 | Q8N1Q1 |
| 321 | QLQX-17 | NFE2L2 | Nuclear factor erythroid 2-related factor 2 | Q16236 |
| 322 | QLQX-17 | NR0B2 | Nuclear receptor subfamily 0 group B member 2 | Q15466 |
| 323 | QLQX-17 | EP300 | Histone acetyltransferase p300 | Q09472 |
| 324 | QLQX-17 | PPARA | Peroxisome proliferator-activated receptor alpha | Q07869 |
| 325 | QLQX-17 | MCL1 | Induced myeloid leukemia cell differentiation protein Mcl-1 | Q07820 |
| 326 | QLQX-17 | DNM1 | Dynamin-1 | Q05193 |
| 327 | QLQX-17 | GLO1 | Lactoylglutathione lyase | Q04760 |
| 328 | QLQX-17 | PPARD | Peroxisome proliferator-activated receptor delta | Q03181 |
| 329 | QLQX-17 | TUBA4A | Tubulin alpha-4A chain | P68366 |
| 330 | QLQX-17 | ARPP19 | cAMP-regulated phosphoprotein 19 | P56211 |
| 331 | QLQX-17 | YARS | Tyrosine--tRNA ligase, cytoplasmic | P54577 |
| 332 | QLQX-17 | CA7 | Carbonic anhydrase 7 | P43166 |
| 333 | QLQX-17 | FRK | Tyrosine-protein kinase FRK | P42685 |
| 334 | QLQX-17 | AKR1C3 | Aldo-keto reductase family 1 member C3 | P42330 |
| 335 | QLQX-17 | MMP12 | Macrophage metalloelastase | P39900 |
| 336 | QLQX-17 | SNCA | Alpha-synuclein | P37840 |
| 337 | QLQX-17 | PPARG | Peroxisome proliferator-activated receptor gamma | P37231 |
| 338 | QLQX-17 | HSD17B3 | Testosterone 17-beta-dehydrogenase 3 | P37058 |
| 339 | QLQX-17 | CA5A | Carbonic anhydrase 5A, mitochondrial | P35218 |
| 340 | QLQX-17 | [HSD11B1](http://zinc15.docking.org/genes/HSD11B1) | Corticosteroid 11-beta-dehydrogenase isozyme 1 | P28845 |
| 341 | QLQX-17 | IGFBP5 | Insulin-like growth factor-binding protein 5 | P24593 |
| 342 | QLQX-17 | CA6 | Carbonic anhydrase 6 | P23280 |
| 343 | QLQX-17 | CA4 | Carbonic anhydrase 4 | P22748 |
| 344 | QLQX-17 | DDC | Aromatic-L-amino-acid decarboxylase | P20711 |
| 345 | QLQX-17 | AKR1C4 | Aldo-keto reductase family 1 member C4 | P17516 |
| 346 | QLQX-17 | ALOX15 | Arachidonate 15-lipoxygenase | P16050 |
| 347 | QLQX-17 | AKR1B1 | Aldose reductase | P15121 |
| 348 | QLQX-17 | MMP9 | Matrix metalloproteinase-9 | P14780 |
| 349 | QLQX-17 | AKR1A1 | Alcohol dehydrogenase [NADP(+)] | P14550 |
| 350 | QLQX-17 | SRC | Proto-oncogene tyrosine-protein kinase Src | P12931 |
| 351 | QLQX-17 | PCNA | Proliferating cell nuclear antigen | P12004 |
| 352 | QLQX-17 | [ODC1](http://zinc15.docking.org/genes/ODC1) | Ornithine decarboxylase | P11926 |
| 353 | QLQX-17 | ALOX5 | Arachidonate 5-lipoxygenase | P09917 |
| 354 | QLQX-17 | FGR | Tyrosine-protein kinase Fgr | P09769 |
| 355 | QLQX-17 | MMP3 | Stromelysin-1 | P08254 |
| 356 | QLQX-17 | MMP2 | 72 kDa type IV collagenase | P08253 |
| 357 | QLQX-17 | YES1 | Tyrosine-protein kinase Yes | P07947 |
| 358 | QLQX-17 | CA3 | Carbonic anhydrase 3 | P07451 |
| 359 | QLQX-17 | [TH](http://zinc15.docking.org/genes/TH) | Tyrosine 3-monooxygenase | P07101 |
| 360 | QLQX-17 | FYN | Tyrosine-protein kinase Fyn | P06241 |
| 361 | QLQX-17 | APP | Amyloid-beta A4 protein | P05067 |
| 362 | QLQX-17 | MMP1 | Interstitial collagenase | P03956 |
| 363 | QLQX-17 | TTR | Transthyretin | P02766 |
| 364 | QLQX-17 | KCNK2 | Potassium channel subfamily K member 2 | O95069 |
| 365 | QLQX-17 | AKR1B10 | Aldo-keto reductase family 1 member B10 | O60218 |
| 366 | QLQX-17 | AlOX15B | Arachidonate 15-lipoxygenase B | O15296 |
| 367 | QLQX-17 | AKR1B15 | Aldo-keto reductase family 1 member B15 | C9JRZ8 |
| 368 | QLQX-17 | KDM4E | Lysine-specific demethylase 4E | B2RXH2 |
| 369 | QLQX-18 | KDM3A | Lysine-specific demethylase 3A | Q9Y4C1 |
| 370 | QLQX-18 | KDM2A | Lysine-specific demethylase 2A | Q9Y2K7 |
| 371 | QLQX-18 | CA5B | Carbonic anhydrase 5B, mitochondrial | Q9Y2D0 |
| 372 | QLQX-18 | [PLAA](http://zinc15.docking.org/genes/PLAA) | Phospholipase A-2-activating protein | Q9Y263 |
| 373 | QLQX-18 | NEU3 | Q9UQ49 | Q9UQ49 |
| 374 | QLQX-18 | SLC7A11 | Cystine/glutamate transporter | Q9UPY5 |
| 375 | QLQX-18 | PLA2G2D | Group IID secretory phospholipase A2 | Q9UNK4 |
| 376 | QLQX-18 | CA14 | Carbonic anhydrase 14 | Q9ULX7 |
| 377 | QLQX-18 | RPS6KB2 | Ribosomal protein S6 kinase beta-2 | Q9UBS0 |
| 378 | QLQX-18 | PLA2G2E | Group IIE secretory phospholipase A2 | Q9NZK7 |
| 379 | QLQX-18 | P4HTM | Transmembrane prolyl 4-hydroxylase | Q9NXG6 |
| 380 | QLQX-18 | TDP1 | Tyrosyl-DNA phosphodiesterase 1 | Q9NUW8 |
| 381 | QLQX-18 | GPR35 | G-protein coupled receptor 35 | Q9HC97 |
| 382 | QLQX-18 | KDM4C | Lysine-specific demethylase 4C | Q9H3R0 |
| 383 | QLQX-18 | [FTO](http://zinc15.docking.org/genes/FTO) | Alpha-ketoglutarate-dependent dioxygenase FTO | Q9C0B1 |
| 384 | QLQX-18 | HCAR1 | Hydroxycarboxylic acid receptor 1 | Q9BXC0 |
| 385 | QLQX-18 | DDO | D-aspartate oxidase | Q99489 |
| 386 | QLQX-18 | CAMKK2 | Calcium/calmodulin-dependent protein kinase kinase 2 | Q96RR4 |
| 387 | QLQX-18 | BHMT | Betaine--homocysteine S-methyltransferase 1 | Q93088 |
| 388 | QLQX-18 | SLC22A8 | Solute carrier family 22 member 8 | Q8TCC7 |
| 389 | QLQX-18 | CA13 | Carbonic anhydrase 13 | Q8N1Q1 |
| 390 | QLQX-18 | PARG | Poly(ADP-ribose) glycohydrolase | Q86W56 |
| 391 | QLQX-18 | SLC22A6 | Solute carrier family 22 member 6 | Q4U2R8 |
| 392 | QLQX-18 | CA9 | Carbonic anhydrase 9 | Q16790 |
| 393 | QLQX-18 | GRM3 | Metabotropic glutamate receptor 3 | Q14832 |
| 394 | QLQX-18 | SQLE | Squalene monooxygenase | Q14534 |
| 395 | QLQX-18 | GRM2 | Metabotropic glutamate receptor 2 | Q14416 |
| 396 | QLQX-18 | KCNMA1 | Calcium-activated potassium channel subunit alpha-1 | Q12791 |
| 397 | QLQX-18 | FUT7 | Alpha-(1,3)-fucosyltransferase 7 | Q11130 |
| 398 | QLQX-18 | ENPEP | Glutamyl aminopeptidase | Q07075 |
| 399 | QLQX-18 | [DNM1](http://zinc15.docking.org/genes/DNM1) | Dynamin-1 | Q05193 |
| 400 | QLQX-18 | AKR1C1 | Aldo-keto reductase family 1 member C1 | Q04828 |
| 401 | QLQX-18 | FOLH1 | Glutamate carboxypeptidase 2 | Q04609 |
| 402 | QLQX-18 | HSF1 | Heat shock factor protein 1 | Q00613 |
| 403 | QLQX-18 | ABAT | 4-aminobutyrate aminotransferase, mitochondrial | P80404 |
| 404 | QLQX-18 | LCN2 | Neutrophil gelatinase-associated lipocalin | P80188 |
| 405 | QLQX-18 | YWHAG | 14-3-3 protein gamma | P61981 |
| 406 | QLQX-18 | ARPP19 | cAMP-regulated phosphoprotein 19 | P56211 |
| 407 | QLQX-18 | GFER | FAD-linked sulfhydryl oxidase ALR | P55789 |
| 408 | QLQX-18 | AKR1C2 | Aldo-keto reductase family 1 member C2 | P52895 |
| 409 | QLQX-18 | ALDH5A1 | Succinate-semialdehyde dehydrogenase, mitochondrial | P51649 |
| 410 | QLQX-18 | TPMT | Thiopurine S-methyltransferase | P51580 |
| 411 | QLQX-18 | P2RX1 | P2X purinoceptor 1 | P51575 |
| 412 | QLQX-18 | DNM2 | Dynamin-2 | P50570 |
| 413 | QLQX-18 | HCAR3 | Asparagine synthetase [glutamine-hydrolyzing] | P49091 |
| 414 | QLQX-18 | CSNK1A1 | Casein kinase I isoform alpha | P48729 |
| 415 | QLQX-18 | RXRG | Retinoic acid receptor RXR-gamma | P48443 |
| 416 | QLQX-18 | XDH | Xanthine dehydrogenase/oxidase | P47989 |
| 417 | QLQX-18 | CA7 | Carbonic anhydrase 7 | P43166 |
| 418 | QLQX-18 | AKR1C3 | Aldo-keto reductase family 1 member C3 | P42330 |
| 419 | QLQX-18 | KDM5C | Lysine-specific demethylase 5C | P41229 |
| 420 | QLQX-18 | SNCA | Alpha-synuclein | P37840 |
| 421 | QLQX-18 | HSD17B2 | Estradiol 17-beta-dehydrogenase 2 | P37059 |
| 422 | QLQX-18 | CA5A | Carbonic anhydrase 5A, mitochondrial | P35218 |
| 423 | QLQX-18 | CA5A | Carbonic anhydrase 5A, mitochondrial | P35218 |
| 424 | QLQX-18 | ATIC | Bifunctional purine biosynthesis protein PURH | P31939 |
| 425 | QLQX-18 | SRD5A2 | 3-oxo-5-alpha-steroid 4-dehydrogenase 2 | P31213 |
| 426 | QLQX-18 | CDC25B | M-phase inducer phosphatase 2 | P30305 |
| 427 | QLQX-18 | CDC25A | M-phase inducer phosphatase 1 | P30304 |
| 428 | QLQX-18 | MPG | DNA-3-methyladenine glycosylase | P29372 |
| 429 | QLQX-18 | ADORA2B | Adenosine receptor A2b | P29275 |
| 430 | QLQX-18 | RXRB | Retinoic acid receptor RXR-beta | P28702 |
| 431 | QLQX-18 | DUSP1 | Dual specificity protein phosphatase 1 | P28562 |
| 432 | QLQX-18 | APEX1 | DNA-(apurinic or apyrimidinic site) lyase | P27695 |
| 433 | QLQX-18 | IGFBP5 | Insulin-like growth factor-binding protein 5 | P24593 |
| 434 | QLQX-18 | IGFBP6 | Insulin-like growth factor-binding protein 6 | P24592 |
| 435 | QLQX-18 | CA6 | Carbonic anhydrase 6 | P23280 |
| 436 | QLQX-18 | CES1 | Liver carboxylesterase 1 | P23141 |
| 437 | QLQX-18 | CA4 | Carbonic anhydrase 4 | P22748 |
| 438 | QLQX-18 | NR4A1 | Nuclear receptor subfamily 4 group A member 1 | P22736 |
| 439 | QLQX-18 | IGFBP4 | Insulin-like growth factor-binding protein 4 | P22692 |
| 440 | QLQX-18 | COMT | Catechol O-methyltransferase | P21964 |
| 441 | QLQX-18 | RXRA | Retinoic acid receptor RXR-alpha | P19793 |
| 442 | QLQX-18 | IGFBP2 | Insulin-like growth factor-binding protein 2 | P18065 |
| 443 | QLQX-18 | IGFBP3 | Insulin-like growth factor-binding protein 3 | P17936 |
| 444 | QLQX-18 | AKR1C4 | Aldo-keto reductase family 1 member C4 | P17516 |
| 445 | QLQX-18 | SELE | E-selectin | P16581 |
| 446 | QLQX-18 | ALOX15 | Arachidonate 15-lipoxygenase | P16050 |
| 447 | QLQX-18 | TYR | Tyrosinase | P14679 |
| 448 | QLQX-18 | SELL | L-selectin | P14151 |
| 449 | QLQX-18 | HSD17B1 | Estradiol 17-beta-dehydrogenase 1 | P14061 |
| 450 | QLQX-18 | P4HA1 | Prolyl 4-hydroxylase subunit alpha-1 | P13674 |
| 451 | QLQX-18 | RARG | Retinoic acid receptor gamma | P13631 |
| 452 | QLQX-18 | TOP1 | DNA topoisomerase 1 | P11387 |
| 453 | QLQX-18 | RARB | Retinoic acid receptor beta | P10826 |
| 454 | QLQX-18 | ALPG | Alkaline phosphatase, germ cell type | P10696 |
| 455 | QLQX-18 | MAPT | Microtubule-associated protein tau | P10636 |
| 456 | QLQX-18 | RARA | Retinoic acid receptor alpha | P10276 |
| 457 | QLQX-18 | PLA2G4B | Cytosolic phospholipase A2 beta | P0C869 |
| 458 | QLQX-18 | ALOX5 | Arachidonate 5-lipoxygenase | P09917 |
| 459 | QLQX-18 | QDPR | Dihydropteridine reductase | P09417 |
| 460 | QLQX-18 | DBH | Dopamine beta-hydroxylase | P09172 |
| 461 | QLQX-18 | IGFBP1 | Insulin-like growth factor-binding protein 1 | P08833 |
| 462 | QLQX-18 | GSTA1 | Glutathione S-transferase A1 | P08263 |
| 463 | QLQX-18 | ADRB2 | Beta-2 adrenergic receptor | P07550 |
| 464 | QLQX-18 | CA3 | Carbonic anhydrase 3 | P07451 |
| 465 | QLQX-18 | TH | Tyrosine 3-monooxygenase | P07101 |
| 466 | QLQX-18 | POLB | DNA polymerase beta | P06746 |
| 467 | QLQX-18 | [FYN](http://zinc15.docking.org/genes/FYN) | Tyrosine-protein kinase Fyn | P06241 |
| 468 | QLQX-18 | ALPL | Alkaline phosphatase, tissue-nonspecific isozyme | P05186 |
| 469 | QLQX-18 | SERPINE1 | Plasminogen activator inhibitor 1 | P05121 |
| 470 | QLQX-18 | PLA2G1B | Phospholipase A2 | P04054 |
| 471 | QLQX-18 | ESR1 | Estrogen receptor | P03372 |
| 472 | QLQX-18 | TTR | Transthyretin | P02766 |
| 473 | QLQX-18 | CA2 | Carbonic anhydrase 2 | P00918 |
| 474 | QLQX-18 | CA1 | Carbonic anhydrase 1 | P00915 |
| 475 | QLQX-18 | ERN1 | Serine/threonine-protein kinase/endoribonuclease IRE1 | O75460 |
| 476 | QLQX-18 | KDM4A | Lysine-specific demethylase 4A | O75164 |
| 477 | QLQX-18 | CA12 | Carbonic anhydrase 12 | O43570 |
| 478 | QLQX-18 | KDM6B | Lysine-specific demethylase 6B | O15054 |
| 479 | QLQX-18 | CES2 | Cocaine esterase | O00748 |
| 480 | QLQX-18 | [KDM4E](http://zinc15.docking.org/genes/KDM4E) | Lysine-specific demethylase 4E | B2RXH2 |
| 481 | QLQX-18 | SLC22A20 | Solute carrier family 22 member 20 | A6NK97 |
| 482 | QLQX-19 | [CA13](http://zinc15.docking.org/genes/CA13) | Carbonic anhydrase 13 | Q8N1Q1 |
| 483 | QLQX-19 | SLC6A3 | Sodium-dependent dopamine transporter | Q01959 |
| 484 | QLQX-19 | [CA7](http://zinc15.docking.org/genes/CA7) | Carbonic anhydrase 7 | P43166 |
| 485 | QLQX-19 | DRD3 | D(3) dopamine receptor | P35462 |
| 486 | QLQX-19 | [CA5A](http://zinc15.docking.org/genes/CA5A) | Carbonic anhydrase 5A, mitochondrial | P35218 |
| 487 | QLQX-19 | ADORA3 | Adenosine receptor A3 | P0DMS8 |
| 488 | QLQX-19 | SLC6A4 | Sodium-dependent serotonin transporter | P31645 |
| 489 | QLQX-19 | [SLC5A2](http://zinc15.docking.org/genes/SLC5A2) | Sodium/glucose cotransporter 2 | P31639 |
| 490 | QLQX-19 | SLC6A2 | Sodium-dependent noradrenaline transporter | P23975 |
| 491 | QLQX-19 | [CA4](http://zinc15.docking.org/genes/CA4) | Carbonic anhydrase 4 | P22748 |
| 492 | QLQX-19 | DRD5 | D(1B) dopamine receptor | P21918 |
| 493 | QLQX-19 | DRD1 | D(1A) dopamine receptor | P21728 |
| 494 | QLQX-19 | CHRM3 | Muscarinic acetylcholine receptor M3 | P20309 |
| 495 | QLQX-19 | ADRA2C | Alpha-2C adrenergic receptor | P18825 |
| 496 | QLQX-19 | ADRA2B | Alpha-2B adrenergic receptor | P18089 |
| 497 | QLQX-19 | CHRM1 | Muscarinic acetylcholine receptor M1 | P11229 |
| 498 | QLQX-19 | [PYGM](http://zinc15.docking.org/genes/PYGM) | Glycogen phosphorylase, muscle form | P11217 |
| 499 | QLQX-19 | ADRA2A | Alpha-2A adrenergic receptor | P08913 |
| 500 | QLQX-19 | CHRM5 | Muscarinic acetylcholine receptor M5 | P08912 |
| 501 | QLQX-19 | CHRM4 | Muscarinic acetylcholine receptor M4 | P08173 |
| 502 | QLQX-19 | CHRM2 | Muscarinic acetylcholine receptor M2 | P08172 |
| 503 | QLQX-19 | [CA3](http://zinc15.docking.org/genes/CA3) | Carbonic anhydrase 3 | P07451 |
| 504 | QLQX-19 | [ALDH2](http://zinc15.docking.org/genes/ALDH2) | Aldehyde dehydrogenase, mitochondrial | P05091 |
| 505 | QLQX-2 | PYGM | Glycogen phosphorylase, muscle form | Q9WUB3 |
| 506 | QLQX-2 | ABCG2 | ATP-binding cassette sub-family G member 2 | Q9UNQ0 |
| 507 | QLQX-2 | TDP1 | Tyrosyl-DNA phosphodiesterase 1 | Q9NUW8 |
| 508 | QLQX-2 | MBNL3 | Muscleblind-like protein 3 | Q9NUK0 |
| 509 | QLQX-2 | MBNL1 | Muscleblind-like protein 1 | Q9NR56 |
| 510 | QLQX-2 | NOX4 | NADPH oxidase 4 | Q9JHI8 |
| 511 | QLQX-2 | CA7 | Carbonic anhydrase 7 | Q9ERQ8 |
| 512 | QLQX-2 | SLC28A3 | Solute carrier family 28 member 3 | Q9ERH8 |
| 513 | QLQX-2 | CA13 | Carbonic anhydrase 13 | Q9D6N1 |
| 514 | QLQX-2 | SLC5A2 | Sodium/glucose cotransporter 2 | Q923I7 |
| 515 | QLQX-2 | ABCC2 | Canalicular multispecific organic anion transporter 1 | Q8VI47 |
| 516 | QLQX-2 | SLC5A11 | Sodium/myo-inositol cotransporter 2 | Q8K0E3 |
| 517 | QLQX-2 | CA12 | Carbonic anhydrase 12 | Q8CI85 |
| 518 | QLQX-2 | SLC5A1 | Sodium/glucose cotransporter 1 | Q8C3K6 |
| 519 | QLQX-2 | NMUR2 | Neuromedin-U receptor 2 | Q8BZ39 |
| 520 | QLQX-2 | KDM4A | Lysine-specific demethylase 4A | Q8BW72 |
| 521 | QLQX-2 | CA4 | Carbonic anhydrase 4 | Q64444 |
| 522 | QLQX-2 | HRAS | GTPase HRas | Q61411 |
| 523 | QLQX-2 | MBNL2 | Muscleblind-like protein 2 | Q5VZF2 |
| 524 | QLQX-2 | ALDH1L2 | Mitochondrial 10-formyltetrahydrofolate dehydrogenase | Q3SY69 |
| 525 | QLQX-2 | TOP1 | DNA topoisomerase 1 | Q04750 |
| 526 | QLQX-2 | XDH | Xanthine dehydrogenase/oxidase | Q00519 |
| 527 | QLQX-2 | ALOX5 | Arachidonate 5-lipoxygenase | P48999 |
| 528 | QLQX-2 | CBR1 | Carbonyl reductase [NADPH] 1 | P48758 |
| 529 | QLQX-2 | ALDH1A3 | Aldehyde dehydrogenase family 1 member A3 | P47895 |
| 530 | QLQX-2 | ALOX12 | Arachidonate 12-lipoxygenase, 12S-type | P39655 |
| 531 | QLQX-2 | AHR | Aryl hydrocarbon receptor | P35869 |
| 532 | QLQX-2 | HES1 | Transcription factor HES-1 | P35428 |
| 533 | QLQX-2 | CD22 | B-cell receptor CD22 | P35329 |
| 534 | QLQX-2 | ALDH1B1 | Aldehyde dehydrogenase X, mitochondrial | P30837 |
| 535 | QLQX-2 | ADORA1 | Adenosine receptor A1 | P30542 |
| 536 | QLQX-2 | GGTA1 | N-acetyllactosaminide alpha-1,3-galactosyltransferase | P23336 |
| 537 | QLQX-2 | ACHE | Acetylcholinesterase | P21836 |
| 538 | QLQX-2 | ABCB1A | Multidrug resistance protein 1A | P21447 |
| 539 | QLQX-2 | FASN | Fatty acid synthase | P19096 |
| 540 | QLQX-2 | LGALS3 | Galectin-3 | P16110 |
| 541 | QLQX-2 | LGALS1 | Galectin-1 | P16045 |
| 542 | QLQX-2 | B4GALT1 | Beta-1,4-galactosyltransferase 1 | P15535 |
| 543 | QLQX-2 | AKR1B1 | Aldose reductase | P15121 |
| 544 | QLQX-2 | TYR | Tyrosinase | P11344 |
| 545 | QLQX-2 | P4HB | Protein disulfide-isomerase | P09103 |
| 546 | QLQX-2 | [TNF](http://zinc15.docking.org/genes/TNF) | Tumor necrosis factor | P06804 |
| 547 | QLQX-2 | ALDH2 | Aldehyde dehydrogenase, mitochondrial | P05091 |
| 548 | QLQX-2 | Il5 | Interleukin-5 | P04401 |
| 549 | QLQX-2 | [IL2](http://zinc15.docking.org/genes/IL2) | Interleukin-2 | P04351 |
| 550 | QLQX-2 | ALDH1A1 | Retinal dehydrogenase 1 | P00352 |
| 551 | QLQX-2 | ALDH1A2 | Retinal dehydrogenase 2 | O94788 |
| 552 | QLQX-2 | ALDH1L1 | Cytosolic 10-formyltetrahydrofolate dehydrogenase | O75891 |
| 553 | QLQX-2 | LGALS7 | Galectin-7 | O54974 |
| 554 | QLQX-2 | LGALS9 | Galectin-9 | O08573 |
| 555 | QLQX-2 | AKR1B15 | Aldo-keto reductase family 1 member B15 | C9JRZ8 |
| 556 | QLQX-20 | SLCO1B1 | Solute carrier organic anion transporter family member 1B2 | [Q9Y6L6](http://www.uniprot.org/uniprot/Q9Y6L6) |
| 557 | QLQX-20 | RCE1 | CAAX prenyl protease 2 | Q9Y256 |
| 558 | QLQX-20 | ABCG2 | ATP-binding cassette sub-family G member 2 | [Q9UNQ0](http://www.uniprot.org/uniprot/Q9UNQ0) |
| 559 | QLQX-20 | CELA1 | Chymotrypsin-like elastase family member 1 | Q9UNI1 |
| 560 | QLQX-20 | RDH8 | Retinol dehydrogenase 8 | Q9NYR8 |
| 561 | QLQX-20 | TDP1 | Tyrosyl-DNA phosphodiesterase 1 | Q9NUW8 |
| 562 | QLQX-20 | MMP26 | Matrix metalloproteinase-26 | Q9NRE1 |
| 563 | QLQX-20 | NOX4 | NADPH oxidase 4 | Q9NPH5 |
| 564 | QLQX-20 | TNKS2 | Tankyrase-2 | Q9H2K2 |
| 565 | QLQX-20 | EGLN1 | Egl nine homolog 1 | Q9GZT9 |
| 566 | QLQX-20 | SENP7 | Sentrin-specific protease 7 | Q9BQF6 |
| 567 | QLQX-20 | GPER1 | G-protein coupled estrogen receptor 1 | Q99527 |
| 568 | QLQX-20 | NEU1 | Sialidase-1 | Q99519 |
| 569 | QLQX-20 | SLC22A12 | Solute carrier family 22 member 12 | Q96S37 |
| 570 | QLQX-20 | ESR2 | Estrogen receptor beta | Q92731 |
| 571 | QLQX-20 | CA13 | Carbonic anhydrase 13 | Q8N1Q1 |
| 572 | QLQX-20 | ANO1 | Anoctamin-1 | Q5XXA6 |
| 573 | QLQX-20 | CYP1B1 | Cytochrome P450 1B1 | Q16678 |
| 574 | QLQX-20 | NCOA1 | Nuclear receptor coactivator 1 | Q15788 |
| 575 | QLQX-20 | NCOA2 | Nuclear receptor coactivator 2 | [Q15596](http://www.uniprot.org/uniprot/Q15596) |
| 576 | QLQX-20 | ERBB4 | Receptor tyrosine-protein kinase erbB-4 | Q15303 |
| 577 | QLQX-20 | GRM7 | Metabotropic glutamate receptor 7 | Q14831 |
| 578 | QLQX-20 | PTK2B | Protein-tyrosine kinase 2-beta | Q14289 |
| 579 | QLQX-20 | DYRK1A | Dual specificity tyrosine-phosphorylation-regulated kinase 1A | Q13627 |
| 580 | QLQX-20 | CYP24A1 | 1,25-dihydroxyvitamin D(3) 24-hydroxylase, mitochondrial | Q07973 |
| 581 | QLQX-20 | PPARA | Peroxisome proliferator-activated receptor alpha | Q07869 |
| 582 | QLQX-20 | TOP2B | DNA topoisomerase 2-beta | Q02880 |
| 583 | QLQX-20 | CDK6 | Cyclin-dependent kinase 6 | Q00534 |
| 584 | QLQX-20 | CDK3 | Cyclin-dependent kinase 3 | Q00526 |
| 585 | QLQX-20 | HSD11B2 | Corticosteroid 11-beta-dehydrogenase isozyme 2 | P80365 |
| 586 | QLQX-20 | [IL2](http://zinc15.docking.org/genes/IL2) | Interleukin-2 | P60568 |
| 587 | QLQX-20 | TAS2R31 | Taste receptor type 2 member 31 | P59538 |
| 588 | QLQX-20 | DAPK1 | Death-associated protein kinase 1 | P53355 |
| 589 | QLQX-20 | GSK3B | Glycogen synthase kinase-3 beta | P49841 |
| 590 | QLQX-20 | MTNR1A | Melatonin receptor type 1A | P48039 |
| 591 | QLQX-20 | XDH | Xanthine dehydrogenase/oxidase | P47989 |
| 592 | QLQX-20 | NR4A2 | Nuclear receptor subfamily 4 group A member 2 | P43354 |
| 593 | QLQX-20 | CA7 | Carbonic anhydrase 7 | P43166 |
| 594 | QLQX-20 | HNF4A | Hepatocyte nuclear factor 4-alpha | P41235 |
| 595 | QLQX-20 | FEN1 | Flap endonuclease 1 | P39748 |
| 596 | QLQX-20 | PPARG | Peroxisome proliferator-activated receptor gamma | P37231 |
| 597 | QLQX-20 | HSD17B2 | Estradiol 17-beta-dehydrogenase 2 | P37059 |
| 598 | QLQX-20 | [HSD17B3](http://zinc15.docking.org/genes/HSD17B3) | Testosterone 17-beta-dehydrogenase 3 | P37058 |
| 599 | QLQX-20 | PTGS2 | Prostaglandin G/H synthase 2 | P35354 |
| 600 | QLQX-20 | GABRA3 | Gamma-aminobutyric acid receptor subunit alpha-3 | P34903 |
| 601 | QLQX-20 | ABCC1 | Multidrug resistance-associated protein 1 | P33527 |
| 602 | QLQX-20 | AKT1 | RAC-alpha serine/threonine-protein kinase | [P31749](http://www.uniprot.org/uniprot/P31749) |
| 603 | QLQX-20 | MPG | DNA-3-methyladenine glycosylase | P29372 |
| 604 | QLQX-20 | ERCC5 | DNA repair protein complementing XP-G cells | P28715 |
| 605 | QLQX-20 | GABRB3 | Gamma-aminobutyric acid receptor subunit beta-3 | P28472 |
| 606 | QLQX-20 | MAOB | Amine oxidase [flavin-containing] B | P27338 |
| 607 | QLQX-20 | ACP1 | Low molecular weight phosphotyrosine protein phosphatase | P24666 |
| 608 | QLQX-20 | PTGS1 | Prostaglandin G/H synthase 1 | P23219 |
| 609 | QLQX-20 | CA4 | Carbonic anhydrase 4 | P22748 |
| 610 | QLQX-20 | ACHE | Acetylcholinesterase | P22303 |
| 611 | QLQX-20 | LPO | Lactoperoxidase | P22079 |
| 612 | QLQX-20 | ERBB3 | Receptor tyrosine-protein kinase erbB-3 | P21860 |
| 613 | QLQX-20 | MAOA | Amine oxidase [flavin-containing] A | P21397 |
| 614 | QLQX-20 | CYP11B2 | Cytochrome P450 11B2, mitochondrial | P19099 |
| 615 | QLQX-20 | GABRG2 | Gamma-aminobutyric acid receptor subunit gamma-2 | P18507 |
| 616 | QLQX-20 | ALOX12 | Arachidonate 12-lipoxygenase, 12S-type | P18054 |
| 617 | QLQX-20 | CBR1 | Carbonyl reductase [NADPH] 1 | P16152 |
| 618 | QLQX-20 | ALOX15 | Arachidonate 15-lipoxygenase | P16050 |
| 619 | QLQX-20 | NQO1 | NAD(P)H dehydrogenase [quinone] 1 | P15559 |
| 620 | QLQX-20 | CYP11B1 | Cytochrome P450 11B1, mitochondrial | P15538 |
| 621 | QLQX-20 | AKR1B1 | Aldose reductase | P15121 |
| 622 | QLQX-20 | [TYR](http://zinc15.docking.org/genes/TYR) | Tyrosinase | P14679 |
| 623 | QLQX-20 | MIF | Macrophage migration inhibitory factor | P14174 |
| 624 | QLQX-20 | HSD17B1 | Estradiol 17-beta-dehydrogenase 1 | P14061 |
| 625 | QLQX-20 | CYP2C9 | Cytochrome P450 2C9 | [P11712](http://www.uniprot.org/uniprot/P11712) |
| 626 | QLQX-20 | CYP19A1 | Aromatase | P11511 |
| 627 | QLQX-20 | ESRRA | Steroid hormone receptor ERR1 | P11474 |
| 628 | QLQX-20 | TOP2A | DNA topoisomerase 2-alpha | [P11388](http://www.uniprot.org/uniprot/P11388) |
| 629 | QLQX-20 | MAPT | Microtubule-associated protein tau | P10636 |
| 630 | QLQX-20 | CYP2C8 | Cytochrome P450 2C8 | P10632 |
| 631 | QLQX-20 | CYP3A4 | Cytochrome P450 3A4 | [P08684](http://www.uniprot.org/uniprot/P08684) |
| 632 | QLQX-20 | [GUSB](http://zinc15.docking.org/genes/GUSB) | Beta-glucuronidase | P08236 |
| 633 | QLQX-20 | ABCB1 | Multidrug resistance protein 1 | P08183 |
| 634 | QLQX-20 | CA3 | Carbonic anhydrase 3 | P07451 |
| 635 | QLQX-20 | CYP1A2 | Cytochrome P450 1A2 | P05177 |
| 636 | QLQX-20 | IL5 | Interleukin-5 | P05113 |
| 637 | QLQX-20 | [ALDH2](http://zinc15.docking.org/genes/ALDH2) | Aldehyde dehydrogenase, mitochondrial | P05091 |
| 638 | QLQX-20 | APP | Amyloid-beta A4 protein | P05067 |
| 639 | QLQX-20 | ERBB2 | Receptor tyrosine-protein kinase erbB-2 | P04626 |
| 640 | QLQX-20 | SHBG | Sex hormone-binding globulin | [P04278](http://www.uniprot.org/uniprot/P04278) |
| 641 | QLQX-20 | ESR1 | Estrogen receptor | P03372 |
| 642 | QLQX-20 | TTR | Transthyretin | P02766 |
| 643 | QLQX-20 | EGFR | Epidermal growth factor receptor | P00533 |
| 644 | QLQX-20 | ESRRB | Steroid hormone receptor ERR2 | [O95718](http://www.uniprot.org/uniprot/O95718) |
| 645 | QLQX-20 | TNKS | Tankyrase-1 | O95271 |
| 646 | QLQX-20 | SLCO2B1 | Solute carrier organic anion transporter family member 2B1 | O94956 |
| 647 | QLQX-20 | TPPP | Tubulin polymerization-promoting protein | O94811 |
| 648 | QLQX-20 | CBR3 | Carbonyl reductase [NADPH] 3 | O75828 |
| 649 | QLQX-20 | NR1I2 | Nuclear receptor subfamily 1 group I member 2 | O75469 |
| 650 | QLQX-20 | AKR1B10 | Aldo-keto reductase family 1 member B10 | O60218 |
| 651 | QLQX-20 | SLC16A3 | Monocarboxylate transporter 4 | O15427 |
| 652 | QLQX-21 | SLCO1B1 | Solute carrier organic anion transporter family member 1B3 | Q9Y6L6 |
| 653 | QLQX-21 | ABCG2 | ATP-binding cassette sub-family G member 2 | [Q9UNQ0](http://www.uniprot.org/uniprot/Q9UNQ0) |
| 654 | QLQX-21 | CA14 | Carbonic anhydrase 14 | Q9ULX7 |
| 655 | QLQX-21 | TDP1 | Tyrosyl-DNA phosphodiesterase 1 | Q9NUW8 |
| 656 | QLQX-21 | SLCO1B3 | Solute carrier organic anion transporter family member 1B4 | Q9NPD5 |
| 657 | QLQX-21 | NMUR2 | Neuromedin-U receptor 2 | Q9GZQ4 |
| 658 | QLQX-21 | AURKB | Aurora kinase B | Q96GD4 |
| 659 | QLQX-21 | CA13 | Carbonic anhydrase 13 | Q8N1Q1 |
| 660 | QLQX-21 | SLC22A6 | Solute carrier family 22 member 6 | [Q4U2R8](http://www.uniprot.org/uniprot/Q4U2R8) |
| 661 | QLQX-21 | CYP1B1 | Cytochrome P450 1B1 | Q16678 |
| 662 | QLQX-21 | IL2 | Interleukin-2 | P60568 |
| 663 | QLQX-21 | [TAS2R31](http://zinc15.docking.org/genes/TAS2R31) | Taste receptor type 2 member 31 | P59538 |
| 664 | QLQX-21 | MTTP | Microsomal triglyceride transfer protein large subunit | P55157 |
| 665 | QLQX-21 | XDH | Xanthine dehydrogenase/oxidase | P47989 |
| 666 | QLQX-21 | MMP13 | Collagenase 3 | P45452 |
| 667 | QLQX-21 | CA7 | Carbonic anhydrase 7 | P43166 |
| 668 | QLQX-21 | ECE1 | Endothelin-converting enzyme 1 | P42892 |
| 669 | QLQX-21 | MMP12 | Macrophage metalloelastase | P39900 |
| 670 | QLQX-21 | SOAT1 | Sterol O-acyltransferase 1 | P35610 |
| 671 | QLQX-21 | CA5A | Carbonic anhydrase 5A, mitochondrial | P35218 |
| 672 | QLQX-21 | SLC5A2 | Sodium/glucose cotransporter 2 | P31639 |
| 673 | QLQX-21 | UGT1A1 | UDP-glucuronosyltransferase 1-1 | P22309 |
| 674 | QLQX-21 | ACHE | Acetylcholinesterase | P22303 |
| 675 | QLQX-21 | CD22 | B-cell receptor CD22 | P20273 |
| 676 | QLQX-21 | PNLIP | Pancreatic triacylglycerol lipase | P16233 |
| 677 | QLQX-21 | CBR1 | Carbonyl reductase [NADPH] 1 | P16152 |
| 678 | QLQX-21 | AKR1B1 | Aldose reductase | P15121 |
| 679 | QLQX-21 | TYR | Tyrosinase | P14679 |
| 680 | QLQX-21 | SLC5A1 | Sodium/glucose cotransporter 1 | P13866 |
| 681 | QLQX-21 | ODC1 | Ornithine decarboxylase | P11926 |
| 682 | QLQX-21 | CYP19A1 | Aromatase | P11511 |
| 683 | QLQX-21 | MMP10 | Stromelysin-2 | P09238 |
| 684 | QLQX-21 | MMP3 | Stromelysin-1 | P08254 |
| 685 | QLQX-21 | CA3 | Carbonic anhydrase 3 | P07451 |
| 686 | QLQX-21 | P4HB | Protein disulfide-isomerase | P07237 |
| 687 | QLQX-21 | CYP1A2 | Cytochrome P450 1A2 | P05177 |
| 688 | QLQX-21 | AMY2A | Pancreatic alpha-amylase | P04746 |
| 689 | QLQX-21 | SHBG | Sex hormone-binding globulin | P04278 |
| 690 | QLQX-21 | MMP1 | Interstitial collagenase | P03956 |
| 691 | QLQX-21 | CA2 | Carbonic anhydrase 2 | P00918 |
| 692 | QLQX-21 | CA1 | Carbonic anhydrase 1 | P00915 |
| 693 | QLQX-21 | SLCO2B1 | Solute carrier organic anion transporter family member 2B1 | O94956 |
| 694 | QLQX-21 | SOAT2 | Sterol O-acyltransferase 2 | [O75908](http://www.uniprot.org/uniprot/O75908) |
| 695 | QLQX-21 | KDM4A | Lysine-specific demethylase 4A | O75164 |
| 696 | QLQX-21 | CA12 | Carbonic anhydrase 12 | O43570 |
| 697 | QLQX-22 | ABCG2 | ATP-binding cassette sub-family G member 2 | Q9UNQ0 |
| 698 | QLQX-22 | TDP1 | Tyrosyl-DNA phosphodiesterase 1 | Q9NUW8 |
| 699 | QLQX-22 | MBNL3 | Muscleblind-like protein 3 | Q9NUK0 |
| 700 | QLQX-22 | MBNL1 | Muscleblind-like protein 1 | Q9NR56 |
| 701 | QLQX-22 | NOX4 | NADPH oxidase 4 | Q9NPH5 |
| 702 | QLQX-22 | NEK6 | Serine/threonine-protein kinase Nek6 | Q9HC98 |
| 703 | QLQX-22 | SLC28A3 | Solute carrier family 28 member 3 | Q9HAS3 |
| 704 | QLQX-22 | [NMUR2](http://zinc15.docking.org/genes/NMUR2) | Neuromedin-U receptor 2 | Q9GZQ4 |
| 705 | QLQX-22 | NEU1 | Sialidase-1 | Q99519 |
| 706 | QLQX-22 | AKR1E2 | 1,5-anhydro-D-fructose reductase | Q96JD6 |
| 707 | QLQX-22 | UGT3A1 | UDP-glucuronosyltransferase 3A1 | Q6NUS8 |
| 708 | QLQX-22 | MBNL2 | Muscleblind-like protein 2 | Q5VZF2 |
| 709 | QLQX-22 | CYP1B1 | Cytochrome P450 1B1 | Q16678 |
| 710 | QLQX-22 | PKN1 | Serine/threonine-protein kinase N1 | Q16512 |
| 711 | QLQX-22 | DYRK1A | Dual specificity tyrosine-phosphorylation-regulated kinase 1A | Q13627 |
| 712 | QLQX-22 | ST3GAL3 | CMP-N-acetylneuraminate-beta-1,4-galactoside alpha-2,3-sialyltransferase | Q11203 |
| 713 | QLQX-22 | GLO1 | Lactoylglutathione lyase | Q04760 |
| 714 | QLQX-22 | CDK6 | Cyclin-dependent kinase 6 | Q00534 |
| 715 | QLQX-22 | IL2 | Interleukin-2 | P60568 |
| 716 | QLQX-22 | DAPK1 | Death-associated protein kinase 1 | P53355 |
| 717 | QLQX-22 | RPS6KA3 | Ribosomal protein S6 kinase alpha-3 | P51812 |
| 718 | QLQX-22 | FASN | Fatty acid synthase | P49327 |
| 719 | QLQX-22 | XDH | Xanthine dehydrogenase/oxidase | P47989 |
| 720 | QLQX-22 | CA7 | Carbonic anhydrase 7 | P43166 |
| 721 | QLQX-22 | AKR1C3 | Aldo-keto reductase family 1 member C3 | P42330 |
| 722 | QLQX-22 | AHR | Aryl hydrocarbon receptor | P35869 |
| 723 | QLQX-22 | ABCC1 | Multidrug resistance-associated protein 1 | P33527 |
| 724 | QLQX-22 | SLC5A2 | Sodium/glucose cotransporter 2 | P31639 |
| 725 | QLQX-22 | MPG | DNA-3-methyladenine glycosylase | P29372 |
| 726 | QLQX-22 | ACP1 | Low molecular weight phosphotyrosine protein phosphatase | P24666 |
| 727 | QLQX-22 | CA4 | Carbonic anhydrase 4 | P22748 |
| 728 | QLQX-22 | ACHE | Acetylcholinesterase | P22303 |
| 729 | QLQX-22 | NT5E | 5'-nucleotidase | P21589 |
| 730 | QLQX-22 | CD22 | B-cell receptor CD22 | P20273 |
| 731 | QLQX-22 | ADRA2C | Alpha-2C adrenergic receptor | P18825 |
| 732 | QLQX-22 | ADRA2B | Alpha-2B adrenergic receptor | P18089 |
| 733 | QLQX-22 | CBR1 | Carbonyl reductase [NADPH] 1 | P16152 |
| 734 | QLQX-22 | NQO2 | Ribosyldihydronicotinamide dehydrogenase [quinone] | P16083 |
| 735 | QLQX-22 | ST6GAL1 | Beta-galactoside alpha-2,6-sialyltransferase 1 | P15907 |
| 736 | QLQX-22 | AKR1B1 | Aldose reductase | P15121 |
| 737 | QLQX-22 | TYR | Tyrosinase | P14679 |
| 738 | QLQX-22 | AKR1A1 | Alcohol dehydrogenase [NADP(+)] | P14550 |
| 739 | QLQX-22 | SLC5A1 | Sodium/glucose cotransporter 1 | P13866 |
| 740 | QLQX-22 | TOP1 | DNA topoisomerase 1 | P11387 |
| 741 | QLQX-22 | CYP2C8 | Cytochrome P450 2C8 | P10632 |
| 742 | QLQX-22 | ALPI | Intestinal-type alkaline phosphatase | P09923 |
| 743 | QLQX-22 | ALOX5 | Arachidonate 5-lipoxygenase | P09917 |
| 744 | QLQX-22 | ADRA2A | Alpha-2A adrenergic receptor | P08913 |
| 745 | QLQX-22 | CYP3A4 | Cytochrome P450 3A4 | P08684 |
| 746 | QLQX-22 | GUSB | Beta-glucuronidase | P08236 |
| 747 | QLQX-22 | ABCB1 | Multidrug resistance protein 1 | P08183 |
| 748 | QLQX-22 | [P4HB](http://zinc15.docking.org/genes/P4HB) | Protein disulfide-isomerase | P07237 |
| 749 | QLQX-22 | IL5 | Interleukin-5 | P05113 |
| 750 | QLQX-22 | AMY2A | Pancreatic alpha-amylase | P04746 |
| 751 | QLQX-22 | KDM4A | Lysine-specific demethylase 4A | O75164 |
| 752 | QLQX-22 | SLC16A7 | Monocarboxylate transporter 2 | O60669 |
| 753 | QLQX-22 | AKR1B10 | Aldo-keto reductase family 1 member B10 | O60218 |
| 754 | QLQX-22 | CA12 | Carbonic anhydrase 12 | O43570 |
| 755 | QLQX-22 | AKR1B15 | Aldo-keto reductase family 1 member B15 | C9JRZ8 |
| 756 | QLQX-23 | SLCO1B1 | Solute carrier organic anion transporter family member 1B4 | [Q9Y6L6](http://www.uniprot.org/uniprot/Q9Y6L6) |
| 757 | QLQX-23 | RUVBL2 | RuvB-like 2 | Q9Y230 |
| 758 | QLQX-23 | ABCG2 | ATP-binding cassette sub-family G member 2 | Q9UNQ0 |
| 759 | QLQX-23 | ALK | ALK tyrosine kinase receptor | Q9UM73 |
| 760 | QLQX-23 | CA14 | Carbonic anhydrase 14 | Q9ULX7 |
| 761 | QLQX-23 | NOX4 | NADPH oxidase 4 | Q9NPH5 |
| 762 | QLQX-23 | CA13 | Carbonic anhydrase 13 | Q9N1Q1 |
| 763 | QLQX-23 | NEK6 | Serine/threonine-protein kinase Nek6 | Q9HC98 |
| 764 | QLQX-23 | GPR35 | G-protein coupled receptor 35 | Q9HC97 |
| 765 | QLQX-23 | TNKS2 | Tankyrase-2 | Q9H2K2 |
| 766 | QLQX-23 | AMY1B | Alpha-amylase 1 | P04745 |
| 767 | QLQX-23 | AURKB | Aurora kinase B | Q96GD4 |
| 768 | QLQX-23 | ABCC2 | Canalicular multispecific organic anion transporter 1 | Q92887 |
| 769 | QLQX-23 | ESR2 | Estrogen receptor beta | Q92731 |
| 770 | QLQX-23 | POL | Integrase | Q7ZJM1 |
| 771 | QLQX-23 | HIBCH | 3-hydroxyisobutyryl-CoA hydrolase, mitochondrial | Q6NVY1 |
| 772 | QLQX-23 | UGT3A1 | UDP-glucuronosyltransferase 3A1 | Q6NUS8 |
| 773 | QLQX-23 | PFKFB3 | 6-phosphofructo-2-kinase/fructose-2,6-bisphosphatase 3 | Q16875 |
| 774 | QLQX-23 | CA9 | Carbonic anhydrase 9 | Q16790 |
| 775 | QLQX-23 | CYP1B1 | Cytochrome P450 1B1 | [Q16678](http://www.uniprot.org/uniprot/Q16678) |
| 776 | QLQX-23 | PKN1 | Serine/threonine-protein kinase N1 | Q16512 |
| 777 | QLQX-23 | SF3B3 | Splicing factor 3B subunit 3 | Q15393 |
| 778 | QLQX-23 | DYRK1A | Dual specificity tyrosine-phosphorylation-regulated kinase 1A | Q13627 |
| 779 | QLQX-23 | NAE1 | NEDD8-activating enzyme E1 regulatory subunit | Q13564 |
| 780 | QLQX-23 | CAMK2B | Calcium/calmodulin-dependent protein kinase type II subunit beta | Q13554 |
| 781 | QLQX-23 | [PTK2](http://zinc15.docking.org/genes/PTK2) | Focal adhesion kinase 1 | Q05397 |
| 782 | QLQX-23 | GLO1 | Lactoylglutathione lyase | Q04760 |
| 783 | QLQX-23 | CDK6 | Cyclin-dependent kinase 6 | Q00534 |
| 784 | QLQX-23 | CSNK2A1 | Casein kinase II subunit alpha | P68400 |
| 785 | QLQX-23 | CSNK2B | Casein kinase II subunit beta | P67870 |
| 786 | QLQX-23 | ACTB | Actin, cytoplasmic 1 | P60709 |
| 787 | QLQX-23 | IL2 | Interleukin-2 | P60568 |
| 788 | QLQX-23 | BACE1 | Beta-secretase 1 | P56817 |
| 789 | QLQX-23 | HSPA2 | Heat shock-related 70 kDa protein 2 | P54652 |
| 790 | QLQX-23 | SLC16A1 | Monocarboxylate transporter 1 | P53985 |
| 791 | QLQX-23 | DAPK1 | Death-associated protein kinase 1 | P53355 |
| 792 | QLQX-23 | NEK2 | Serine/threonine-protein kinase Nek2 | P51955 |
| 793 | QLQX-23 | RPS6KA3 | Ribosomal protein S6 kinase alpha-3 | P51812 |
| 794 | QLQX-23 | CCR4 | C-C chemokine receptor type 4 | P51679 |
| 795 | QLQX-23 | GSK3B | Glycogen synthase kinase-3 beta | P49841 |
| 796 | QLQX-23 | PGF | Placenta growth factor | P49763 |
| 797 | QLQX-23 | FASN | Fatty acid synthase | P49327 |
| 798 | QLQX-23 | PIK3CG | Phosphatidylinositol 4,5-bisphosphate 3-kinase catalytic subunit gamma isoform | [P48736](http://www.uniprot.org/uniprot/P48736) |
| 799 | QLQX-23 | [XDH](http://zinc15.docking.org/genes/XDH) | Xanthine dehydrogenase/oxidase | [P47989](http://www.uniprot.org/uniprot/Q16445) |
| 800 | QLQX-23 | MMP13 | Collagenase 3 | P45452 |
| 801 | QLQX-23 | CA7 | Carbonic anhydrase 7 | P43166 |
| 802 | QLQX-23 | OPRD1 | Delta-type opioid receptor | P41143 |
| 803 | QLQX-23 | [SNCA](http://zinc15.docking.org/genes/SNCA) | Alpha-synuclein | P37840 |
| 804 | QLQX-23 | HSD17B2 | Estradiol 17-beta-dehydrogenase 2 | P37059 |
| 805 | QLQX-23 | FLT3 | Receptor-type tyrosine-protein kinase FLT3 | P36888 |
| 806 | QLQX-23 | ATP5C1 | ATP synthase subunit gamma, mitochondrial | [P36542](http://www.uniprot.org/uniprot/P36542) |
| 807 | QLQX-23 | KDR | Vascular endothelial growth factor receptor 2 | P35968 |
| 808 | QLQX-23 | AHR | Aryl hydrocarbon receptor | P35869 |
| 809 | QLQX-23 | CA5A | Carbonic anhydrase 5A, mitochondrial | P35218 |
| 810 | QLQX-23 | ABCC1 | Multidrug resistance-associated protein 1 | [P33527](http://www.uniprot.org/uniprot/P33527) |
| 811 | QLQX-23 | AKT1 | RAC-alpha serine/threonine-protein kinase | P31749 |
| 812 | QLQX-23 | AXL | Tyrosine-protein kinase receptor UFO | P30530 |
| 813 | QLQX-23 | ADORA1 | Glucose-1-phosphate adenylyltransferase large subunit 1, chloroplastic/amyloplastic | P30524 |
| 814 | QLQX-23 | AVPR2 | Vasopressin V2 receptor | P30518 |
| 815 | QLQX-23 | MPG | DNA-3-methyladenine glycosylase | P29372 |
| 816 | QLQX-23 | ADORA2A | Adenosine receptor A2a | P29274 |
| 817 | QLQX-23 | PIK3R1 | Phosphatidylinositol 3-kinase regulatory subunit alpha | P27986 |
| 818 | QLQX-23 | APEX1 | DNA-(apurinic or apyrimidinic site) lyase | P27695 |
| 819 | QLQX-23 | ATP5A1 | ATP synthase subunit alpha, mitochondrial | P25705 |
| 820 | QLQX-23 | CXCR1 | C-X-C chemokine receptor type 1 | P25024 |
| 821 | QLQX-23 | ACP1 | Low molecular weight phosphotyrosine protein phosphatase | P24666 |
| 822 | QLQX-23 | JAK1 | Tyrosine-protein kinase JAK1 | [P23458](http://www.uniprot.org/uniprot/P23458) |
| 823 | QLQX-23 | CA6 | Carbonic anhydrase 6 | P23280 |
| 824 | QLQX-23 | CA4 | Carbonic anhydrase 4 | P22748 |
| 825 | QLQX-23 | UBA1 | Ubiquitin-like modifier-activating enzyme 1 | P22314 |
| 826 | QLQX-23 | DRD4 | D(4) dopamine receptor | P21917 |
| 827 | QLQX-23 | NT5E | 5'-nucleotidase | P21589 |
| 828 | QLQX-23 | MAOA | Amine oxidase [flavin-containing] A | P21397 |
| 829 | QLQX-23 | ALOX12 | Arachidonate 12-lipoxygenase, 12S-type | P18054 |
| 830 | QLQX-23 | CEBPB | CCAAT/enhancer-binding protein beta | P17676 |
| 831 | QLQX-23 | CREB1 | Cyclic AMP-responsive element-binding protein 1 | P16220 |
| 832 | QLQX-23 | CBR1 | Carbonyl reductase [NADPH] 1 | P16152 |
| 833 | QLQX-23 | NQO2 | Ribosyldihydronicotinamide dehydrogenase [quinone] | P16083 |
| 834 | QLQX-23 | ALOX15 | Arachidonate 15-lipoxygenase | P16050 |
| 835 | QLQX-23 | ST6GAL1 | Beta-galactoside alpha-2,6-sialyltransferase 1 | P15907 |
| 836 | QLQX-23 | VEGFA | Vascular endothelial growth factor A | P15692 |
| 837 | QLQX-23 | [AKR1B1](http://zinc15.docking.org/genes/AKR1B1) | Aldose reductase | P15121 |
| 838 | QLQX-23 | GABRA1 | Gamma-aminobutyric acid receptor subunit alpha-1 | P14867 |
| 839 | QLQX-23 | MMP9 | Matrix metalloproteinase-9 | P14780 |
| 840 | QLQX-23 | TYR | Tyrosinase | P14679 |
| 841 | QLQX-23 | AKR1A1 | Alcohol dehydrogenase [NADP(+)] | P14550 |
| 842 | QLQX-23 | CYP2C9 | Cytochrome P450 2C9 | P11712 |
| 843 | QLQX-23 | CYP19A1 | Aromatase | P11511 |
| 844 | QLQX-23 | ESRRA | Steroid hormone receptor ERR1 | [P11474](http://www.uniprot.org/uniprot/P11474) |
| 845 | QLQX-23 | TOP2A | DNA topoisomerase 2-alpha | P11388 |
| 846 | QLQX-23 | TOP1 | DNA topoisomerase 1 | P11387 |
| 847 | QLQX-23 | PIM1 | Serine/threonine-protein kinase pim-1 | P11309 |
| 848 | QLQX-23 | MAPT | Microtubule-associated protein tau | P10636 |
| 849 | QLQX-23 | CYP2C8 | Cytochrome P450 2C8 | P10632 |
| 850 | QLQX-23 | NR2F1 | COUP transcription factor 1 | P10589 |
| 851 | QLQX-23 | ALPI | Intestinal-type alkaline phosphatase | P09923 |
| 852 | QLQX-23 | ALOX5 | Arachidonate 5-lipoxygenase | P09917 |
| 853 | QLQX-23 | QDPR | Dihydropteridine reductase | P09417 |
| 854 | QLQX-23 | HCK | Tyrosine-protein kinase HCK | P08631 |
| 855 | QLQX-23 | MET | Hepatocyte growth factor receptor | P08581 |
| 856 | QLQX-23 | MMP3 | Stromelysin-1 | P08254 |
| 857 | QLQX-23 | MMP2 | 72 kDa type IV collagenase | P08253 |
| 858 | QLQX-23 | GUSB | Beta-glucuronidase | P08236 |
| 859 | QLQX-23 | ABCB1 | Multidrug resistance protein 1 | P08183 |
| 860 | QLQX-23 | IGF1R | Insulin-like growth factor 1 receptor | P08069 |
| 861 | QLQX-23 | HSP90AA1 | Heat shock protein HSP 90-alpha | P07900 |
| 862 | QLQX-23 | PRSS1 | Trypsin-1 | P07477 |
| 863 | QLQX-23 | CA3 | Carbonic anhydrase 3 | P07451 |
| 864 | QLQX-23 | P4HB | Protein disulfide-isomerase | P07237 |
| 865 | QLQX-23 | PYGL | Glycogen phosphorylase, liver form | P06737 |
| 866 | QLQX-23 | ATP5B | ATP synthase subunit beta, mitochondrial | P06576 |
| 867 | QLQX-23 | CDK1 | Cyclin-dependent kinase 1 | P06493 |
| 868 | QLQX-23 | CYP1A2 | Cytochrome P450 1A2 | P05177 |
| 869 | QLQX-23 | MPO | Myeloperoxidase | P05164 |
| 870 | QLQX-23 | IL5 | Interleukin-5 | P05113 |
| 871 | QLQX-23 | AMY1A | Alpha-amylase 1 | P04745 |
| 872 | QLQX-23 | ERBB2 | Receptor tyrosine-protein kinase erbB-2 | P04626 |
| 873 | QLQX-23 | SHBG | Sex hormone-binding globulin | [P04278](http://www.uniprot.org/uniprot/P04278) |
| 874 | QLQX-23 | PLA2G1B | Phospholipase A2 | P04054 |
| 875 | QLQX-23 | ESR1 | Estrogen receptor | P03372 |
| 876 | QLQX-23 | ALB | Serum albumin | P02768 |
| 877 | QLQX-23 | TTR | Transthyretin | P02766 |
| 878 | QLQX-23 | CA2 | Carbonic anhydrase 2 | P00918 |
| 879 | QLQX-23 | CA1 | Carbonic anhydrase 1 | P00915 |
| 880 | QLQX-23 | F2 | Prothrombin | P00734 |
| 881 | QLQX-23 | EGFR | Epidermal growth factor receptor | P00533 |
| 882 | QLQX-23 | ESRRB | Steroid hormone receptor ERR2 | O95718 |
| 883 | QLQX-23 | TNKS | Tankyrase-1 | O95271 |
| 884 | QLQX-23 | SLCO2B1 | Solute carrier organic anion transporter family member 2B1 | O94956 |
| 885 | QLQX-23 | STK17B | Serine/threonine-protein kinase 17B | O94768 |
| 886 | QLQX-23 | NR1I2 | Nuclear receptor subfamily 1 group I member 2 | O75469 |
| 887 | QLQX-23 | SLC16A7 | Monocarboxylate transporter 2 | O60669 |
| 888 | QLQX-23 | NUAK1 | NUAK family SNF1-like kinase 1 | O60285 |
| 889 | QLQX-23 | CA12 | Carbonic anhydrase 12 | O43570 |
| 890 | QLQX-23 | EIF3F | Eukaryotic translation initiation factor 3 subunit F | O00303 |
| 891 | QLQX-23 | KDM4E | Lysine-specific demethylase 4E | B2RXH2 |
| 892 | QLQX-23 | ALOX15B | Arachidonate 15-lipoxygenase B | O15296 |
| 893 | QLQX-24 | SCN10A | Sodium channel protein type 10 subunit alpha | Q9Y5Y9 |
| 894 | QLQX-24 | SCN11A | Sodium channel protein type 11 subunit alpha | Q9UI33 |
| 895 | QLQX-24 | MBNL3 | Muscleblind-like protein 3 | Q9NUK0 |
| 896 | QLQX-24 | KCNH7 | Potassium voltage-gated channel subfamily H member 7 | Q9NS40 |
| 897 | QLQX-24 | MBNL1 | Muscleblind-like protein 1 | Q9NR56 |
| 898 | QLQX-24 | KCNH6 | Potassium voltage-gated channel subfamily H member 6 | Q9H252 |
| 899 | QLQX-24 | SIGMAR1 | Sigma non-opioid intracellular receptor 1 | Q99720 |
| 900 | QLQX-24 | MBNL2 | Muscleblind-like protein 2 | Q5VZF2 |
| 901 | QLQX-24 | CHRFAM7A | CHRNA7-FAM7A fusion protein | Q494W8 |
| 902 | QLQX-24 | SCN5A | Sodium channel protein type 5 subunit alpha | [Q14524](http://www.uniprot.org/uniprot/Q14524) |
| 903 | QLQX-24 | KCNH2 | Tubulin beta-3 chain | Q12809 |
| 904 | QLQX-24 | SLC6A3 | Sodium-dependent dopamine transporter | Q01959 |
| 905 | QLQX-24 | CHRNA7 | Neuronal acetylcholine receptor subunit alpha-7 | P36544 |
| 906 | QLQX-24 | SLC6A4 | Sodium-dependent serotonin transporter | P31645 |
| 907 | QLQX-24 | CYP3A7 | Cytochrome P450 3A7 | [P24462](http://www.uniprot.org/uniprot/P24462) |
| 908 | QLQX-24 | SLC6A2 | Sodium-dependent noradrenaline transporter | P23975 |
| 909 | QLQX-24 | CES1 | Liver carboxylesterase 1 | P23141 |
| 910 | QLQX-24 | ACHE | Acetylcholinesterase | P22303 |
| 911 | QLQX-24 | CYP3A5 | Cytochrome P450 3A5 | P20815 |
| 912 | QLQX-24 | CHRM1 | Muscarinic acetylcholine receptor M1 | P11229 |
| 913 | QLQX-24 | CYP2D6 | Cytochrome P450 2D6 | P10635 |
| 914 | QLQX-24 | CYP2C8 | Cytochrome P450 2C8 | P10632 |
| 915 | QLQX-24 | CYP3A4 | Cytochrome P450 3A4 | P08684 |
| 916 | QLQX-24 | ADRB1 | Beta-1 adrenergic receptor | P08588 |
| 917 | QLQX-24 | [ABCB1](http://zinc15.docking.org/genes/ABCB1) | Multidrug resistance protein 1 | [P08183](http://www.uniprot.org/uniprot/P08183) |
| 918 | QLQX-24 | CHRM2 | Muscarinic acetylcholine receptor M2 | P08172 |
| 919 | QLQX-24 | ADRB2 | Beta-2 adrenergic receptor | P07550 |
| 920 | QLQX-24 | BCHE | Cholinesterase | P06276 |
| 921 | QLQX-24 | SLC22A2 | Solute carrier family 22 member 2 | O15244 |
| 922 | QLQX-25 | SCN10A | Sodium channel protein type 10 subunit alpha | Q9Y5Y9 |
| 923 | QLQX-25 | SCN11A | Sodium channel protein type 11 subunit alpha | Q9UI33 |
| 924 | QLQX-25 | MBNL3 | Muscleblind-like protein 3 | Q9NUK0 |
| 925 | QLQX-25 | KCNH7 | Potassium voltage-gated channel subfamily H member 7 | Q9NS40 |
| 926 | QLQX-25 | MBNL1 | Muscleblind-like protein 1 | Q9NR56 |
| 927 | QLQX-25 | KCNH6 | Potassium voltage-gated channel subfamily H member 6 | Q9H252 |
| 928 | QLQX-25 | SIGMAR1 | Sigma non-opioid intracellular receptor 1 | Q99720 |
| 929 | QLQX-25 | MBNL2 | Muscleblind-like protein 2 | Q5VZF2 |
| 930 | QLQX-25 | CHRFAM7A | CHRNA7-FAM7A fusion protein | Q494W8 |
| 931 | QLQX-25 | SCN5A | Sodium channel protein type 5 subunit alpha | [Q14524](http://www.uniprot.org/uniprot/Q14524) |
| 932 | QLQX-25 | KCNH2 | Tubulin beta-3 chain | Q12809 |
| 933 | QLQX-25 | SLC6A3 | Sodium-dependent dopamine transporter | Q01959 |
| 934 | QLQX-25 | CHRNA7 | Neuronal acetylcholine receptor subunit alpha-7 | P36544 |
| 935 | QLQX-25 | SLC6A4 | Sodium-dependent serotonin transporter | P31645 |
| 936 | QLQX-25 | CYP3A7 | Cytochrome P450 3A7 | [P24462](http://www.uniprot.org/uniprot/P24462) |
| 937 | QLQX-25 | SLC6A2 | Sodium-dependent noradrenaline transporter | P23975 |
| 938 | QLQX-25 | CES1 | Liver carboxylesterase 1 | P23141 |
| 939 | QLQX-25 | ACHE | Acetylcholinesterase | P22303 |
| 940 | QLQX-25 | CYP3A5 | Cytochrome P450 3A5 | P20815 |
| 941 | QLQX-25 | CHRM1 | Muscarinic acetylcholine receptor M1 | P11229 |
| 942 | QLQX-25 | CYP2D6 | Cytochrome P450 2D6 | P10635 |
| 943 | QLQX-25 | CYP2C8 | Cytochrome P450 2C8 | P10632 |
| 944 | QLQX-25 | CYP3A4 | Cytochrome P450 3A4 | P08684 |
| 945 | QLQX-25 | ADRB1 | Beta-1 adrenergic receptor | P08588 |
| 946 | QLQX-25 | [ABCB1](http://zinc15.docking.org/genes/ABCB1) | Multidrug resistance protein 1 | [P08183](http://www.uniprot.org/uniprot/P08183) |
| 947 | QLQX-25 | CHRM2 | Muscarinic acetylcholine receptor M2 | P08172 |
| 948 | QLQX-25 | ADRB2 | Beta-2 adrenergic receptor | P07550 |
| 949 | QLQX-25 | BCHE | Cholinesterase | P06276 |
| 950 | QLQX-25 | SLC22A2 | Solute carrier family 22 member 2 | O15244 |
| 951 | QLQX-26 | SCN10A | Sodium channel protein type 10 subunit alpha | Q9Y5Y9 |
| 952 | QLQX-26 | SCN11A | Sodium channel protein type 11 subunit alpha | Q9UI33 |
| 953 | QLQX-26 | KCNH7 | Potassium voltage-gated channel subfamily H member 7 | Q9NS40 |
| 954 | QLQX-26 | KCNH6 | Potassium voltage-gated channel subfamily H member 6 | Q9H252 |
| 955 | QLQX-26 | SIGMAR1 | Sigma non-opioid intracellular receptor 1 | Q99720 |
| 956 | QLQX-26 | CHRFAM7A | CHRNA7-FAM7A fusion protein | Q494W8 |
| 957 | QLQX-26 | SCN5A | Sodium channel protein type 5 subunit alpha | [Q14524](http://www.uniprot.org/uniprot/Q14524) |
| 958 | QLQX-26 | KCNH2 | Tubulin beta-3 chain | Q12809 |
| 959 | QLQX-26 | SLC6A3 | Sodium-dependent dopamine transporter | Q01959 |
| 960 | QLQX-26 | CHRNA7 | Neuronal acetylcholine receptor subunit alpha-7 | P36544 |
| 961 | QLQX-26 | CHRNA7 | Neuronal acetylcholine receptor subunit alpha-7 | [P36544](https://www.uniprot.org/uniprot/P36544) |
| 962 | QLQX-26 | SLC6A4 | Sodium-dependent serotonin transporter | P31645 |
| 963 | QLQX-26 | CYP3A7 | Cytochrome P450 3A7 | P24462 |
| 964 | QLQX-26 | SLC6A2 | Sodium-dependent noradrenaline transporter | P23975 |
| 965 | QLQX-26 | CES1 | Liver carboxylesterase 1 | P23141 |
| 966 | QLQX-26 | ACHE | Acetylcholinesterase | P22303 |
| 967 | QLQX-26 | CYP3A5 | Cytochrome P450 3A5 | P20815 |
| 968 | QLQX-26 | ADRB3 | Beta-3 adrenergic receptor | P13945 |
| 969 | QLQX-26 | CHRM1 | Muscarinic acetylcholine receptor M1 | P11229 |
| 970 | QLQX-26 | CYP2D6 | Cytochrome P450 2D6 | P10635 |
| 971 | QLQX-26 | CYP2C8 | Cytochrome P450 2C8 | P10632 |
| 972 | QLQX-26 | CYP3A4 | Cytochrome P450 3A4 | P08684 |
| 973 | QLQX-26 | ADRB1 | Beta-1 adrenergic receptor | P08588 |
| 974 | QLQX-26 | [ABCB1](http://zinc15.docking.org/genes/ABCB1) | Multidrug resistance protein 1 | [P08183](http://www.uniprot.org/uniprot/P08183) |
| 975 | QLQX-26 | CHRM4 | Muscarinic acetylcholine receptor M4 | P08173 |
| 976 | QLQX-26 | CHRM2 | Muscarinic acetylcholine receptor M2 | P08172 |
| 977 | QLQX-26 | ADRB2 | Beta-2 adrenergic receptor | P07550 |
| 978 | QLQX-26 | BCHE | Cholinesterase | P06276 |
| 979 | QLQX-26 | SLC22A2 | Solute carrier family 22 member 2 | O15244 |
| 980 | QLQX-27 | SCN10A | Sodium channel protein type 10 subunit alpha | Q9Y5Y9 |
| 981 | QLQX-27 | SCN11A | Sodium channel protein type 11 subunit alpha | Q9UI33 |
| 982 | QLQX-27 | KCNH7 | Potassium voltage-gated channel subfamily H member 7 | Q9NS40 |
| 983 | QLQX-27 | MBNL1 | Muscleblind-like protein 1 | Q9NR56 |
| 984 | QLQX-27 | KCNH6 | Potassium voltage-gated channel subfamily H member 6 | Q9H252 |
| 985 | QLQX-27 | SIGMAR1 | Sigma non-opioid intracellular receptor 1 | Q99720 |
| 986 | QLQX-27 | CHRFAM7A | CHRNA7-FAM7A fusion protein | Q494W8 |
| 987 | QLQX-27 | SCN5A | Sodium channel protein type 5 subunit alpha | [Q14524](http://www.uniprot.org/uniprot/Q14524) |
| 988 | QLQX-27 | KCNH2 | Tubulin beta-3 chain | Q12809 |
| 989 | QLQX-27 | SLC6A3 | Sodium-dependent dopamine transporter | Q01959 |
| 990 | QLQX-27 | HTR3A | 5-hydroxytryptamine receptor 3A | P46098 |
| 991 | QLQX-27 | CHRNA7 | Neuronal acetylcholine receptor subunit alpha-7 | P36544 |
| 992 | QLQX-27 | [CHRNA7](http://zinc15.docking.org/genes/CHRNA7) | Neuronal acetylcholine receptor subunit alpha-7 | [P36544](https://www.uniprot.org/uniprot/P36544) |
| 993 | QLQX-27 | SLC6A4 | Sodium-dependent serotonin transporter | P31645 |
| 994 | QLQX-27 | CYP3A7 | Cytochrome P450 3A7 | [P24462](http://www.uniprot.org/uniprot/P24462) |
| 995 | QLQX-27 | SLC6A2 | Sodium-dependent noradrenaline transporter | P23975 |
| 996 | QLQX-27 | CES1 | Liver carboxylesterase 1 | P23141 |
| 997 | QLQX-27 | ACHE | Acetylcholinesterase | P22303 |
| 998 | QLQX-27 | CYP3A5 | Cytochrome P450 3A5 | P20815 |
| 999 | QLQX-27 | ADRB3 | Beta-3 adrenergic receptor | P13945 |
| 1000 | QLQX-27 | CHRM1 | Muscarinic acetylcholine receptor M1 | P11229 |
| 1001 | QLQX-27 | CYP2D6 | Cytochrome P450 2D6 | P10635 |
| 1002 | QLQX-27 | CYP2C8 | Cytochrome P450 2C8 | P10632 |
| 1003 | QLQX-27 | CYP3A4 | Cytochrome P450 3A4 | P08684 |
| 1004 | QLQX-27 | ADRB1 | Beta-1 adrenergic receptor | P08588 |
| 1005 | QLQX-27 | [ABCB1](http://zinc15.docking.org/genes/ABCB1) | Multidrug resistance protein 1 | [P08183](http://www.uniprot.org/uniprot/P08183) |
| 1006 | QLQX-27 | CHRM2 | Muscarinic acetylcholine receptor M2 | P08172 |
| 1007 | QLQX-27 | ADRB2 | Beta-2 adrenergic receptor | P07550 |
| 1008 | QLQX-27 | BCHE | Cholinesterase | P06276 |
| 1009 | QLQX-27 | HTR3B | 5-hydroxytryptamine receptor 3B | O95264 |
| 1010 | QLQX-27 | SLC22A2 | Solute carrier family 22 member 2 | O15244 |
| 1011 | QLQX-28 | SCN10A | Sodium channel protein type 10 subunit alpha | Q9Y5Y9 |
| 1012 | QLQX-28 | SCN11A | Sodium channel protein type 11 subunit alpha | Q9UI33 |
| 1013 | QLQX-28 | MBNL3 | Muscleblind-like protein 3 | Q9NUK0 |
| 1014 | QLQX-28 | MBNL1 | Muscleblind-like protein 1 | Q9NR56 |
| 1015 | QLQX-28 | SIGMAR1 | Sigma non-opioid intracellular receptor 1 | [Q99720](http://www.uniprot.org/uniprot/Q99720) |
| 1016 | QLQX-28 | MBNL2 | Muscleblind-like protein 2 | Q5VZF2 |
| 1017 | QLQX-28 | CHRFAM7A | CHRNA7-FAM7A fusion protein | Q494W8 |
| 1018 | QLQX-28 | SCN5A | Sodium channel protein type 5 subunit alpha | Q14524 |
| 1019 | QLQX-28 | SLC6A3 | Sodium-dependent dopamine transporter | Q01959 |
| 1020 | QLQX-28 | SLC6A9 | Sodium- and chloride-dependent glycine transporter 1 | P48067 |
| 1021 | QLQX-28 | HTR3A | 5-hydroxytryptamine receptor 3A | P46098 |
| 1022 | QLQX-28 | CHRNA7 | Neuronal acetylcholine receptor subunit alpha-7 | P36544 |
| 1023 | QLQX-28 | ADORA3 | Adenosine receptor A3 | P0DMS8 |
| 1024 | QLQX-28 | SLC6A4 | Sodium-dependent serotonin transporter | [P31645](http://www.uniprot.org/uniprot/P31645) |
| 1025 | QLQX-28 | CYP3A7 | Cytochrome P450 3A7 | [P24462](http://www.uniprot.org/uniprot/P24462) |
| 1026 | QLQX-28 | SLC6A2 | Sodium-dependent noradrenaline transporter | P23975 |
| 1027 | QLQX-28 | CES1 | Liver carboxylesterase 1 | P23141 |
| 1028 | QLQX-28 | ACHE | Acetylcholinesterase | P22303 |
| 1029 | QLQX-28 | CYP3A5 | Cytochrome P450 3A5 | P20815 |
| 1030 | QLQX-28 | ADRB3 | Beta-3 adrenergic receptor | P13945 |
| 1031 | QLQX-28 | CHRM1 | Muscarinic acetylcholine receptor M1 | P11229 |
| 1032 | QLQX-28 | CYP2D6 | Cytochrome P450 2D6 | P10635 |
| 1033 | QLQX-28 | CYP2C8 | Cytochrome P450 2C8 | P10632 |
| 1034 | QLQX-28 | CYP3A4 | Cytochrome P450 3A4 | P08684 |
| 1035 | QLQX-28 | ADRB1 | Beta-1 adrenergic receptor | P08588 |
| 1036 | QLQX-28 | [ABCB1](http://zinc15.docking.org/genes/ABCB1) | Multidrug resistance protein 1 | [P08183](http://www.uniprot.org/uniprot/P08183) |
| 1037 | QLQX-28 | CHRM2 | Muscarinic acetylcholine receptor M2 | P08172 |
| 1038 | QLQX-28 | ADRB2 | Beta-2 adrenergic receptor | P07550 |
| 1039 | QLQX-28 | BCHE | Cholinesterase | P06276 |
| 1040 | QLQX-28 | HTR3B | 5-hydroxytryptamine receptor 3B | O95264 |
| 1041 | QLQX-28 | SLC22A2 | Solute carrier family 22 member 2 | O15244 |
| 1042 | QLQX-29 | SCN10A | Sodium channel protein type 10 subunit alpha | Q9Y5Y9 |
| 1043 | QLQX-29 | SCN11A | Sodium channel protein type 11 subunit alpha | Q9UI33 |
| 1044 | QLQX-29 | KCNH7 | Potassium voltage-gated channel subfamily H member 7 | Q9NS40 |
| 1045 | QLQX-29 | KCNH6 | Potassium voltage-gated channel subfamily H member 6 | Q9H252 |
| 1046 | QLQX-29 | SIGMAR1 | Sigma non-opioid intracellular receptor 1 | Q99720 |
| 1047 | QLQX-29 | CHRFAM7A | CHRNA7-FAM7A fusion protein | Q494W8 |
| 1048 | QLQX-29 | SCN5A | Sodium channel protein type 5 subunit alpha | [Q14524](http://www.uniprot.org/uniprot/Q14524) |
| 1049 | QLQX-29 | KCNH2 | Tubulin beta-3 chain | Q12809 |
| 1050 | QLQX-29 | SLC6A3 | Sodium-dependent dopamine transporter | Q01959 |
| 1051 | QLQX-29 | CHRNA7 | Neuronal acetylcholine receptor subunit alpha-7 | P36544 |
| 1052 | QLQX-29 | SLC6A4 | Sodium-dependent serotonin transporter | P31645 |
| 1053 | QLQX-29 | CYP3A7 | Cytochrome P450 3A7 | [P24462](http://www.uniprot.org/uniprot/P24462) |
| 1054 | QLQX-29 | SLC6A2 | Sodium-dependent noradrenaline transporter | P23975 |
| 1055 | QLQX-29 | CES1 | Liver carboxylesterase 1 | P23141 |
| 1056 | QLQX-29 | ACHE | Acetylcholinesterase | P22303 |
| 1057 | QLQX-29 | CYP3A5 | Cytochrome P450 3A5 | P20815 |
| 1058 | QLQX-29 | ADRB3 | Beta-3 adrenergic receptor | P13945 |
| 1059 | QLQX-29 | CHRM1 | Muscarinic acetylcholine receptor M1 | P11229 |
| 1060 | QLQX-29 | CYP2D6 | Cytochrome P450 2D6 | P10635 |
| 1061 | QLQX-29 | CYP2C8 | Cytochrome P450 2C8 | P10632 |
| 1062 | QLQX-29 | CYP3A4 | Cytochrome P450 3A4 | P08684 |
| 1063 | QLQX-29 | ADRB1 | Beta-1 adrenergic receptor | P08588 |
| 1064 | QLQX-29 | ABCB1 | Multidrug resistance protein 1 | P08183 |
| 1065 | QLQX-29 | CHRM2 | Muscarinic acetylcholine receptor M2 | P08172 |
| 1066 | QLQX-29 | ADRB2 | Beta-2 adrenergic receptor | P07550 |
| 1067 | QLQX-29 | BCHE | Cholinesterase | P06276 |
| 1068 | QLQX-29 | ABCB11 | Bile salt export pump | O95342 |
| 1069 | QLQX-29 | SLC22A2 | Solute carrier family 22 member 2 | O15244 |
| 1070 | QLQX-3 | IKBKG | NF-kappa-B essential modulator | Q9Y6K9 |
| 1071 | QLQX-3 | CA5B | Carbonic anhydrase 5B, mitochondrial | Q9Y2D0 |
| 1072 | QLQX-3 | [ABCG2](http://zinc15.docking.org/genes/ABCG2) | ATP-binding cassette sub-family G member 2 | Q9UNQ0 |
| 1073 | QLQX-3 | CA14 | Carbonic anhydrase 14 | Q9ULX7 |
| 1074 | QLQX-3 | [TUBB1](http://zinc15.docking.org/genes/TUBB1) | Tubulin beta-1 chain | Q9H4B7 |
| 1075 | QLQX-3 | SLC5A7 | High affinity choline transporter 1 | Q9GZV3 |
| 1076 | QLQX-3 | CA13 | Carbonic anhydrase 13 | Q8N1Q1 |
| 1077 | QLQX-3 | ABCB5 | ATP-binding cassette sub-family B member 5 | Q2M3G0 |
| 1078 | QLQX-3 | CA9 | Carbonic anhydrase 9 | Q16790 |
| 1079 | QLQX-3 | [NFE2L2](http://zinc15.docking.org/genes/NFE2L2) | Nuclear factor erythroid 2-related factor 2 | Q16236 |
| 1080 | QLQX-3 | GRM3 | Metabotropic glutamate receptor 3 | Q14832 |
| 1081 | QLQX-3 | [SLC29A2](http://zinc15.docking.org/genes/SLC29A2) | Equilibrative nucleoside transporter 2 | Q14542 |
| 1082 | QLQX-3 | GRM2 | Metabotropic glutamate receptor 2 | Q14416 |
| 1083 | QLQX-3 | DYRK1A | Dual specificity tyrosine-phosphorylation-regulated kinase 1A | Q13627 |
| 1084 | QLQX-3 | TUBB3 | Tubulin beta-3 chain | Q13509 |
| 1085 | QLQX-3 | CHRND | Acetylcholine receptor subunit delta | Q07001 |
| 1086 | QLQX-3 | DNM1 | Dynamin-1 | Q05193 |
| 1087 | QLQX-3 | [GLO1](http://zinc15.docking.org/genes/GLO1) | Lactoylglutathione lyase | Q04760 |
| 1088 | QLQX-3 | CA7 | Carbonic anhydrase 7 | P43166 |
| 1089 | QLQX-3 | PTGER2 | Prostaglandin E2 receptor EP2 subtype | P43116 |
| 1090 | QLQX-3 | AKR1C3 | Aldo-keto reductase family 1 member C3 | P42330 |
| 1091 | QLQX-3 | HSD17B3 | Testosterone 17-beta-dehydrogenase 3 | P37058 |
| 1092 | QLQX-3 | AHR | Aryl hydrocarbon receptor | P35869 |
| 1093 | QLQX-3 | NOS2 | Nitric oxide synthase, inducible | P35228 |
| 1094 | QLQX-3 | CA5A | Carbonic anhydrase 5A, mitochondrial | P35218 |
| 1095 | QLQX-3 | MAOB | Amine oxidase [flavin-containing] B | P27338 |
| 1096 | QLQX-3 | CA6 | Carbonic anhydrase 6 | P23280 |
| 1097 | QLQX-3 | CA4 | Carbonic anhydrase 4 | P22748 |
| 1098 | QLQX-3 | ABCB4 | Phosphatidylcholine translocator ABCB4 | P21439 |
| 1099 | QLQX-3 | MAOA | Amine oxidase [flavin-containing] A | P21397 |
| 1100 | QLQX-3 | CHRM3 | Muscarinic acetylcholine receptor M3 | P20309 |
| 1101 | QLQX-3 | [TNFRSF1A](http://zinc15.docking.org/genes/TNFRSF1A) | Tumor necrosis factor receptor superfamily member 1A | P19438 |
| 1102 | QLQX-3 | AKR1C4 | Aldo-keto reductase family 1 member C4 | P17516 |
| 1103 | QLQX-3 | AKR1B1 | Aldose reductase | P15121 |
| 1104 | QLQX-3 | ODC1 | Ornithine decarboxylase | P11926 |
| 1105 | QLQX-3 | [TOP2A](http://zinc15.docking.org/genes/TOP2A) | DNA topoisomerase 2-alpha | P11388 |
| 1106 | QLQX-3 | MAPT | Microtubule-associated protein tau | P10636 |
| 1107 | QLQX-3 | ALOX5 | Arachidonate 5-lipoxygenase | P09917 |
| 1108 | QLQX-3 | ABCB1 | Multidrug resistance protein 1 | P08183 |
| 1109 | QLQX-3 | CHRM2 | Muscarinic acetylcholine receptor M2 | P08172 |
| 1110 | QLQX-3 | CA3 | Carbonic anhydrase 3 | P07451 |
| 1111 | QLQX-3 | [APP](http://zinc15.docking.org/genes/APP) | Amyloid-beta A4 protein | P05067 |
| 1112 | QLQX-3 | ERBB2 | Receptor tyrosine-protein kinase erbB-2 | P04626 |
| 1113 | QLQX-3 | TTR | Transthyretin | P02766 |
| 1114 | QLQX-3 | CA2 | Carbonic anhydrase 2 | P00918 |
| 1115 | QLQX-3 | CA1 | Carbonic anhydrase 1 | P00915 |
| 1116 | QLQX-3 | ABCB11 | Bile salt export pump | O95342 |
| 1117 | QLQX-3 | [KCNK2](http://zinc15.docking.org/genes/KCNK2) | Potassium channel subfamily K member 2 | O95069 |
| 1118 | QLQX-3 | ERN1 | Serine/threonine-protein kinase/endoribonuclease IRE1 | O75460 |
| 1119 | QLQX-3 | [AKR1B10](http://zinc15.docking.org/genes/AKR1B10) | Aldo-keto reductase family 1 member B10 | O60218 |
| 1120 | QLQX-3 | PSMD14 | 26S proteasome non-ATPase regulatory subunit 14 | O00487 |
| 1121 | QLQX-4 | SLCO1B1 | Solute carrier organic anion transporter family member 1B1 | [Q9Y6L6](http://www.uniprot.org/uniprot/Q9Y6L6) |
| 1122 | QLQX-4 | HPSE | Heparanase | Q9Y251 |
| 1123 | QLQX-4 | SLCO1C1 | Solute carrier organic anion transporter family member 1C1 | Q9NYB5 |
| 1124 | QLQX-4 | SLCO1B3 | Solute carrier organic anion transporter family member 1B3 | Q9NPD5 |
| 1125 | QLQX-4 | ABCC2 | Canalicular multispecific organic anion transporter 1 | Q92887 |
| 1126 | QLQX-4 | HPSE2 | Inactive heparanase-2 | Q8WWQ2 |
| 1127 | QLQX-4 | SLC22A8 | Solute carrier family 22 member 8 | [Q8TCC7](http://www.uniprot.org/uniprot/Q8TCC7) |
| 1128 | QLQX-4 | SLCO4C1 | Solute carrier organic anion transporter family member 4C1 | Q6ZQN7 |
| 1129 | QLQX-4 | STAT4 | Signal transducer and activator of transcription 4 | Q14765 |
| 1130 | QLQX-4 | [LGALS4](http://zinc15.docking.org/genes/LGALS4) | Galectin-4 | P56470 |
| 1131 | QLQX-4 | STAT2 | Signal transducer and activator of transcription 2 | P52630 |
| 1132 | QLQX-4 | ATP1A2 | Sodium/potassium-transporting ATPase subunit alpha-2 | P50993 |
| 1133 | QLQX-4 | SLCO1A2 | Solute carrier organic anion transporter family member 1A2 | P46721 |
| 1134 | QLQX-4 | STAT1 | Signal transducer and activator of transcription 1-alpha/beta | P42224 |
| 1135 | QLQX-4 | STAT3 | Signal transducer and activator of transcription 3 | P40763 |
| 1136 | QLQX-4 | CYP2B6 | Cytochrome P450 2B6 | [P20813](http://www.uniprot.org/uniprot/P20813) |
| 1137 | QLQX-4 | CHRM3 | Muscarinic acetylcholine receptor M3 | P20309 |
| 1138 | QLQX-4 | VEGFA | Vascular endothelial growth factor A | P15692 |
| 1139 | QLQX-4 | ATP1A3 | Sodium/potassium-transporting ATPase subunit alpha-3 | P13637 |
| 1140 | QLQX-4 | CHRM1 | Muscarinic acetylcholine receptor M1 | P11229 |
| 1141 | QLQX-4 | MAPT | Microtubule-associated protein tau | P10636 |
| 1142 | QLQX-4 | FGF2 | Fibroblast growth factor 2 | P09038 |
| 1143 | QLQX-4 | CHRM5 | Muscarinic acetylcholine receptor M5 | P08912 |
| 1144 | QLQX-4 | CHRM4 | Muscarinic acetylcholine receptor M4 | P08173 |
| 1145 | QLQX-4 | CHRM2 | Muscarinic acetylcholine receptor M2 | P08172 |
| 1146 | QLQX-4 | FGF1 | Fibroblast growth factor 1 | P05230 |
| 1147 | QLQX-4 | ATP1A1 | Sodium/potassium-transporting ATPase subunit alpha-1 | P05023 |
| 1148 | QLQX-4 | CYP1A1 | Cytochrome P450 1A1 | P04798 |
| 1149 | QLQX-4 | [AMY2A](http://zinc15.docking.org/genes/AMY2A) | Pancreatic alpha-amylase | P04746 |
| 1150 | QLQX-4 | [LGALS8](http://zinc15.docking.org/genes/LGALS8) | Galectin-8 | O00214 |
| 1151 | QLQX-5 | SLCO1B1 | Solute carrier organic anion transporter family member 1B1 | [Q9Y6L6](http://www.uniprot.org/uniprot/Q9Y6L6) |
| 1152 | QLQX-5 | HPSE | Heparanase | Q9Y251 |
| 1153 | QLQX-5 | SLCO1C1 | Solute carrier organic anion transporter family member 1C1 | Q9NYB5 |
| 1154 | QLQX-5 | SLCO1B3 | Solute carrier organic anion transporter family member 1B3 | Q9NPD5 |
| 1155 | QLQX-5 | HPSE2 | Inactive heparanase-2 | Q8WWQ2 |
| 1156 | QLQX-5 | SLC22A8 | Solute carrier family 22 member 8 | [Q8TCC7](http://www.uniprot.org/uniprot/Q8TCC7) |
| 1157 | QLQX-5 | SLCO4C1 | Solute carrier organic anion transporter family member 4C1 | Q6ZQN7 |
| 1158 | QLQX-5 | STAT4 | Signal transducer and activator of transcription 4 | Q14765 |
| 1159 | QLQX-5 | STAT2 | Signal transducer and activator of transcription 2 | P52630 |
| 1160 | QLQX-5 | ATP1A2 | Sodium/potassium-transporting ATPase subunit alpha-2 | P50993 |
| 1161 | QLQX-5 | SLCO1A2 | Solute carrier organic anion transporter family member 1A2 | P46721 |
| 1162 | QLQX-5 | STAT1 | Signal transducer and activator of transcription 1-alpha/beta | P42224 |
| 1163 | QLQX-5 | STAT3 | Signal transducer and activator of transcription 3 | P40763 |
| 1164 | QLQX-5 | CYP2B6 | Cytochrome P450 2B6 | [P20813](http://www.uniprot.org/uniprot/P20813) |
| 1165 | QLQX-5 | CHRM3 | Muscarinic acetylcholine receptor M3 | P20309 |
| 1166 | QLQX-5 | VEGFA | Vascular endothelial growth factor A | P15692 |
| 1167 | QLQX-5 | ATP1A3 | Sodium/potassium-transporting ATPase subunit alpha-3 | P13637 |
| 1168 | QLQX-5 | CHRM1 | Muscarinic acetylcholine receptor M1 | P11229 |
| 1169 | QLQX-5 | MAPT | Microtubule-associated protein tau | P10636 |
| 1170 | QLQX-5 | FGF2 | Fibroblast growth factor 2 | P09038 |
| 1171 | QLQX-5 | CHRM5 | Muscarinic acetylcholine receptor M5 | P08912 |
| 1172 | QLQX-5 | CHRM4 | Muscarinic acetylcholine receptor M4 | P08173 |
| 1173 | QLQX-5 | CHRM2 | Muscarinic acetylcholine receptor M2 | P08172 |
| 1174 | QLQX-5 | FGF1 | Fibroblast growth factor 1 | P05230 |
| 1175 | QLQX-5 | ATP1A1 | Sodium/potassium-transporting ATPase subunit alpha-1 | P05023 |
| 1176 | QLQX-5 | CYP1A1 | Cytochrome P450 1A1 | P04798 |
| 1177 | QLQX-6 | SLCO1B1 | Solute carrier organic anion transporter family member 1B1 | [Q9Y6L6](http://www.uniprot.org/uniprot/Q9Y6L6) |
| 1178 | QLQX-6 | HPSE | Heparanase | Q9Y251 |
| 1179 | QLQX-6 | SLCO1C1 | Solute carrier organic anion transporter family member 1C1 | Q9NYB5 |
| 1180 | QLQX-6 | MBNL3 | Muscleblind-like protein 3 | Q9NUK0 |
| 1181 | QLQX-6 | MBNL1 | Muscleblind-like protein 1 | Q9NR56 |
| 1182 | QLQX-6 | SLCO1B3 | Solute carrier organic anion transporter family member 1B3 | Q9NPD5 |
| 1183 | QLQX-6 | HPSE2 | Inactive heparanase-2 | Q8WWQ2 |
| 1184 | QLQX-6 | SLC22A8 | Solute carrier family 22 member 8 | [Q8TCC7](http://www.uniprot.org/uniprot/Q8TCC7) |
| 1185 | QLQX-6 | SLCO4C1 | Solute carrier organic anion transporter family member 4C1 | Q6ZQN7 |
| 1186 | QLQX-6 | MBNL2 | Muscleblind-like protein 2 | Q5VZF2 |
| 1187 | QLQX-6 | STAT4 | Signal transducer and activator of transcription 4 | Q14765 |
| 1188 | QLQX-6 | STAT2 | Signal transducer and activator of transcription 2 | P52630 |
| 1189 | QLQX-6 | ATP1A2 | Sodium/potassium-transporting ATPase subunit alpha-2 | P50993 |
| 1190 | QLQX-6 | SLCO1A2 | Solute carrier organic anion transporter family member 1A2 | P46721 |
| 1191 | QLQX-6 | STAT1 | Signal transducer and activator of transcription 1-alpha/beta | P42224 |
| 1192 | QLQX-6 | STAT3 | Signal transducer and activator of transcription 3 | P40763 |
| 1193 | QLQX-6 | PTAFR | Platelet-activating factor receptor | P25105 |
| 1194 | QLQX-6 | CYP2B6 | Signal transducer and activator of transcription 1-alpha/beta | [P20813](http://www.uniprot.org/uniprot/P20813) |
| 1195 | QLQX-6 | VEGFA | Vascular endothelial growth factor A | P15692 |
| 1196 | QLQX-6 | ATP1A3 | Sodium/potassium-transporting ATPase subunit alpha-3 | P13637 |
| 1197 | QLQX-6 | FGF2 | Fibroblast growth factor 2 | P09038 |
| 1198 | QLQX-6 | CHRM5 | Muscarinic acetylcholine receptor M5 | P08912 |
| 1199 | QLQX-6 | FGF1 | Fibroblast growth factor 1 | P05230 |
| 1200 | QLQX-6 | ATP1A1 | Sodium/potassium-transporting ATPase subunit alpha-1 | P05023 |
| 1201 | QLQX-6 | CYP1A1 | Signal transducer and activator of transcription 3 | P04798 |
| 1202 | QLQX-6 | F2 | Prothrombin | P00734 |
| 1203 | QLQX-7 | SLCO1B1 | Solute carrier organic anion transporter family member 1B1 | [Q9Y6L6](http://www.uniprot.org/uniprot/Q9Y6L6) |
| 1204 | QLQX-7 | HPSE | Heparanase | Q9Y251 |
| 1205 | QLQX-7 | SLCO1C1 | Solute carrier organic anion transporter family member 1C1 | Q9NYB5 |
| 1206 | QLQX-7 | SLCO1B3 | Solute carrier organic anion transporter family member 1B3 | Q9NPD5 |
| 1207 | QLQX-7 | ABCC2 | Canalicular multispecific organic anion transporter 1 | Q92887 |
| 1208 | QLQX-7 | HPSE2 | Inactive heparanase-2 | Q8WWQ2 |
| 1209 | QLQX-7 | SLC22A8 | Solute carrier family 22 member 8 | [Q8TCC7](http://www.uniprot.org/uniprot/Q8TCC7) |
| 1210 | QLQX-7 | SLCO4C1 | Solute carrier organic anion transporter family member 4C1 | Q6ZQN7 |
| 1211 | QLQX-7 | STAT4 | Signal transducer and activator of transcription 4 | Q14765 |
| 1212 | QLQX-7 | [LGALS4](http://zinc15.docking.org/genes/LGALS4) | Galectin-4 | P56470 |
| 1213 | QLQX-7 | STAT2 | Signal transducer and activator of transcription 2 | P52630 |
| 1214 | QLQX-7 | ATP1A2 | Sodium/potassium-transporting ATPase subunit alpha-2 | P50993 |
| 1215 | QLQX-7 | SLCO1A2 | Solute carrier organic anion transporter family member 1A2 | P46721 |
| 1216 | QLQX-7 | STAT1 | Signal transducer and activator of transcription 1-alpha/beta | P42224 |
| 1217 | QLQX-7 | STAT3 | Signal transducer and activator of transcription 3 | P40763 |
| 1218 | QLQX-7 | DRD3 | D(3) dopamine receptor | P35462 |
| 1219 | QLQX-7 | ADRA1B | Alpha-1B adrenergic receptor | P35368 |
| 1220 | QLQX-7 | ADRA1A | Alpha-1A adrenergic receptor | P35348 |
| 1221 | QLQX-7 | ADRA1D | Alpha-1D adrenergic receptor | P25100 |
| 1222 | QLQX-7 | CYP2B6 | Signal transducer and activator of transcription 1-alpha/beta | [P20813](http://www.uniprot.org/uniprot/P20813) |
| 1223 | QLQX-7 | LGALS3 | Galectin-3 | P17931 |
| 1224 | QLQX-7 | VEGFA | Vascular endothelial growth factor A | P15692 |
| 1225 | QLQX-7 | DRD2 | D(2) dopamine receptor | P14416 |
| 1226 | QLQX-7 | ATP1A3 | Sodium/potassium-transporting ATPase subunit alpha-3 | P13637 |
| 1227 | QLQX-7 | FGF2 | Fibroblast growth factor 2 | P09038 |
| 1228 | QLQX-7 | CHRM5 | Muscarinic acetylcholine receptor M5 | P08912 |
| 1229 | QLQX-7 | FGF1 | Fibroblast growth factor 1 | P05230 |
| 1230 | QLQX-7 | ATP1A1 | Sodium/potassium-transporting ATPase subunit alpha-1 | P05023 |
| 1231 | QLQX-7 | CYP1A1 | Signal transducer and activator of transcription 3 | P04798 |
| 1232 | QLQX-7 | [LGALS8](http://zinc15.docking.org/genes/LGALS8) | Galectin-8 | O00214 |
| 1233 | QLQX-8 | SLCO1B1 | Solute carrier organic anion transporter family member 1B1 | [Q9Y6L6](http://www.uniprot.org/uniprot/Q9Y6L6) |
| 1234 | QLQX-8 | HPSE | Heparanase | Q9Y251 |
| 1235 | QLQX-8 | SLCO1C1 | Solute carrier organic anion transporter family member 1C1 | Q9NYB5 |
| 1236 | QLQX-8 | SLCO1B3 | Solute carrier organic anion transporter family member 1B3 | Q9NPD5 |
| 1237 | QLQX-8 | ABCC2 | Canalicular multispecific organic anion transporter 1 | Q92887 |
| 1238 | QLQX-8 | HPSE2 | Inactive heparanase-2 | Q8WWQ2 |
| 1239 | QLQX-8 | SLC22A8 | Solute carrier family 22 member 8 | [Q8TCC7](http://www.uniprot.org/uniprot/Q8TCC7) |
| 1240 | QLQX-8 | SLCO4C1 | Solute carrier organic anion transporter family member 4C1 | Q6ZQN7 |
| 1241 | QLQX-8 | STAT4 | Signal transducer and activator of transcription 4 | Q14765 |
| 1242 | QLQX-8 | [LGALS4](http://zinc15.docking.org/genes/LGALS4) | Galectin-4 | P56470 |
| 1243 | QLQX-8 | STAT2 | Signal transducer and activator of transcription 2 | P52630 |
| 1244 | QLQX-8 | ATP1A2 | Sodium/potassium-transporting ATPase subunit alpha-2 | P50993 |
| 1245 | QLQX-8 | SLCO1A2 | Solute carrier organic anion transporter family member 1A2 | P46721 |
| 1246 | QLQX-8 | STAT1 | Signal transducer and activator of transcription 1-alpha/beta | P42224 |
| 1247 | QLQX-8 | STAT3 | Signal transducer and activator of transcription 3 | P40763 |
| 1248 | QLQX-8 | CYP2B6 | Cytochrome P450 2B6 | [P20813](http://www.uniprot.org/uniprot/P20813) |
| 1249 | QLQX-8 | CHRM3 | Muscarinic acetylcholine receptor M3 | P20309 |
| 1250 | QLQX-8 | VEGFA | Vascular endothelial growth factor A | P15692 |
| 1251 | QLQX-8 | ATP1A3 | Sodium/potassium-transporting ATPase subunit alpha-3 | P13637 |
| 1252 | QLQX-8 | CHRM1 | Muscarinic acetylcholine receptor M1 | P11229 |
| 1253 | QLQX-8 | MAPT | Microtubule-associated protein tau | P10636 |
| 1254 | QLQX-8 | FGF2 | Fibroblast growth factor 2 | P09038 |
| 1255 | QLQX-8 | CHRM5 | Muscarinic acetylcholine receptor M5 | P08912 |
| 1256 | QLQX-8 | CHRM4 | Muscarinic acetylcholine receptor M4 | P08173 |
| 1257 | QLQX-8 | CHRM2 | Muscarinic acetylcholine receptor M2 | P08172 |
| 1258 | QLQX-8 | FGF1 | Fibroblast growth factor 1 | P05230 |
| 1259 | QLQX-8 | ATP1A1 | Sodium/potassium-transporting ATPase subunit alpha-1 | P05023 |
| 1260 | QLQX-8 | CYP1A1 | Cytochrome P450 1A1 | P04798 |
| 1261 | QLQX-8 | [AMY2A](http://zinc15.docking.org/genes/AMY2A) | Pancreatic alpha-amylase | P04746 |
| 1262 | QLQX-8 | [LGALS8](http://zinc15.docking.org/genes/LGALS8) | Galectin-8 | O00214 |
| 1263 | QLQX-9 | SLCO1B1 | Solute carrier organic anion transporter family member 1B1 | [Q9Y6L6](http://www.uniprot.org/uniprot/Q9Y6L6) |
| 1264 | QLQX-9 | HPSE | Heparanase | Q9Y251 |
| 1265 | QLQX-9 | SLCO1C1 | Solute carrier organic anion transporter family member 1C1 | Q9NYB5 |
| 1266 | QLQX-9 | SLCO1B3 | Solute carrier organic anion transporter family member 1B3 | Q9NPD5 |
| 1267 | QLQX-9 | HPSE2 | Inactive heparanase-2 | Q8WWQ2 |
| 1268 | QLQX-9 | SLC22A8 | Solute carrier family 22 member 8 | [Q8TCC7](http://www.uniprot.org/uniprot/Q8TCC7) |
| 1269 | QLQX-9 | SLCO4C1 | Solute carrier organic anion transporter family member 4C1 | Q6ZQN7 |
| 1270 | QLQX-9 | STAT4 | Signal transducer and activator of transcription 4 | Q14765 |
| 1271 | QLQX-9 | STAT2 | Signal transducer and activator of transcription 2 | P52630 |
| 1272 | QLQX-9 | ATP1A2 | Sodium/potassium-transporting ATPase subunit alpha-2 | P50993 |
| 1273 | QLQX-9 | SLCO1A2 | Solute carrier organic anion transporter family member 1A2 | P46721 |
| 1274 | QLQX-9 | STAT1 | Signal transducer and activator of transcription 1-alpha/beta | P42224 |
| 1275 | QLQX-9 | STAT3 | Signal transducer and activator of transcription 3 | P40763 |
| 1276 | QLQX-9 | CYP2B6 | Cytochrome P450 2B6 | [P20813](http://www.uniprot.org/uniprot/P20813) |
| 1277 | QLQX-9 | CHRM3 | Muscarinic acetylcholine receptor M3 | P20309 |
| 1278 | QLQX-9 | VEGFA | Vascular endothelial growth factor A | P15692 |
| 1279 | QLQX-9 | ATP1A3 | Sodium/potassium-transporting ATPase subunit alpha-3 | P13637 |
| 1280 | QLQX-9 | CHRM1 | Muscarinic acetylcholine receptor M1 | P11229 |
| 1281 | QLQX-9 | MAPT | Microtubule-associated protein tau | P10636 |
| 1282 | QLQX-9 | FGF2 | Fibroblast growth factor 2 | P09038 |
| 1283 | QLQX-9 | CHRM5 | Muscarinic acetylcholine receptor M5 | P08912 |
| 1284 | QLQX-9 | CHRM4 | Muscarinic acetylcholine receptor M4 | P08173 |
| 1285 | QLQX-9 | CHRM2 | Muscarinic acetylcholine receptor M2 | P08172 |
| 1286 | QLQX-9 | FGF1 | Fibroblast growth factor 1 | P05230 |
| 1287 | QLQX-9 | ATP1A1 | Sodium/potassium-transporting ATPase subunit alpha-1 | P05023 |
| 1288 | QLQX-9 | CYP1A1 | Cytochrome P450 1A1 | P04798 |
