# Supplementary material for: Integrating Pharmacokinetics Study, Network Analysis, and Experimental Validation to Uncover the Mechanism of Qiliqiangxin Capsule Against Chronic Heart Failure
Source: Front Pharmacol. 2019 Sep 18;10:1046. doi: 10.3389/fphar.2019.01046 (PMC6759796; doi:10.3389/fphar.2019.01046)
Supplement: Supplementary file 2 [file Table_2.doc]

**SUPPLEMENTARY TABLE S2 ׀** Candidate targets associated with CHF.

| **No.** | **Gene** | **Protein name** | **Uniprot ID** |
| --- | --- | --- | --- |
| 1 | CRHR2 | Corticotropin releasingfactor receptor 2 | Q13324 |
| 2 | TNNC1 | Troponin C | P63316 |
| 3 | CA2 | Carbonic anhydrase II | P00981 |
| 4 | HTR2B | 5-hydroxytryptamine 2B receptor | P41595 |
| 5 | SCNN1A | Amiloride-sensitive sodium channel subunit alpha | [P37088](http://www.uniprot.org/uniprot/P37088||P51168||P51170||P51172) |
| 6 | SCNN1B | Amiloride-sensitive sodium channel subunit beta | P51168 |
| 7 | SCNN1G | Amiloride-sensitive sodium channel subunit gamma | P51170 |
| 8 | SCNN1D | Amiloride-sensitive sodium channel subunit delta | P51172 |
| 9 | ADRA1D | Alpha-1D adrenergic receptor | P25100 |
| 10 | ASIC1 | Acid-sensing ion channel ASIC1a/ASIC1b | [P78348](http://www.uniprot.org/uniprot/P78348) |
| 11 | HTR4 | 5-hydroxytryptamine 4 receptor | [Q13639](http://www.uniprot.org/uniprot/Q13639) |
| 12 | NRG1 | Pro-neuregulin-1 | [Q02297](http://www.uniprot.org/uniprot/Q02297 (20-241)) |
| 13 | NR3C2 | Mineralocorticoid receptor | P08235 |
| 14 | ADRA1 | Alpha-1 adrenergic receptor | T81183 |
| 15 | ACE | Angiotensin-converting enzyme | P12821 |
| 16 | CYP11B1 | Cytochrome P450 11B1, mitochondrial | P15538 |
| 17 | SLC12A1 | Solute carrier family 12 member 1 | Q13621 |
| 18 | AVPR2 | Vasopressin V2 receptor | P30518 |
| 19 | AVPR1A | Vasopressin V1a receptor | P37288 |
| 20 | ADORA1 | Adenosine A1 receptor | P30542 |
| 21 | NPR3 | Natriuretic peptide receptor | P17342 |
| 22 | CA1 | [Carbonic anhydrase 1](https://www.drugbank.ca/biodb/bio_entities/BE0000267) | P00915 |
| 23 | AQP1 | [Aquaporin-1](https://www.drugbank.ca/biodb/bio_entities/BE0000880) | [P29972](http://www.uniprot.org/uniprot/P29972) |
| 24 | CA14 | [Carbonic anhydrase 14](https://www.drugbank.ca/biodb/bio_entities/BE0003634) | [Q9ULX7](http://www.uniprot.org/uniprot/Q9ULX7) |
| 25 | SLC22A6 | [Solute carrier family 22 member 6](https://www.drugbank.ca/biodb/bio_entities/BE0001066) | [Q4U2R8](http://www.uniprot.org/uniprot/Q4U2R8) |
| 26 | CA2 | [Carbonic anhydrase 2](https://www.drugbank.ca/biodb/bio_entities/BE0000322) | [P00918](http://www.uniprot.org/uniprot/P00918) |
| 27 | CA3 | [Carbonic anhydrase 3](https://www.drugbank.ca/biodb/bio_entities/BE0003625) | [P07451](http://www.uniprot.org/uniprot/P07451) |
| 28 | CA4 | Carbonic anhydrase 4 | P22748 |
| 29 | CYP3A4 | Cytochrome P450 3A4 | SLC22A6 |
| 30 | CA7 | Carbonic anhydrase 7 | P43166 |
| 31 | CA12 | Carbonic anhydrase 12 | O43570 |
| 32 | AOC1 | Amiloride-sensitive amine oxidase [copper-containing] | P19801 |
| 33 | SLC9A1 | Sodium/hydrogen exchanger 1 | P19634 |
| 34 | ASIC2 | Acid-sensing ion channel 2 | Q16515 |
| 35 | PLAU | Urokinase-type plasminogen activator | [P00749](http://www.uniprot.org/uniprot/P00749) |
| 36 | SLC22A2 | Solute carrier family 22 member 2 | O15244 |
| 37 | SLC22A4 | Solute carrier family 22 member 4 | Q9H015 |
| 38 | AOC1 | Amiloride-sensitive amine oxidase [copper-containing] | Q9TRC7 |
| 39 | HMGCR | 3-hydroxy-3-methylglutaryl-coenzyme A reductase | P04035 |
| 40 | CYP3A4 | Cytochrome P450 3A4 | P08684 |
| 41 | DPP4 | Dipeptidyl peptidase 4 | P27487 |
| 42 | CYP2D6 | Cytochrome P450 2D6 | P10635 |
| 43 | CYP2C9 | Cytochrome P450 2C9 | P11712 |
| 44 | CYP2C19 | Cytochrome P450 2C19 | P33261 |
| 45 | CYP3A5 | Cytochrome P450 3A5 | P20815 |
| 46 | CYP3A7 | Cytochrome P450 3A7 | P24462 |
| 47 | ABCB1 | Multidrug resistance protein 1 | P08183 |
| 48 | SLCO1A2 | Solute carrier organic anion transporter family member 1A2 | P46721 |
| 49 | SLCO1B1 | Solute carrier organic anion transporter family member 1B1 | Q9Y6L6 |
| 50 | ABCC4 | Multidrug resistance-associated protein 4 | O15439 |
| 51 | ABCC5 | Multidrug resistance-associated protein 5 | O15440 |
| 52 | ABCC1 | Multidrug resistance-associated protein 1 | P33527 |
| 53 | CYP2B6 | Cytochrome P450 2B6 | P20813 |
| 54 | CYP2C8 | Cytochrome P450 2C8 | P10632 |
| 55 | AHR | Aryl hydrocarbon receptor | P35869 |
| 56 | SLCO2B1 | Solute carrier organic anion transporter family member 2B1 | O94956 |
| 57 | SLCO1B3 | Solute carrier organic anion transporter family member 1B3 | Q9NPD5 |
| 58 | UGT1A1 | UDP-glucuronosyltransferase 1-1 | P22309 |
| 59 | UGT1A3 | UDP-glucuronosyltransferase 1-3 | P35503 |
| 60 | UGT2B7 | UDP-glucuronosyltransferase 2B7 | P16662 |
| 61 | ABCC2 | Canalicular multispecific organic anion transporter 1 | Q92887 |
| 62 | ABCB11 | Bile salt export pump | O95342 |
| 63 | ALB | Serum albumin | P02768 |
| 64 | MMP2 | 72 kDa type IV collagenase | P08253 |
| 65 | MMP9 | Matrix metalloproteinase-9 | P14780 |
| 66 | SLC15A1 | Solute carrier family 15 member 1 | P46059 |
| 67 | LTA4H | Leukotriene A-4 hydrolase | P09960 |
| 68 | BDKRB1 | B1 bradykinin receptor | P46663 |
| 69 | SLC15A2 | Solute carrier family 15 member 2 | Q16348 |
| 70 | AGTR1 | Type-1 angiotensin II receptor | P30556 |
| 71 | ATP1A1 | Sodium/potassium-transporting ATPase subunit alpha-1 | P05023 |
| 72 | GSTA2 | Glutathione S-transferase A2 | P09210 |
| 73 | LEF1 | Lymphoid enhancer-binding factor 1 | Q9UJU2 |
| 74 | GSTP1 | Glutathione S-transferase P | P09211 |
| 75 | SLC22A5 | Solute carrier family 22 member 5 | O76082 |
| 76 | SLC22A8 | Solute carrier family 22 member 8 | [Q8TCC7](http://www.uniprot.org/uniprot/Q8TCC7) |
| 77 | SLCO2A1 | Solute carrier organic anion transporter family member 2A1 | Q92959 |
| 78 | SLC22A11 | Solute carrier family 22 member 11 | Q9NSA0 |
| 79 | PGD | 6-phosphogluconate dehydrogenase, decarboxylating | P52209 |
| 80 | SLC12A3 | Solute carrier family 12 member 3 | P55017 |
| 81 | KCNMA1 | Calcium-activated potassium channel subunit alpha-1 | Q12791 |
| 82 | ADRB1 | Beta-1 adrenergic receptor | P08588 |
| 83 | ADRB2 | Beta-2 adrenergic receptor | P07550 |
| 84 | PIK3R1 | Phosphatidylinositol 3-kinase regulatory subunit alpha | P27986 |
| 85 | ADRB3 | Beta-3 adrenergic receptor | P13945 |
| 86 | MAPK1 | Mitogen-activated protein kinase 1 | P28482 |
| 87 | PIK3R2 | Phosphatidylinositol 3-kinase regulatory subunit beta | O00459 |
| 88 | PIK3R3 | Phosphatidylinositol 3-kinase regulatory subunit gamma | Q92569 |
| 89 | CYP1A1 | Cytochrome P450 1A1 | P04798 |
| 90 | PDE4 | CAMP phosphodiesterase | Q4ZHU6 |
| 91 | SOD1 | Superoxide dismutase [Cu-Zn] | P00441 |
| 92 | PDE3A | cGMP-inhibited 3',5'-cyclic phosphodiesterase A | Q14432 |
| 93 | NPR1 | Atrial natriuretic peptide receptor 1 | P16066 |
| 94 | ALDH2 | Aldehyde dehydrogenase, mitochondrial | P05091 |
| 95 | PLAUR | Urokinase plasminogen activator surface receptor | Q03405 |
| 96 | FGA | Fibrinogen alpha chain | P02671 |
| 97 | PLG | Plasminogen | P00747 |
| 98 | SERPINE1 | Plasminogen activator inhibitor 1 | P05121 |
| 99 | AR | Androgen receptor | P10275 |
| 100 | PGR | Progesterone receptor | P06401 |
| 101 | NR3C1 | Glucocorticoid receptor | P04150 |
| 102 | CYP11B2 | Cytochrome P450 11B2, mitochondrial | P19099 |
| 103 | SRD5A1 | 3-oxo-5-alpha-steroid 4-dehydrogenase 1 | P18405 |
| 104 | SRD5A2 | 3-oxo-5-alpha-steroid 4-dehydrogenase 2 | P31213 |
| 105 | SRD5A3 | Polyprenol reductase | Q9H8P0 |
| 106 | SHBG | Sex hormone-binding globulin | P04278 |
| 107 | CACNG1 | Voltage-dependent calcium channel gamma-1 subunit | Q06432 |
| 108 | CACNA2D1 | Voltage-dependent calcium channel subunit alpha-2/delta-1 | P54289 |
| 109 | CACNA2D2 | Voltage-dependent calcium channel subunit alpha-2/delta-2 | Q9NY47 |
| 110 | CACNA2D3 | Voltage-dependent calcium channel subunit alpha-2/delta-3 | Q8IZS8 |
| 111 | CACNA1C | Voltage-dependent L-type calcium channel subunit alpha-1C | Q13936 |
| 112 | CACNA1D | Voltage-dependent L-type calcium channel subunit alpha-1D | Q01668 |
| 113 | CACNA1F | Voltage-dependent L-type calcium channel subunit alpha-1F | O60840 |
| 114 | CACNA1S | Voltage-dependent L-type calcium channel subunit alpha-1S | Q13698 |
| 115 | CACNB1 | Voltage-dependent L-type calcium channel subunit beta-1 | Q6TME4 |
| 116 | CACNB1 | Voltage-dependent L-type calcium channel subunit beta-1 | Q02641 |
| 117 | CACNB2 | Voltage-dependent L-type calcium channel subunit beta-2 | Q08289 |
| 118 | CACNB3 | Voltage-dependent L-type calcium channel subunit beta-3 | P54284 |
| 119 | CACNB4 | Voltage-dependent L-type calcium channel subunit beta-4 | O00305 |
| 120 | CACNA1B | Voltage-dependent N-type calcium channel subunit alpha-1B | Q00975 |
| 121 | CACNA1A | Voltage-dependent P/Q-type calcium channel subunit alpha-1A | O00555 |
| 122 | CACNA1G | Voltage-dependent T-type calcium channel subunit alpha-1G | O43497 |
| 123 | CACNA1H | Voltage-dependent T-type calcium channel subunit alpha-1H | O95180 |
| 124 | CACNA1I | Voltage-dependent T-type calcium channel subunit alpha-1I | Q9P0X4 |
| 125 | NR1I2 | Nuclear receptor subfamily 1 group I member 2 | O75469 |
| 126 | PTGS1 | Prostaglandin G/H synthase 1 | P23219 |
| 127 | CYP1A2 | Cytochrome P450 1A2 | P05177 |
| 128 | SLC22A2 | Solute carrier family 22 member 2 | Q15244 |
| 129 | SLC22A1 | Solute carrier family 22 member 1 | Q15245 |
| 130 | ABCC4 | Multidrug resistance-associated protein 4 | Q15439 |
| 131 | EDN1 | endothelin 1 | P05305 |
| 132 | TNF | tumor necrosis factor | P01375 |
| 133 | REN | renin | P00797 |
| 134 | MSTN | myostatin | O14793 |
| 135 | RYR2 | ryanodine receptor 2 | Q92736 |
| 136 | ACE2 | angiotensin I converting enzyme 2 | Q9BYF1 |
| 137 | NOS2 | nitric oxide synthase 2 | P35228 |
| 138 | VEGFA | vascular endothelial growth factor A | P15692 |
| 139 | CXCR4 | C-X-C motif chemokine receptor 4 | P61073 |
| 140 | TBPL1 | TATA-box binding protein like 1 | P62380 |
| 141 | PNMT | phenylethanolamine N-methyltransferase | P11086 |
| 142 | NPPC | natriuretic peptide C | P23582 |
| 143 | NOS1AP | nitric oxide synthase 1 adaptor protein | O75052 |
| 144 | MUC2 | mucin 2, oligomeric mucus/gel-forming | Q02817 |
| 145 | MECP2 | methyl-CpG binding protein 2 | P51608 |
| 146 | CD14 | CD14 molecule | P08571 |
| 147 | HSD17B6 | hydroxysteroid 17-beta dehydrogenase 6 | O14756 |
| 148 | CCL2 | C-C motif chemokine ligand 2 | P13500 |
| 149 | ADIPOR2 | adiponectin receptor 2 | Q86V24 |
| 150 | ADM2 | adrenomedullin 2 | Q7Z4H4 |
| 151 | VPS51 | VPS51, GARP complex subunit | Q9UID3 |
| 152 | TP53 | tumor protein p53 | P04637 |
| 153 | CALM1 | calmodulin 1 | P62158 |
| 154 | PIF1 | PIF1 5'-to-3' DNA helicase | Q9H611 |
| 155 | TERF2 | telomeric repeat binding factor 2 | Q15554 |
| 156 | MARCKSL1 | MARCKS like 1 | P49006 |
| 157 | CSRP3 | cysteine and glycine rich protein 3 | P50461 |
| 158 | CXCL12 | C-X-C motif chemokine ligand 12 | P48061 |
| 159 | EDNRB | endothelin receptor type B | P24530 |
| 160 | ADA | adenosine deaminase | P00813 |
| 161 | AGT | angiotensinogen | P01019 |
| 162 | GRK2 | G protein-coupled receptor kinase 2 | P25098 |
| 163 | CTSK | cathepsin K | P43235 |
| 164 | ZFP90 | ZFP90 zinc finger protein | Q8TF47 |
| 165 | ADORA2B | adenosine A2b receptor | P29275 |
| 166 | ADORA2A | adenosine A2a receptor | P29274 |
| 167 | ADM | adrenomedullin | P35318 |
| 168 | DCD | dermcidin | P81605 |
| 169 | VSIG4 | V-set and immunoglobulin domain containing 4 | Q9Y279 |
| 170 | ERBB2 | erb-b2 receptor tyrosine kinase 2 | P04626 |
| 171 | SIRT1 | sirtuin 1 | Q96EB6 |
| 172 | GHRH | growth hormone releasing hormone | P01286 |
| 173 | LOX | lysyl oxidase | P28300 |
| 174 | KCNE1 | potassium voltage-gated channel subfamily E regulatory subunit 1 | P15382 |
| 175 | IFNG | interferon gamma | P01579 |
| 176 | NANOS2 | nanos C2HC-type zinc finger 2 | P60321 |
| 177 | HSPA1B | heat shock protein family A (Hsp70) member 1B | P0DMV8 |
| 178 | HSPA1B | Heat shock 70 kDa protein 1B | P0DMV9 |
| 179 | HMOX1 | heme oxygenase 1 | P09601 |
| 180 | HLA-E | major histocompatibility complex, class I, E | P13747 |
| 181 | HDC | histidine decarboxylase | P19113 |
| 182 | ANGPT2 | angiopoietin 2 | O15123 |
| 183 | GPR17 | G protein-coupled receptor 17 | Q13304 |
| 184 | AMPD1 | adenosine monophosphate deaminase 1 | P23109 |
| 185 | TRDN | triadin | Q13061 |
| 186 | DMD | dystrophin | P11532 |
| 187 | LMNA | lamin A/C | P02545 |
| 188 | GJA1 | gap junction protein alpha 1 | P17302 |
| 189 | BAG3 | BCL2 associated athanogene 3 | O95817 |
| 190 | DSP | desmoplakin | P15924 |
| 191 | TNNI3 | troponin I3, cardiac type | P19429 |
| 192 | HFE | hemochromatosis | Q30201 |
| 193 | TNNT2 | troponin T2, cardiac type | P45379 |
| 194 | MYH6 | myosin heavy chain 6 | P13533 |
| 195 | MYH7 | myosin heavy chain 7 | P12883 |
| 196 | TAZ | tafazzin | Q16635 |
| 197 | COX2 | cytochrome c oxidase subunit II | P00403 |
| 198 | ACTC1 | actin, alpha, cardiac muscle 1 | P68032 |
| 199 | EYA4 | EYA transcriptional coactivator and phosphatase 4 | O95677 |
| 200 | CLIC2 | chloride intracellular channel 2 | O15247 |
| 201 | PSEN1 | presenilin 1 | P49768 |
| 202 | PSEN2 | presenilin 2 | P49810 |
| 203 | FBN1 | fibrillin 1 | P35555 |
| 204 | GLB1 | galactosidase beta 1 | P16278 |
| 205 | PRKAG2 | protein kinase AMP-activated non-catalytic subunit gamma 2 | Q9UGJ0 |
| 206 | TPI1 | triosephosphate isomerase 1 | P60174 |
| 207 | TRIM37 | tripartite motif containing 37 | O94972 |
| 208 | COL1A1 | collagen type I alpha 1 chain | P02452 |
| 209 | TPM1 | tropomyosin 1 (alpha) | P09493 |
| 210 | RET | ret proto-oncogene | P07949 |
| 211 | ENPP1 | ectonucleotide pyrophosphatase/phosphodiesterase 1 | P22413 |
| 212 | PEX7 | peroxisomal biogenesis factor 7 | O00628 |
| 213 | PHYH | phytanoyl-CoA 2-hydroxylase | O14832 |
| 214 | PSMB8 | proteasome subunit beta 8 | P28062 |
| 215 | RPS19 | ribosomal protein S19 | P39019 |
| 216 | ATP5A1 | ATP synthase, H+ transporting, mitochondrial F1 complex, alpha subunit 1, cardiac muscle | P25705 |
| 217 | TMEM70 | transmembrane protein 70 | Q9BUB7 |
| 218 | PRKAR1A | protein kinase cAMP-dependent type I regulatory subunit alpha | P10644 |
| 219 | COL1A2 | collagen type I alpha 2 chain | P08123 |
| 220 | PLOD1 | procollagen-lysine,2-oxoglutarate 5-dioxygenase 1 | Q02809 |
| 221 | ND5 | NADH dehydrogenase, subunit 5 (complex I) | P03915 |
| 222 | ND6 | NADH dehydrogenase, subunit 6 (complex I) | P03923 |
| 223 | WRN | Werner syndrome RecQ like helicase | Q14191 |
| 224 | ALMS1 | ALMS1, centrosome and basal body associated protein | Q8TCU4 |
| 225 | IDS | iduronate 2-sulfatase | P22304 |
| 226 | KIF1B | kinesin family member 1B | O60333 |
| 227 | HBA2 | hemoglobin subunit alpha 2 | P69905 |
| 228 | HADHB | hydroxyacyl-CoA dehydrogenase/3-ketoacyl-CoA thiolase/enoyl-CoA hydratase (trifunctional protein), beta subunit | P55084 |
| 229 | HADHA | hydroxyacyl-CoA dehydrogenase/3-ketoacyl-CoA thiolase/enoyl-CoA hydratase (trifunctional protein), alpha subunit | P40939 |
| 230 | ACAD9 | acyl-CoA dehydrogenase family member 9 | Q9H845 |
| 231 | FXN | frataxin | Q16595 |
| 232 | CAV3 | caveolin 3 | P56539 |
| 233 | GLA | galactosidase alpha | P06280 |
| 234 | RAB3GAP2 | RAB3 GTPase activating non-catalytic protein subunit 2 | Q9H2M9 |
| 235 | VHL | von Hippel-Lindau tumor suppressor | P40337 |
| 236 | ABCC6 | ATP binding cassette subfamily C member 6 | O95255 |
| 237 | ND1 | NADH dehydrogenase, subunit 1 (complex I) | P03886 |
| 238 | CYTB | cytochrome b | P00156 |
| 239 | SDHB | succinate dehydrogenase complex iron sulfur subunit B | P21912 |
| 240 | SDHD | succinate dehydrogenase complex subunit D | O14521 |
| 241 | COX3 | cytochrome c oxidase III | P00414 |
| 242 | SLC17A5 | solute carrier family 17 member 5 | Q9NRA2 |
| 243 | TF | transferrin | P02787 |
| 244 | DTNA | dystrobrevin alpha | Q9Y4J8 |
| 245 | COX1 | cytochrome c oxidase subunit I | P00395 |
| 246 | FLNA | filamin A | P21333 |
| 247 | KCNJ5 | potassium voltage-gated channel subfamily J member 5 | P48544 |
| 248 | JUP | junction plakoglobin | P14923 |
| 249 | ADAMTSL2 | ADAMTS like 2 | Q86TH1 |
| 250 | NOS3 | nitric oxide synthase 3 | P29474 |
| 251 | DNAH8 | dynein axonemal heavy chain 8 | Q96JB1 |
| 252 | PLN | phospholamban | P26678 |
| 253 | SLC3A2 | solute carrier family 3 member 2 | P08195 |
| 254 | ADRA2C | adrenoceptor alpha 2C | P18825 |
| 255 | IL6 | interleukin 6 | P05231 |
| 256 | S100A1 | S100 calcium binding protein A1 | P23297 |
| 257 | NPPB | natriuretic peptide B | P16860 |
| 258 | TTN | titin | Q8WZ42 |
| 259 | NPPA | natriuretic peptide A | P01160 |
| 260 | IGF1 | insulin like growth factor 1 | P05019 |
| 261 | CTGF | connective tissue growth factor | P29279 |
| 262 | CRP | C-reactive protein | P02741 |
| 263 | ATP2A2 | ATPase sarcoplasmic/endoplasmic reticulum Ca2+ transporting 2 | P16615 |
| 264 | IL18 | interleukin 18 | Q14116 |
| 265 | SCN5A | sodium voltage-gated channel alpha subunit 5 | Q14524 |
| 266 | TTR | transthyretin | P02766 |
| 267 | PPARA | peroxisome proliferator activated receptor alpha | Q07869 |
| 268 | EIF3K | eukaryotic translation initiation factor 3 subunit K | Q9UBQ5 |
| 269 | SLC8A1 | solute carrier family 8 member A1 | P32418 |
| 270 | APLNR | apelin receptor | P35414 |
| 271 | DES | desmin | P17661 |
| 272 | CTF1 | cardiotrophin 1 | Q16619 |
| 273 | PPP1R1A | protein phosphatase 1 regulatory inhibitor subunit 1A | Q13522 |
| 274 | CASP3 | caspase 3 | P42574 |
| 275 | CASQ2 | calsequestrin 2 | O14958 |
| 276 | PPARGC1A | PPARG coactivator 1 alpha | Q9UBK2 |
| 277 | SRF | serum response factor | P11831 |
| 278 | KNG1 | kininogen 1 | P01042 |
| 279 | LGALS3 | galectin 3 | P17931 |
| 280 | HIF1A | hypoxia inducible factor 1 alpha subunit | Q16665 |
| 281 | DECR1 | 2,4-dienoyl-CoA reductase 1 | Q16698 |
| 282 | PPARGC1B | PPARG coactivator 1 beta | Q86YN6 |
| 283 | IL6ST | interleukin 6 signal transducer | P40189 |
| 284 | FSD1L | fibronectin type III and SPRY domain containing 1 like | Q9BXM9 |
| 285 | GH1 | growth hormone 1 | P01241 |
| 286 | GRK5 | G protein-coupled receptor kinase 5 | P34947 |
| 287 | AKT1 | AKT serine/threonine kinase 1 | P31749 |
| 288 | LRPPRC | leucine rich pentatricopeptide repeat containing | P42704 |
| 289 | ANXA5 | annexin A5 | P08758 |
| 290 | GATA4 | GATA binding protein 4 | P43694 |
| 291 | FSD1 | fibronectin type III and SPRY domain containing 1 | Q9BTV5 |
| 292 | APRT | adenine phosphoribosyltransferase | P07741 |
| 293 | IL1B | interleukin 1 beta | P01584 |
| 294 | NOS1 | nitric oxide synthase 1 | P29475 |
| 295 | SGCD | sarcoglycan delta | Q92629 |
| 296 | PRKD1 | protein kinase D1 | Q15139 |
| 297 | PRH1 | proline rich protein HaeIII subfamily 1 | P02810 |
| 298 | PTPA | protein phosphatase 2 phosphatase activator | Q15257 |
| 299 | PIK3CA | phosphatidylinositol-4,5-bisphosphate 3-kinase catalytic subunit alpha | P42336 |
| 300 | ANKRD1 | ankyrin repeat domain 1 | Q15327 |
| 301 | ADIPOQ | adiponectin, C1Q and collagen domain containing | Q15848 |
| 302 | MYBPC3 | myosin binding protein C, cardiac | Q14896 |
| 303 | PPARG | peroxisome proliferator activated receptor gamma | P37231 |
| 304 | CD59 | CD59 molecule | P13987 |
| 305 | HP | haptoglobin | P00738 |
| 306 | ATM | ATM serine/threonine kinase | Q13315 |
| 307 | PIK3CD | phosphatidylinositol-4,5-bisphosphate 3-kinase catalytic subunit delta | O00329 |
| 308 | SLC33A1 | solute carrier family 33 member 1 | O00400 |
| 309 | ANGPT1 | angiopoietin 1 | Q15389 |
| 310 | PIK3CG | phosphatidylinositol-4,5-bisphosphate 3-kinase catalytic subunit gamma | P48736 |
| 311 | PDE5A | phosphodiesterase 5A | O76074 |
| 312 | HSPB7 | heat shock protein family B (small) member 7 | Q9UBY9 |
| 313 | NPR2 | natriuretic peptide receptor 2 | P20594 |
| 314 | AVP | arginine vasopressin | P01185 |
| 315 | MEF2A | myocyte enhancer factor 2A | Q02078 |
| 316 | IL4 | interleukin 4 | P05112 |
| 317 | LCN2 | lipocalin 2 | P80188 |
| 318 | PIK3CB | phosphatidylinositol-4,5-bisphosphate 3-kinase catalytic subunit beta | P42338 |
| 319 | RND3 | Rho family GTPase 3 | P61587 |
| 320 | STAT3 | signal transducer and activator of transcription 3 | P40763 |
| 321 | HLA-C | major histocompatibility complex, class I, C | P04222 |
| 322 | HLA-C | HLA class I histocompatibility antigen, Cw-7 alpha chain | P10321 |
| 323 | HLA-C | HLA class I histocompatibility antigen, Cw-1 alpha chain | P30499 |
| 324 | HLA-C | HLA class I histocompatibility antigen, Cw-2 alpha chain | P30501 |
| 325 | HLA-C | HLA class I histocompatibility antigen, Cw-4 alpha chain | P30504 |
| 326 | HLA-C | HLA class I histocompatibility antigen, Cw-8 alpha chain | P30505 |
| 327 | HLA-C | HLA class I histocompatibility antigen, Cw-12 alpha chain | P30508 |
| 328 | HLA-C | HLA class I histocompatibility antigen, Cw-14 alpha chain | P30510 |
| 329 | HLA-C | HLA class I histocompatibility antigen, Cw-15 alpha chain | Q07000 |
| 330 | HLA-C | HLA class I histocompatibility antigen, Cw-18 alpha chain | Q29865 |
| 331 | HLA-C | HLA class I histocompatibility antigen, Cw-16 alpha chain | Q29960 |
| 332 | HLA-C | HLA class I histocompatibility antigen, Cw-6 alpha chain | Q29963 |
| 333 | HLA-C | HLA class I histocompatibility antigen, Cw-17 alpha chain | Q95604 |
| 334 | HLA-C | HLA class I histocompatibility antigen, Cw-5 alpha chain | Q9TNN7 |
| 335 | HSPA4 | heat shock protein family A (Hsp70) member 4 | P34932 |
| 336 | CAD | carbamoyl-phosphate synthetase 2, aspartate transcarbamylase, and dihydroorotase | P27708 |
| 337 | PITX2 | paired like homeodomain 2 | Q99697 |
| 338 | TM7SF2 | transmembrane 7 superfamily member 2 | O76062 |
| 339 | EPO | erythropoietin | P01588 |
| 340 | UTS2 | urotensin 2 | O95399 |
| 341 | IL18BP | interleukin 18 binding protein | O95998 |
| 342 | EDNRA | endothelin receptor type A | P25101 |
| 343 | SEMA6A | semaphorin 6A | Q9H2E6 |
| 344 | ALAS2 | 5'-aminolevulinate synthase 2 | P22557 |
| 345 | XBP1 | X-box binding protein 1 | P17861 |
| 346 | CDK9 | cyclin dependent kinase 9 | P50750 |
| 347 | CHGA | chromogranin A | P10645 |
| 348 | CCR2 | C-C motif chemokine receptor 2 | P41597 |
| 349 | TWIST1 | twist family bHLH transcription factor 1 | Q15672 |
| 350 | MAPK14 | mitogen-activated protein kinase 14 | Q16539 |
| 351 | FSTL3 | follistatin like 3 | O95633 |
| 352 | TRPC6 | transient receptor potential cation channel subfamily C member 6 | Q9Y210 |
| 353 | TRPC3 | transient receptor potential cation channel subfamily C member 3 | Q13507 |
| 354 | XDH | xanthine dehydrogenase | P47989 |
| 355 | CHEK2 | checkpoint kinase 2 | O96017 |
| 356 | CRK | CRK proto-oncogene, adaptor protein | P46108 |
| 357 | ISYNA1 | inositol-3-phosphate synthase 1 | Q9NPH2 |
| 358 | TNC | tenascin C | P24821 |
| 359 | MMRN1 | multimerin 1 | Q13201 |
| 360 | ADIPOR1 | adiponectin receptor 1 | Q96A54 |
| 361 | AIMP2 | aminoacyl tRNA synthetase complex interacting multifunctional protein 2 | Q13155 |
| 362 | TNNI3K | TNNI3 interacting kinase | Q59H18 |
| 363 | DUOX2 | dual oxidase 2 | Q9NRD8 |
| 364 | TXN | thioredoxin | P10599 |
| 365 | PRRT2 | proline rich transmembrane protein 2 | Q7Z6L0 |
| 366 | RYR1 | ryanodine receptor 1 | P21817 |
| 367 | WDTC1 | WD and tetratricopeptide repeats 1 | Q8N5D0 |
| 368 | CSF3 | colony stimulating factor 3 | P09919 |
| 369 | SLC2A1 | solute carrier family 2 member 1 | P11166 |
| 370 | ECE1 | endothelin converting enzyme 1 | P42892 |
| 371 | BRCA1 | BRCA1, DNA repair associated | P38398 |
| 372 | SLC6A2 | solute carrier family 6 member 2 | P23975 |
| 373 | SPP1 | secreted phosphoprotein 1 | P10451 |
| 374 | SOD2 | superoxide dismutase 2 | P04179 |
| 375 | EGFR | epidermal growth factor receptor | P00533 |
| 376 | MME | membrane metalloendopeptidase | P08473 |
| 377 | MMP1 | matrix metallopeptidase 1 | P03956 |
| 378 | SLC7A1 | solute carrier family 7 member 1 | P30825 |
| 379 | MPO | myeloperoxidase | P05164 |
| 380 | CX3CR1 | C-X3-C motif chemokine receptor 1 | P49238 |
| 381 | SDC4 | syndecan 4 | P31431 |
| 382 | LTA | lymphotoxin alpha | P01374 |
| 383 | TNFRSF1B | TNF receptor superfamily member 1B | P20333 |
| 384 | TNFRSF1A | TNF receptor superfamily member 1A | Q13325 |
| 385 | SGCG | sarcoglycan gamma | P63316 |
| 386 | NFKB1 | nuclear factor kappa B subunit 1 | P00981 |
| 387 | PKP2 | plakophilin 2 | P41595 |
| 388 | TFAM | transcription factor A, mitochondrial | [P37088](http://www.uniprot.org/uniprot/P37088||P51168||P51170||P51172) |
| 389 | NCAM1 | neural cell adhesion molecule 1 | P51168 |
| 390 | MYLK | myosin light chain kinase | P51170 |
| 391 | AHSA1 | activator of HSP90 ATPase activity 1 | P51172 |
| 392 | AGTR2 | angiotensin II receptor type 2 | P25100 |
| 393 | GHRL | ghrelin and obestatin prepropeptide | [P78348](http://www.uniprot.org/uniprot/P78348) |
| 394 | POLDIP2 | DNA polymerase delta interacting protein 2 | [Q09045](http://www.uniprot.org/uniprot/Q13639) |
| 395 | MAPK8 | mitogen-activated protein kinase 8 | [Q20387](http://www.uniprot.org/uniprot/Q02297 (20-241)) |
| 396 | GRAP2 | GRB2-related adaptor protein 2 | P08236 |
| 397 | DUOX1 | dual oxidase 1 | T81184 |
| 398 | GPT | glutamic--pyruvic transaminase | P18255 |
| 399 | PPIG | peptidylprolyl isomerase G | P20972 |
| 400 | KLF15 | Kruppel like factor 15 | Q13622 |
| 401 | SETD2 | SET domain containing 2 | P30518 |
| 402 | DNER | delta/notch like EGF repeat containing | P37288 |
| 403 | PKD2 | polycystin 2, transient receptor potential cation channel | P30542 |
| 404 | ANXA2 | annexin A2 | P17342 |
| 405 | ANG | angiogenin | P00915 |
| 406 | ERBIN | erbb2 interacting protein | [P29972](http://www.uniprot.org/uniprot/P29972) |
| 407 | CHDH | choline dehydrogenase | [Q9ULX8](http://www.uniprot.org/uniprot/Q9ULX7) |
| 408 | QRSL1 | glutaminyl-tRNA synthase (glutamine-hydrolyzing)-like 1 | [Q4U2R9](http://www.uniprot.org/uniprot/Q4U2R8) |
| 409 | RNF19A | ring finger protein 19A, RBR E3 ubiquitin protein ligase | [P00918](http://www.uniprot.org/uniprot/P00918) |
| 410 | G6PD | glucose-6-phosphate dehydrogenase | [P07451](http://www.uniprot.org/uniprot/P07451) |
| 411 | CNP | 2',3'-cyclic nucleotide 3' phosphodiesterase | P22748 |
| 412 | AXL | AXL receptor tyrosine kinase | SLC22A7 |
| 413 | SERPINA3 | serpin family A member 3 | P43167 |
| 414 | RNF111 | ring finger protein 111 | O43571 |
| 415 | RBFOX1 | RNA binding protein, fox-1 homolog 1 | P19467 |
| 416 | BIN1 | bridging integrator 1 | P19300 |
| 417 | GNB3 | G protein subunit beta 3 | Q16516 |
| 418 | PTGS2 | prostaglandin-endoperoxide synthase 2 | [P00750](http://www.uniprot.org/uniprot/P00749) |
| 419 | ABCC9 | ATP binding cassette subfamily C member 9 | O15245 |
| 420 | HGF | hepatocyte growth factor | Q9H016 |
| 421 | CAT | catalase | Q9TRC8 |
| 422 | HDAC5 | histone deacetylase 5 | P04035 |
| 423 | LUC7L3 | LUC7 like 3 pre-mRNA splicing factor | P08684 |
| 424 | HOPX | HOP homeobox | P27487 |
| 425 | TLX2 | T-cell leukemia homeobox 2 | P10635 |
| 426 | PINK1 | PTEN induced putative kinase 1 | P11712 |
| 427 | BMP4 | bone morphogenetic protein 4 | P33261 |
| 428 | SLC2A4 | solute carrier family 2 member 4 | P20815 |
| 429 | RHOU | ras homolog family member U | P24462 |
| 430 | CXCL16 | C-X-C motif chemokine ligand 16 | P08183 |
| 431 | WNK1 | WNK lysine deficient protein kinase 1 | P46721 |
| 432 | LOC107984055 | medium-wave-sensitive opsin 1 | Q9Y6L7 |
| 433 | API5 | Apoptosis inhibitor 5 | O15441 |
| 434 | MPPED1 | Metallophosphoesterase domain-containing protein 1 | O15442 |
| 435 | MOK | MOK protein kinase | P33527 |
| 436 | PBK | PDZ binding kinase | P20813 |
| 437 | GIGYF1 | GRB10 interacting GYF protein 1 | P10632 |
| 438 | GORASP1 | golgi reassembly stacking protein 1 | P35869 |
| 439 | CDK15 | cyclin dependent kinase 15 | O94957 |
| 440 | PURB | purine rich element binding protein B | Q9NPD6 |
| 441 | RELA | RELA proto-oncogene, NF-kB subunit | P22309 |
| 442 | CISH | cytokine inducible SH2 containing protein | P35503 |
| 443 | ZKSCAN7 | zinc finger with KRAB and SCAN domains 7 | P16662 |
| 444 | RAC2 | ras-related C3 botulinum toxin substrate 2 (rho family, small GTP binding protein Rac2) | Q92888 |
| 445 | CMA1 | chymase 1 | O95343 |
| 446 | KDM5B | lysine demethylase 5B | P02768 |
| 447 | CCR7 | C-C motif chemokine receptor 7 | P08253 |
| 448 | NES | nestin | P14780 |
| 449 | TRPV6 | transient receptor potential cation channel subfamily V member 6 | P46059 |
| 450 | BMPR1A | bone morphogenetic protein receptor type 1A | P09960 |
| 451 | SLC18A3 | solute carrier family 18 member A3 | P46663 |
| 452 | MGEA5 | meningioma expressed antigen 5 (hyaluronidase) | Q16349 |
| 453 | MYL7 | myosin light chain 7 | P30556 |
| 454 | RBM25 | RNA binding motif protein 25 | P05023 |
| 455 | SYBU | syntabulin | P09210 |
| 456 | RAB1A | RAB1A, member RAS oncogene family | Q9UJU3 |
| 457 | PRKCA | protein kinase C alpha | P09212 |
| 458 | ENAH | enabled homolog (Drosophila) | O76083 |
| 459 | TPH2 | tryptophan hydroxylase 2 | [Q8TCC8](http://www.uniprot.org/uniprot/Q8TCC7) |
| 460 | SLC6A4 | solute carrier family 6 member 4 | Q92960 |
| 461 | NCF1 | neutrophil cytosolic factor 1 | Q9NSA1 |
| 462 | SLC6A8 | solute carrier family 6 member 8 | P57825 |
| 463 | PRKACG | protein kinase cAMP-activated catalytic subunit gamma | P60633 |
| 464 | SLN | sarcolipin | Q12792 |
| 465 | CFAP97 | cilia and flagella associated protein 97 | P08588 |
| 466 | PTEN | phosphatase and tensin homolog | P07550 |
| 467 | BAD | BCL2 associated agonist of cell death | P27986 |
| 468 | SCD | stearoyl-CoA desaturase | P13945 |
| 469 | FSTL1 | follistatin like 1 | P28482 |
| 470 | HCN2 | hyperpolarization activated cyclic nucleotide gated potassium channel 2 | O00460 |
| 471 | CCL19 | C-C motif chemokine ligand 19 | Q92570 |
| 472 | ROCK1 | Rho associated coiled-coil containing protein kinase 1 | P04799 |
| 473 | CHPT1 | choline phosphotransferase 1 | Q4ZHU7 |
| 474 | SLC24A3 | solute carrier family 24 member 3 | P00442 |
| 475 | ATXN1 | ataxin 1 | Q14433 |
| 476 | S100B | S100 calcium binding protein B | P05884 |
| 477 | ATXN7 | ataxin 7 | P16859 |
| 478 | NLN | neurolysin | Q03406 |
| 479 | RYR3 | ryanodine receptor 3 | P02671 |
| 480 | BDNF | brain derived neurotrophic factor | P00747 |
| 481 | RPL32 | ribosomal protein L32 | P05121 |
| 482 | MAP4K5 | mitogen-activated protein kinase kinase kinase kinase 5 | P10275 |
| 483 | ADD1 | adducin 1 | P06401 |
| 484 | RETN | resistin | P04150 |
| 485 | CLCN3 | chloride voltage-gated channel 3 | P19099 |
| 486 | PRDM16 | PR/SET domain 16 | P18405 |
| 487 | ECD | ecdysoneless cell cycle regulator | P31213 |
| 488 | MAP2K3 | mitogen-activated protein kinase kinase 3 | Q9H8P1 |
| 489 | SELP | selectin P | P04279 |
| 490 | LILRB1 | leukocyte immunoglobulin like receptor B1 | Q06433 |
| 491 | RENBP | renin binding protein | P54290 |
| 492 | MAPK9 | mitogen-activated protein kinase 9 | Q9NY48 |
| 493 | SGK1 | serum/glucocorticoid regulated kinase 1 | Q8IZS9 |
| 494 | SUGT1 | SGT1 homolog, MIS12 kinetochore complex assembly cochaperone | Q10600 |
| 495 | PTX3 | pentraxin 3 | Q22868 |
| 496 | PRL | prolactin | O60841 |
| 497 | CLCNKA | chloride voltage-gated channel Ka | Q13699 |
| 498 | SAV1 | salvador family WW domain containing protein 1 | Q6TME5 |
| 499 | CCL21 | C-C motif chemokine ligand 21 | Q13937 |
| 500 | RGS4 | regulator of G-protein signaling 4 | Q19585 |
| 501 | CX3CL1 | C-X3-C motif chemokine ligand 1 | P54285 |
| 502 | PTN | pleiotrophin | O00306 |
| 503 | SDC2 | syndecan 2 | Q00976 |
| 504 | SDS | serine dehydratase | O00555 |
| 505 | SGTA | small glutamine rich tetratricopeptide repeat containing alpha | O43497 |
| 506 | NOX5 | NADPH oxidase 5 | O95180 |
| 507 | SESN2 | sestrin 2 | Q9P0X5 |
| 508 | DNM1L | dynamin 1 like | O75470 |
| 509 | TCHP | trichoplein keratin filament binding | P12865 |
| 510 | SPZ1 | spermatogenic leucine zipper 1 | P30907 |
| 511 | IKBKG | inhibitor of nuclear factor kappa B kinase subunit gamma | Q15244 |
| 512 | DENR | density regulated re-initiation and release factor | Q15245 |
| 513 | DGAT1 | diacylglycerol O-acyltransferase 1 | Q15439 |
| 514 | CBR3 | carbonyl reductase 3 | P05305 |
| 515 | CDS2 | CDP-diacylglycerol synthase 2 | P01375 |
| 516 | CCK | cholecystokinin | P00797 |
| 517 | CASP1 | caspase 1 | O14794 |
| 518 | CAST | calpastatin | Q92737 |
| 519 | ARID1A | AT-rich interaction domain 1A | Q9BYF2 |
| 520 | CALCR | calcitonin receptor | P35228 |
| 521 | NUP85 | nucleoporin 85 | P15692 |
| 522 | ZC3H12A | zinc finger CCCH-type containing 12A | P61073 |
| 523 | CEP290 | centrosomal protein 290 | P62380 |
| 524 | CH17-360D5.1 | neuropeptide Y receptor type 4-like | P11086 |
| 525 | ACTN4 | actinin alpha 4 | P23582 |
| 526 | CALR | calreticulin | O75053 |
| 527 | TCL1A | T-cell leukemia/lymphoma 1A | Q02818 |
| 528 | MAP3K14 | mitogen-activated protein kinase kinase kinase 14 | P34466 |
| 529 | UCN2 | urocortin 2 | P77503 |
| 530 | MLIP | muscular LMNA interacting protein | O14757 |
| 531 | GDF15 | growth differentiation factor 15 | P13501 |
| 532 | NR2E3 | nuclear receptor subfamily 2 group E member 3 | Q86V25 |
| 533 | CD44 | CD44 molecule (Indian blood group) | Q7Z4H5 |
| 534 | ISG15 | ISG15 ubiquitin-like modifier | Q9UID4 |
| 535 | CD68 | CD68 molecule | P119679 |
| 536 | CD69 | CD69 molecule | P177200 |
| 537 | CD70 | CD70 molecule | Q9H612 |
| 538 | HDAC4 | histone deacetylase 4 | Q15555 |
| 539 | MLEC | malectin | P49006 |
| 540 | TLK1 | tousled like kinase 1 | P50461 |
| 541 | SCARB2 | scavenger receptor class B member 2 | P48061 |
| 542 | CD36 | CD36 molecule | P24530 |
| 543 | HAND2 | heart and neural crest derivatives expressed 2 | P00813 |
| 544 | NEXN | nexilin F-actin binding protein | P01019 |
| 545 | MAP3K13 | mitogen-activated protein kinase kinase kinase 13 | P25098 |
| 546 | MSC | musculin | P43235 |
| 547 | MAPKAPK2 | mitogen-activated protein kinase-activated protein kinase 2 | Q8TF48 |
| 548 | LONP1 | lon peptidase 1, mitochondrial | P29275 |
| 549 | MYOCD | myocardin | P29274 |
| 550 | HAND1 | heart and neural crest derivatives expressed 1 | P35318 |
| 551 | OPN4 | opsin 4 | P81605 |
| 552 | ABCG2 | ATP binding cassette subfamily G member 2 (Junior blood group) | Q9Y280 |
| 553 | MED23 | mediator complex subunit 23 | P04627 |
| 554 | MFN2 | mitofusin 2 | Q96EB7 |
| 555 | BMPR2 | bone morphogenetic protein receptor type 2 | P01286 |
| 556 | STIM1 | stromal interaction molecule 1 | P28300 |
| 557 | ABCC8 | ATP binding cassette subfamily C member 8 | P15382 |
| 558 | SYT1 | synaptotagmin 1 | P01579 |
| 559 | ADAM17 | ADAM metallopeptidase domain 17 | P60321 |
| 560 | SLU7 | SLU7 homolog, splicing factor | P0DMV10 |
| 561 | TFR2 | transferrin receptor 2 | P0DMV11 |
| 562 | TFRC | transferrin receptor | P09601 |
| 563 | TGFB1 | transforming growth factor beta 1 | P13747 |
| 564 | THBS1 | thrombospondin 1 | P19113 |
| 565 | THBS2 | thrombospondin 2 | O15124 |
| 566 | TIMP1 | TIMP metallopeptidase inhibitor 1 | Q13305 |
| 567 | STC1 | stanniocalcin 1 | P23110 |
| 568 | STAT4 | signal transducer and activator of transcription 4 | Q13062 |
| 569 | POSTN | periostin | P11532 |
| 570 | SUMO3 | small ubiquitin-like modifier 3 | P02545 |
| 571 | SUMO2 | small ubiquitin-like modifier 2 | P17302 |
| 572 | SNAP25 | synaptosome associated protein 25 | O95818 |
| 573 | FSCN1 | fascin actin-bundling protein 1 | P22934 |
| 574 | BNIP3 | BCL2 interacting protein 3 | P26439 |
| 575 | SOAT1 | sterol O-acyltransferase 1 | Q30202 |
| 576 | SOD3 | superoxide dismutase 3 | P45379 |
| 577 | BNIP3L | BCL2 interacting protein 3 like | P13533 |
| 578 | SOX3 | SRY-box 3 | P12883 |
| 579 | SPTBN1 | spectrin beta, non-erythrocytic 1 | Q16636 |
| 580 | SRI | sorcin | P135661 |
| 581 | CORIN | corin, serine peptidase | P203290 |
| 582 | TIMP3 | TIMP metallopeptidase inhibitor 3 | O65183 |
| 583 | TLR4 | toll like receptor 4 | O145613 |
| 584 | SEMA4D | semaphorin 4D | P49768 |
| 585 | VEGFB | vascular endothelial growth factor B | P49810 |
| 586 | AK6 | adenylate kinase 6 | P35555 |
| 587 | BEST1 | bestrophin 1 | P16278 |
| 588 | ALYREF | Aly/REF export factor | Q9UGJ1 |
| 589 | YY1 | YY1 transcription factor | P60175 |
| 590 | ZBTB17 | zinc finger and BTB domain containing 17 | O94973 |
| 591 | MAP3K12 | mitogen-activated protein kinase kinase kinase 12 | P02452 |
| 592 | DDR1 | discoidin domain receptor tyrosine kinase 1 | P09493 |
| 593 | SCG2 | secretogranin II | P07949 |
| 594 | MANF | mesencephalic astrocyte derived neurotrophic factor | P22413 |
| 595 | NAMPT | nicotinamide phosphoribosyltransferase | O29036 |
| 596 | FKRP | fukutin related protein | O43240 |
| 597 | VDR | vitamin D (1,25- dihydroxyvitamin D3) receptor | P28062 |
| 598 | UTRN | utrophin | P39019 |
| 599 | SLC9A6 | solute carrier family 9 member A6 | P25705 |
| 600 | FST | follistatin | Q9BUB8 |
| 601 | PRMT5 | protein arginine methyltransferase 5 | P05602 |
| 602 | TOP2B | topoisomerase (DNA) II beta | P03081 |
| 603 | CDS1 | CDP-diacylglycerol synthase 1 | Q02810 |
| 604 | RACK1 | receptor for activated C kinase 1 | P03931 |
| 605 | CRISP2 | cysteine rich secretory protein 2 | P03939 |
| 606 | TRAF3 | TNF receptor associated factor 3 | Q14192 |
| 607 | TRPC1 | transient receptor potential cation channel subfamily C member 1 | Q8TCU5 |
| 608 | CDKN1A | cyclin dependent kinase inhibitor 1A | P22305 |
| 609 | OPN1MW2 | opsin 1, medium wave sensitive 2 | O60334 |
| 610 | SUMO1 | small ubiquitin-like modifier 1 | P69905 |
| 611 | HTR2A | 5-hydroxytryptamine receptor 2A | P55084 |
| 612 | F2RL1 | F2R like trypsin receptor 1 | P40939 |
| 613 | IL7 | interleukin 7 | Q9H846 |
| 614 | IL9 | interleukin 9 | Q16596 |
| 615 | IL10 | interleukin 10 | P43979 |
| 616 | IL16 | interleukin 16 | P94238 |
| 617 | ILF3 | interleukin enhancer binding factor 3 | Q9H2M10 |
| 618 | ILK | integrin linked kinase | P40338 |
| 619 | IRF1 | interferon regulatory factor 1 | O95256 |
| 620 | ISG20 | interferon stimulated exonuclease gene 20 | P03886 |
| 621 | ISL1 | ISL LIM homeobox 1 | P00156 |
| 622 | ITPR1 | inositol 1,4,5-trisphosphate receptor type 1 | P21912 |
| 623 | ITPR2 | inositol 1,4,5-trisphosphate receptor type 2 | O14522 |
| 624 | IL5 | interleukin 5 | P00415 |
| 625 | PTK2B | protein tyrosine kinase 2 beta | Q9NRA3 |
| 626 | FASLG | Fas ligand | P02788 |
| 627 | ID2 | inhibitor of DNA binding 2, HLH protein | Q9Y4J9 |
| 628 | IFNA2 | interferon alpha 2 | P00395 |
| 629 | ICK | intestinal cell kinase | P21333 |
| 630 | APOE | apolipoprotein E | P48544 |
| 631 | IGF2 | insulin like growth factor 2 | P14923 |
| 632 | FGF4 | fibroblast growth factor 4 | Q86TH2 |
| 633 | FDPS | farnesyl diphosphate synthase | P29475 |
| 634 | IL1A | interleukin 1 alpha | Q96JB2 |
| 635 | TPCN2 | two pore segment channel 2 | P26678 |
| 636 | IL2RA | interleukin 2 receptor subunit alpha | P08195 |
| 637 | JARID2 | jumonji and AT-rich interaction domain containing 2 | P18825 |
| 638 | ESRRA | estrogen related receptor alpha | P05231 |
| 639 | KCNA3 | potassium voltage-gated channel subfamily A member 3 | P23297 |
| 640 | LY75 | lymphocyte antigen 75 | P16860 |
| 641 | CTTN | cortactin | Q8WZ43 |
| 642 | LUM | lumican | P01160 |
| 643 | LPA | lipoprotein(a) | P05019 |
| 644 | PRSS55 | protease, serine 55 | P29279 |
| 645 | KCNH2 | potassium voltage-gated channel subfamily H member 2 | P02741 |
| 646 | ESR2 | estrogen receptor 2 | P16615 |
| 647 | KCNQ1 | potassium voltage-gated channel subfamily Q member 1 | Q14932 |
| 648 | ESR1 | estrogen receptor 1 | Q15340 |
| 649 | ARG1 | arginase 1 | P02767 |
| 650 | RHOA | ras homolog family member A | Q07870 |
| 651 | ERN1 | endoplasmic reticulum to nucleus signaling 1 | Q9UBQ6 |
| 652 | LEP | leptin | P32418 |
| 653 | EPHX2 | epoxide hydrolase 2 | P35414 |
| 654 | LIFR | leukemia inhibitory factor receptor alpha | P17661 |
| 655 | EPHA3 | EPH receptor A3 | Q10425 |
| 656 | APOA1 | apolipoprotein A1 | Q7328 |
| 657 | GNAQ | G protein subunit alpha q | P42575 |
| 658 | GOLGB1 | golgin B1 | O14959 |
| 659 | RBM20 | RNA binding motif protein 20 | Q9UBK3 |
| 660 | ABCA4 | ATP binding cassette subfamily A member 4 | P11831 |
| 661 | BRD1 | bromodomain containing 1 | P01042 |
| 662 | DAPK2 | death associated protein kinase 2 | P17931 |
| 663 | ANK2 | ankyrin 2 | Q16731 |
| 664 | GIT1 | GIT ArfGAP 1 | Q16764 |
| 665 | SLC27A6 | solute carrier family 27 member 6 | Q86YN7 |
| 666 | NNT | nicotinamide nucleotide transhydrogenase | P40190 |
| 667 | TBK1 | TANK binding kinase 1 | Q9BXM10 |
| 668 | ALOX15 | arachidonate 15-lipoxygenase | P01241 |
| 669 | MTOR | mechanistic target of rapamycin | P34947 |
| 670 | VPS4A | vacuolar protein sorting 4 homolog A | P31749 |
| 671 | GATM | glycine amidinotransferase | P42704 |
| 672 | GC | GC, vitamin D binding protein | P08758 |
| 673 | GAPDH | glyceraldehyde-3-phosphate dehydrogenase | P43694 |
| 674 | GABPA | GA binding protein transcription factor alpha subunit | Q9BTV6 |
| 675 | GHSR | growth hormone secretagogue receptor | P07741 |
| 676 | GIF | gastric intrinsic factor | P01584 |
| 677 | GABBR1 | gamma-aminobutyric acid type B receptor subunit 1 | P29475 |
| 678 | GAB1 | GRB2 associated binding protein 1 | Q62351 |
| 679 | XRCC6 | X-ray repair cross complementing 6 | Q139841 |
| 680 | FRZB | frizzled-related protein | P02811 |
| 681 | LGALS13 | galectin 13 | Q15258 |
| 682 | GSN | gelsolin | P42337 |
| 683 | GSR | glutathione-disulfide reductase | Q15327 |
| 684 | HK2 | hexokinase 2 | Q15848 |
| 685 | FOXM1 | forkhead box M1 | Q14896 |
| 686 | HMGB1 | high mobility group box 1 | P37231 |
| 687 | PDS5B | PDS5 cohesin associated factor B | P13987 |
| 688 | NR4A1 | nuclear receptor subfamily 4 group A member 1 | P00738 |
| 689 | HNRNPD | heterogeneous nuclear ribonucleoprotein D | Q13316 |
| 690 | HOXD10 | homeobox D10 | O00471 |
| 691 | HRC | histidine rich calcium binding protein | O00542 |
| 692 | HSPA2 | heat shock protein family A (Hsp70) member 2 | Q15390 |
| 693 | FOXC1 | forkhead box C1 | P48737 |
| 694 | XIAP | X-linked inhibitor of apoptosis | O76075 |
| 695 | AKR1B1 | aldo-keto reductase family 1 member B | Q9UBY10 |
| 696 | ANXA6 | annexin A6 | P18224 |
| 697 | EHD3 | EH domain containing 3 | P37633 |
| 698 | GNL2 | G protein nucleolar 2 | Q02079 |
| 699 | RHOD | ras homolog family member D | P05112 |
| 700 | ZFPM2 | zinc finger protein, FOG family member 2 | P80188 |
| 701 | RPGRIP1L | RPGRIP1 like | P42338 |
| 702 | ATP11A | ATPase phospholipid transporting 11A | P61587 |
| 703 | PDS5A | PDS5 cohesin associated factor A | P40763 |
| 704 | HCRTR2 | hypocretin receptor 2 | P04222 |
| 705 | HTT | huntingtin | P10321 |
| 706 | ARC | activity regulated cytoskeleton associated protein | P30499 |
| 707 | GPD1L | glycerol-3-phosphate dehydrogenase 1-like | P30501 |
| 708 | KCNIP2 | potassium voltage-gated channel interacting protein 2 | P30504 |
| 709 | FRMD4B | FERM domain containing 4B | P30505 |
| 710 | HSPG2 | heparan sulfate proteoglycan 2 | P30508 |
| 711 | ATP2B4 | ATPase plasma membrane Ca2+ transporting 4 | P30510 |
| 712 | GP6 | glycoprotein VI platelet | Q07000 |
| 713 | CDK16 | cyclin dependent kinase 16 | Q29865 |
| 714 | PDC | phosducin | Q29960 |
| 715 | CRMP1 | collapsin response mediator protein 1 | Q29963 |
| 716 | PDE9A | phosphodiesterase 9A | Q95604 |
| 717 | GDE1 | glycerophosphodiester phosphodiesterase 1 | Q9TNN8 |
| 718 | PDK1 | pyruvate dehydrogenase kinase 1 | P20484 |
| 719 | PDK4 | pyruvate dehydrogenase kinase 4 | P13260 |
| 720 | PECAM1 | platelet and endothelial cell adhesion molecule 1 | Q99698 |
| 721 | RTEL1 | regulator of telomere elongation helicase 1 | O76063 |
| 722 | MAP3K20 | mitogen-activated protein kinase kinase kinase 20 | P01589 |
| 723 | CRAT | carnitine O-acetyltransferase | O96597 |
| 724 | RMDN1 | regulator of microtubule dynamics 1 | O97196 |
| 725 | FOXP3 | forkhead box P3 | P25102 |
| 726 | NOX4 | NADPH oxidase 4 | Q9H2E7 |
| 727 | TRIM72 | tripartite motif containing 72 | P22557 |
| 728 | CRYZ | crystallin zeta | P17861 |
| 729 | OGN | osteoglycin | P50750 |
| 730 | PARP1 | poly(ADP-ribose) polymerase 1 | P10645 |
| 731 | OPRM1 | opioid receptor mu 1 | P41597 |
| 732 | P2RX1 | purinergic receptor P2X 1 | Q17406 |
| 733 | P2RX7 | purinergic receptor P2X 7 | Q18273 |
| 734 | P2RY2 | purinergic receptor P2Y2 | O95634 |
| 735 | P2RY4 | pyrimidinergic receptor P2Y4 | Q9Y211 |
| 736 | P2RY6 | pyrimidinergic receptor P2Y6 | Q13508 |
| 737 | P4HB | prolyl 4-hydroxylase subunit beta | P47990 |
| 738 | PEBP1 | phosphatidylethanolamine binding protein 1 | O96018 |
| 739 | PGK1 | phosphoglycerate kinase 1 | P46109 |
| 740 | CPT2 | carnitine palmitoyltransferase 2 | Q9NPH3 |
| 741 | CPT1A | carnitine palmitoyltransferase 1A | P24822 |
| 742 | XIRP2 | xin actin binding repeat containing 2 | Q13202 |
| 743 | PPARD | peroxisome proliferator activated receptor delta | Q96A55 |
| 744 | COL3A1 | collagen type III alpha 1 chain | Q13156 |
| 745 | TRPM4 | transient receptor potential cation channel subfamily M member 4 | Q59H19 |
| 746 | CNR2 | cannabinoid receptor 2 | Q9NRD9 |
| 747 | SLC52A1 | solute carrier family 52 member 1 | P10600 |
| 748 | CNN1 | calponin 1 | Q7Z6L1 |
| 749 | RMDN3 | regulator of microtubule dynamics 3 | P21818 |
| 750 | PPP2R1A | protein phosphatase 2 scaffold subunit Aalpha | Q8N5D1 |
| 751 | PPP3CA | protein phosphatase 3 catalytic subunit alpha | P09919 |
| 752 | PPA1 | pyrophosphatase (inorganic) 1 | P11166 |
| 753 | POLR2A | RNA polymerase II subunit A | P42892 |
| 754 | KLF14 | Kruppel like factor 14 | P38398 |
| 755 | PIM1 | Pim-1 proto-oncogene, serine/threonine kinase | P23975 |
| 756 | CP | ceruloplasmin | P10451 |
| 757 | PIN1 | peptidylprolyl cis/trans isomerase, NIMA-interacting 1 | P04179 |
| 758 | IGFBP4 | insulin like growth factor binding protein 4 | P00533 |
| 759 | PKD1 | polycystin 1, transient receptor potential channel interacting | P08473 |
| 760 | PKHD1 | polycystic kidney and hepatic disease 1 (autosomal recessive) | P03956 |
| 761 | NFATC4 | nuclear factor of activated T-cells 4 | P30825 |
| 762 | PLAT | plasminogen activator, tissue type | P05164 |
| 763 | TPCN1 | two pore segment channel 1 | P49238 |
| 764 | COL11A2 | collagen type XI alpha 2 chain | P31431 |
| 765 | NRF1 | nuclear respiratory factor 1 | P01374 |
| 766 | DHRS7C | dehydrogenase/reductase 7C | P20333 |
| 767 | MIP | major intrinsic protein of lens fiber | Q13326 |
| 768 | MITF | melanogenesis associated transcription factor | P63316 |
| 769 | MMP3 | matrix metallopeptidase 3 | P00981 |
| 770 | MMP12 | matrix metallopeptidase 12 | P41595 |
| 771 | S1PR1 | sphingosine-1-phosphate receptor 1 | [P37088](http://www.uniprot.org/uniprot/P37088||P51168||P51170||P51172) |
| 772 | NR0B1 | nuclear receptor subfamily 0 group B member 1 | P51168 |
| 773 | DPYS | dihydropyrimidinase | P51170 |
| 774 | DPYD | dihydropyrimidine dehydrogenase | P51172 |
| 775 | MEF2C | myocyte enhancer factor 2C | P25100 |
| 776 | EGR1 | early growth response 1 | [P78348](http://www.uniprot.org/uniprot/P78348) |
| 777 | ELN | elastin | [Q31729](http://www.uniprot.org/uniprot/Q13639) |
| 778 | ELANE | elastase, neutrophil expressed | [Q43071](http://www.uniprot.org/uniprot/Q02297 (20-241)) |
| 779 | ARRB1 | arrestin beta 1 | P08237 |
| 780 | SMAD1 | SMAD family member 1 | T81185 |
| 781 | ARRB2 | arrestin beta 2 | P23689 |
| 782 | MAS1 | MAS1 proto-oncogene, G protein-coupled receptor | P26406 |
| 783 | DNM2 | dynamin 2 | Q13623 |
| 784 | DNASE1 | deoxyribonuclease 1 | P30518 |
| 785 | AGER | advanced glycosylation end-product specific receptor | P37288 |
| 786 | NFE2L1 | nuclear factor, erythroid 2 like 1 | P30542 |
| 787 | NFE2L2 | nuclear factor, erythroid 2 like 2 | P17342 |
| 788 | NGF | nerve growth factor | P00915 |
| 789 | CTSB | cathepsin B | [P29972](http://www.uniprot.org/uniprot/P29972) |
| 790 | CTRL | chymotrypsin like | [Q9ULX9](http://www.uniprot.org/uniprot/Q9ULX7) |
| 791 | LGALS16 | galectin 16 | [Q4U2R10](http://www.uniprot.org/uniprot/Q4U2R8) |
| 792 | CST3 | cystatin C | [P00918](http://www.uniprot.org/uniprot/P00918) |
| 793 | CNOT3 | CCR4-NOT transcription complex subunit 3 | [P07451](http://www.uniprot.org/uniprot/P07451) |
| 794 | NPY | neuropeptide Y | P22748 |
| 795 | NOTCH3 | notch 3 | SLC22A8 |
| 796 | CSF2 | colony stimulating factor 2 | P43168 |
| 797 | CSF1 | colony stimulating factor 1 | O43572 |
| 798 | NFATC2 | nuclear factor of activated T-cells 2 | P19133 |
| 799 | NDUFS6 | NADH:ubiquinone oxidoreductase subunit S6 | P18966 |
| 800 | RMDN2 | regulator of microtubule dynamics 2 | Q16517 |
| 801 | NQO1 | NAD(P)H quinone dehydrogenase 1 | [P00751](http://www.uniprot.org/uniprot/P00749) |
| 802 | DDIT3 | DNA damage inducible transcript 3 | O15246 |
| 803 | DCN | decorin | Q9H017 |
| 804 | DBH | dopamine beta-hydroxylase | Q9TRC9 |
| 805 | DAG1 | dystroglycan 1 | P04035 |
| 806 | CYP27B1 | cytochrome P450 family 27 subfamily B member 1 | P08684 |
| 807 | CYP2J2 | cytochrome P450 family 2 subfamily J member 2 | P27487 |
| 808 | CYP2E1 | cytochrome P450 family 2 subfamily E member 1 | P10635 |
| 809 | GRK3 | G protein-coupled receptor kinase 3 | P11712 |
| 810 | CYBB | cytochrome b-245 beta chain | P33261 |
| 811 | PPP1R12A | protein phosphatase 1 regulatory subunit 12A | P20815 |
| 812 | ATP2A3 | ATPase sarcoplasmic/endoplasmic reticulum Ca2+ transporting 3 | P24462 |
