# Supplementary material for: Integrating Pharmacokinetics Study, Network Analysis, and Experimental Validation to Uncover the Mechanism of Qiliqiangxin Capsule Against Chronic Heart Failure
Source: Front Pharmacol. 2019 Sep 18;10:1046. doi: 10.3389/fphar.2019.01046 (PMC6759796; doi:10.3389/fphar.2019.01046)
Supplement: Supplementary file 3 [file Table_3.doc]

**SUPPLEMENTARY TABLE S3 ׀** The top 10 representative biological processes among 120 candidate targets.

| **No.** | **Function** | **Groups** | **Group genes** |
| --- | --- | --- | --- |
| 1 | Blood circulation | Group25 | ABCB1,ABCC2,ACE,ACE2,ADORA1,ADORA2A,ADORA2B,ADRA1D,ADRA2C,ADRB1,ADRB2,ADRB3,AKR1B1,AKT1,ALB,ALOX15,AQP1,ATP1A1,ATP5A1,AVPR2,CA2,CA7,CACNA2D1,COX2,CYP11B1,CYP11B2,CYP1A1,CYP3A4,DBH,DNM2,ECE1,EGFR,ERBB2,ESR1,HSPA2,KCNH2,KCNMA1,LGALS3,MME,MMP1,MMP12,MMP2,MMP3,MMP9,NOS2,NOX4,P2RX1,PIK3CG,PIK3R1,PPARA,PPARD,PPARG,PTGS1,PTGS2,PTK2B,SCN5A,SLC6A4,SOAT1,SRD5A2,STAT3,TNFRSF1A,TTR,UGT1A1,VEGFA |
| 2 | Response to oxidative stress | Group24 | ABCC2,ACE,ACE2,ADORA1,ADORA2A,ADORA2B,ADRA1D,ADRA2C,ADRB1,ADRB2,ADRB3,AHR,AKR1B1,AKT1,ALB,ALOX15,AQP1,ATP1A1,ATP5A1,AVPR2,AXL,CA2,CA3,COX1,COX2,CYP11B1,CYP11B2,CYP1A1,CYP1A2,DBH,DNM2,EGFR,ERBB2,ERN1,ESR1,ESR2,ESRRA,KCNMA1,LCN2,MME,MMP1,MMP12,MMP2,MMP3,MMP9,MPO,ND1,NFE2L2,NOS2,NOX4,NQO1,NR1I2,NR4A1,P2RX1,P4HB,PIK3R1,PIM1,PPARA,PPARD,PPARG,PTGS1,PTGS2,PTK2B,S100B,SCN5A,SLC22A2,SLC22A8,SLC6A2,SLC6A4,SOAT1,SRD5A2,STAT3,TNFRSF1A,UGT1A1,VEGFA,XDH |
| 3 | Muscle contraction | Group23 | ABCB1,ACE2,ADORA1,ADORA2A,ADORA2B,ADRA1D,ADRA2C,ADRB1,ADRB2,ADRB3,AKR1B1,AQP1,ATP1A1,AVPR2,CA2,CA7,CACNA2D1,CYP11B2,EGFR,KCNH2,KCNMA1,NOS2,P2RX1,PIK3CG,PPARD,PTGS2,PTK2B,S100B,SCN5A,SLC22A6 |
| 4 | Positive regulation of blood circulation | Group22 | ABCC2,ACE2,ADORA1,ADORA2A,ADORA2B,ADRA1D,ADRA2C,ADRB1,ADRB2,AKR1B1,AKT1,ALB,AQP1,ATP1A1,AVPR2,AXL,CYP1A2,DBH,DNM2,EGFR,ERN1,ESR1,LCN2,MME,MMP12,MMP2,MMP9,MPO,NFE2L2,NOS2,NOX4,NQO1,NR4A1,PIK3CG,PIK3R1,PIM1,PPARA,PPARG,PTGS2,PTK2B,SERPINE1,SLC6A2,SLC6A4,STAT3,TNFRSF1A,UGT1A1,VEGFA |
| 5 | Drug metabolic process | Group21 | ABCB11,ABCC1,ABCC2,AHR,AKR1B1,AKT1,ALOX15,AQP1,ATP1A1,CBR3,COX1,CYP11B1,CYP11B2,CYP1A1,CYP1A2,CYP2B6,CYP2C8,CYP2C9,CYP2D6,CYP3A4,CYP3A5,CYP3A7,EGFR,ESR1,KCNH2,NQO1,NR1I2,PPARA,PPARD,PPARG,PTGS1,PTGS2,SLC6A2,SOAT1,SRD5A2,TNFRSF1A,TTR,UGT1A1 |
| 6 | Response to inorganic substance | Group20 | ABCC2,ACE2,AKR1B1,AKT1,ALOX15,AQP1,AXL,CA2,CA3,COX1,COX3,CYP11B1,CYP11B2,CYP1A1,CYP1A2,CYP2B6,CYP2C8,CYP2C9,CYTB,DNM2,EGFR,ESR1,KCNMA1,LCN2,MMP3,MMP9,MPO,ND1,NFE2L2,NOS2,NOX4,NQO1,P4HB,PTGS1,PTGS2,PTK2B,SCN5A,SDHB,SDHD,SLC6A2,STAT3,XDH |
| 7 | Regulation of blood pressure | Group19 | ABCB11,ABCC2,ACE,ACE2,ADORA1,ADRA1D,ADRA2C,ADRB1,ADRB2,ADRB3,AKR1B1,AKT1,ATP1A1,AVPR2,CYP11B1,CYP11B2,CYP1A1,CYP1A2,CYP2B6,CYP2C8,CYP2C9,CYP2D6,CYP3A4,CYP3A5,CYP3A7,ECE1,EGFR,ESR1,KCNH2,MME,MMP1,MMP12,MMP2,MMP3,MMP9,NOS2,NR1I2,P2RX1,PPARA,PPARD,PPARG,PTGS1,PTGS2,SLCO1B1,SOAT1,SRD5A2,TTR,UGT1A1 |
| 8 | Regulation of systemic arterial blood pressure | Group18 | ACE,ACE2,ADORA1,ADORA2A,ADORA2B,ADRA1D,ADRA2C,ADRB1,ADRB2,ADRB3,AKR1B1,AQP1,ATP1A1,AVPR2,CYP11B2,ECE1,KCNMA1,MME,MMP12,NOS2,PIK3CG,PPARA,SLC22A6 |
| 9 | Regulation of muscle contraction | Group17 | ACE2,ADORA1,ADORA2A,ADORA2B,ADRA1D,ADRA2C,ADRB1,ADRB2,ADRB3,AKT1,ATP1A1,AVPR2,CACNA2D1,DBH,EGFR,KCNH2,KCNMA1,P2RX1,PIK3CG,PPARA,PTGS2,SCN5A,SERPINE1,SLC6A4,STAT3,TNFRSF1A |
| 10 | Xenobiotic metabolic process | Group16 | ABCB11,ABCC2,ACE,ACE2,AHR,AKR1B1,AKT1,ATP1A1,CBR3,CYP11B1,CYP11B2,CYP1A1,CYP1A2,CYP2B6,CYP2C8,CYP2C9,CYP2D6,CYP3A4,CYP3A5,CYP3A7,ECE1,EGFR,ERBB2,ESR1,KCNH2,MME,MMP12,NQO1,NR1I2,PIK3CG,PIK3R1,PPARA,PPARD,PPARG,PTGS1,SLC6A2,SOAT1,SRD5A2,TTR,UGT1A1 |
