# Supplementary material for: Integrating Pharmacokinetics Study, Network Analysis, and Experimental Validation to Uncover the Mechanism of Qiliqiangxin Capsule Against Chronic Heart Failure
Source: Front Pharmacol. 2019 Sep 18;10:1046. doi: 10.3389/fphar.2019.01046 (PMC6759796; doi:10.3389/fphar.2019.01046)
Supplement: Supplementary file 4 [file Table_4.doc]

**SUPPLEMENTARY TABLE S4 ׀** The top 10 representative pathways among 120 candidate targets.

| **No.** | **Function** | **Groups** | **Group genes** |
| --- | --- | --- | --- |
| 1 | Interleukin-4 and 13 signaling | Group17 | ABCB1|ABCC2|ABCG2|AHR|AKT1|ALOX15|AQP1|CBR3|COX1|CYP1A1|CYP1A2|CYP3A4|EGFR|ERBB2|ESR1|ESR2|IKBKG|LCN2|LGALS3|MMP1|MMP12|MMP2|MMP3|MMP9|NFE2L2|NOS2|NOX4|NQO1|NR4A1|PIK3CG|PIK3R1|PIM1|PPARD|PTGS2|PTK2B|SERPINE1|SLC6A2|SLC6A4|STAT3|TNFRSF1A|UGT1A1|VEGFA|XDH |
| 2 | Phase I - functionalization of compounds | Group16 | ABCB1|ABCC1|ABCC2|ADRB1|AHR|AKR1B1|ALDH2|ALOX15|CBR3|CYP11B1|CYP11B2|CYP1A1|CYP1A2|CYP2B6|CYP2C8|CYP2C9|CYP2D6|CYP3A4|CYP3A5|CYP3A7|ECE1|ESR1|NQO1|PPARA|PPARD|PTGS1|PTGS2|STAT3|UGT1A1 |
| 3 | Drug induction of bile acid pathway | Group15 | ABCB1|ABCB11|ABCC2|AKR1B1|ALB|AVPR2|CYP11B1|CYP11B2|CYP3A4|NR1I2|PPARA|SLC15A1|SLC15A2|SLC22A2|SLC22A6|SLC22A8|SLC6A2|SLCO1A2|SLCO1B1|SLCO1B3|SLCO2B1|SRD5A2 |
| 4 | Xenobiotics | Group14 | ABCB1|ABCB11|ABCC1|ABCC2|ABCG2|AHR|AKR1B1|ALB|AVPR2|CYP11B1|CYP11B2|CYP1A1|CYP1A2|CYP2B6|CYP2C8|CYP2C9|CYP2D6|CYP3A4|CYP3A5|CYP3A7|NR1I2|PPARA|PPARD|PPARG|SLC6A2|SLCO1B1|SLCO1B3|SLCO2B1|UGT1A1 |
| 5 | Tamoxifen metabolism | Group13 | AHR|AKR1B1|ALDH2|CYP11B1|CYP11B2|CYP1A1|CYP1A2|CYP2B6|CYP2C8|CYP2C9|CYP2D6|CYP3A4|CYP3A5|CYP3A7|EGFR|NFE2L2|NQO1|UGT1A1 |
| 6 | Reversible hydration of carbon dioxide | Group12 | AQP1|CA1|CA12|CA14|CA2|CA3|CA4|CA7 |
| 7 | Electron transport chain | Group11 | ATP5A1|COX1|COX2|COX3|CYTB|ND1|PPARD|SDHB|SDHD |
| 8 | Adrenoceptors | Group09 | ADRA1D|ADRA2C|ADRB1|ADRB2|ADRB3 |
| 9 | Adenosine P1 receptors | Group10 | ADORA1|ADORA2A|ADORA2B |
| 10 | ACE inhibitor pathway | Group08 | ACE|ACE2|CYP11B2|MME |
